# DNA Metabarcoding Reveals Broad Presence of Plant Pathogenic Oomycetes in Soil From Internationally Traded Plants

**DOI:** 10.3389/fmicb.2021.637068

**Published:** 2021-03-25

**Authors:** Simeon Rossmann, Erik Lysøe, Monica Skogen, Venche Talgø, May Bente Brurberg

**Affiliations:** ^1^Division of Biotechnology and Plant Health, Norwegian Institute of Bioeconomy Research (NIBIO), Ås, Norway; ^2^Department of Plant Sciences, Norwegian University of Life Sciences (NMBU), Ås, Norway

**Keywords:** baiting, environmental DNA, *Globisporangium*, *Phytophthora*, plant pathogens, *Pythium*

## Abstract

Plants with roots and soil clumps transported over long distances in plant trading can harbor plant pathogenic oomycetes, facilitating disease outbreaks that threaten ecosystems, biodiversity, and food security. Tools to detect the presence of such oomycetes with a sufficiently high throughput and broad scope are currently not part of international phytosanitary testing regimes. In this work, DNA metabarcoding targeting the internal transcribed spacer (ITS) region was employed to broadly detect and identify oomycetes present in soil from internationally shipped plants. This method was compared to traditional isolation-based detection and identification after an enrichment step. DNA metabarcoding showed widespread presence of potentially plant pathogenic *Phytophthora* and *Pythium* species in internationally transported rhizospheric soil with *Pythium* being the overall most abundant genus observed. Baiting, a commonly employed enrichment method for *Phytophthora* species, led to an increase of golden-brown algae in the soil samples, but did not increase the relative or absolute abundance of potentially plant pathogenic oomycetes. Metabarcoding of rhizospheric soil yielded DNA sequences corresponding to oomycete isolates obtained after enrichment and identified them correctly but did not always detect the isolated oomycetes in the same samples. This work provides a proof of concept and outlines necessary improvements for the use of environmental DNA (eDNA) and metabarcoding as a standalone phytosanitary assessment tool for broad detection and identification of plant pathogenic oomycetes.

## Introduction

Oomycetes comprise some of the most notorious plant pathogens, such as *Phytophthora infestans*, the pathogen widely known for its central role in the Irish potato famine and a remaining concern in potato production; *Phytophthora ramorum*, damaging a range of wild and cultivated tree and shrub species; *Pythium ultimum*, causing damping off and root rot in a range of important crops like corn, soybean, and potato (Kamoun et al., 2015). Oomycetes form a taxonomically distinct group of eukaryotic microorganisms that shares some biological features with fungi (e.g., formation of spores and hyphae) but are phylogenetically distant from fungi. The large spectrum of environmental conditions and plant hosts that oomycetes thrive in is reflected in their phylogenetic diversity (Thines, 2014). *Phytophthora*, *Pythium*, and *Globisporangium*, which was recently branched off from *Pythium* (Uzuhashi et al., 2010), are among the phylogenetically most complex oomycete genera and encompass an ever-growing list of species, of which a large number are plant pathogens. Many oomycetes, including various *Phytophthora* and *Pythium* species, overwinter as resting spores (oospores or chlamydospores), and some may remain dormant for several years before resuming a reproductive state (Judelson and Blanco, 2005; Babadoost and Pavon, 2013). Human activity, such as trade with rooted plants, is the predominant pathway of oomycete spread over long, potentially global, distances (Ristaino and Gumpertz, 2000; Cushman and Meentemeyer, 2008; Jung et al., 2016). Soil is also commonly moved locally or over longer distances, e.g., during construction projects, when transplanting trees or bushes from plant nurseries, or as debris on apparel, equipment, and machines. Oomycetes may also spread as airborne (zoosporangia with zoospores) or waterborne (zoospores or resting spores in soil particles) spores, and some can be transported from infected to healthy plants *via* vectors like certain insects (Goldberg and Stanghellini, 1990; Jarvis et al., 1993; Judelson and Blanco, 2005). Considering in addition the longevity of resting spores, the international transport of plant pathogens in contaminated soil presents a major threat to ecosystems, biodiversity, and food security. The risk of catastrophic disease outbreaks caused by oomycetes is especially high when a non-native invasive species, or lineages within a species, are introduced to an ecosystem that is not adapted to it (Fisher et al., 2012; Díaz et al., 2019; Fones et al., 2020). This was likely the case for the recent epidemics caused by *Ph. ramorum* on tanoak (*Notholithocarpus densiflorus*) in the United States and larch (*Larix* spp.) in the United Kingdom (Fry and Goodwin, 1997; Grünwald et al., 2012).

Despite this, phytosanitary measures required in the international transport of plants are commonly limited to a visual inspection for symptoms and do not address oomycete pathogens in rhizospheric soil. A single large individual plant can have a root-soil clump of many kilograms, resulting in thousands of tons of soil being transported internationally. In Europe, routine testing of a limited number of random samples is only done for a few plant pathogenic *Phytophthora* species that are recommended for regulation as quarantine pests in the A1/A2 lists of the European and Mediterranean Plant Protection Organization (EPPO 2018). Moreover, only samples from symptomatic plant tissue are tested and rapid, reliable detection methods for soil samples are currently not available to inspectors. Consequently, detection schemes for oomycetes and other microbial plant pathogens that are not regulated as quarantine pests are not established in international transport of plant material with soil in European countries.

In Norway, a national surveillance program was instated in 2018 and 2019 by the Norwegian Food Safety Authority to analyze for *Phytophthora* spp., in random samples of soil from imported woody ornamental plants, i.e., soil attached to the plants or as loose debris in shipping containers. Sequencing the ribosomal internal transcribed spacer (ITS) region of isolates after enrichment identified 19 distinct *Phytophthora* species from 231 soil samples (Talgø et al., 2019; Pettersson et al., 2020). Sequencing the ITS region of isolates provides reliable identification of oomycetes, but is resource intensive, as it requires DNA isolation from pure cultures before sequencing the marker region. To enrich plant pathogenic oomycetes before isolation, so called “baiting” is often employed. In this approach, healthy leaves or other parts of susceptible plants (e.g., *Rhododendron* spp.) are used as bait for oomycetes (e.g., Telfer et al., 2015). If the bait tissue develops symptoms like spots or lesions (appearing water soaked or necrotic), oomycetes can be isolated from the margin between diseased and healthy tissues. This approach has proven useful for analysis of both soil and water samples (e.g., Roberts et al., 2005; Shrestha et al., 2013). As an alternative, next-generation sequencing of short conserved DNA regions, so called “metabarcoding” or, more generally, “amplicon sequencing,” may be used to derive the identity of present oomycetes directly from environmental DNA (eDNA). This approach offers detection and identification of a wide variety of oomycetes and other groups of organisms directly from soil and other sources without requiring prior enrichment and laborious isolation of pure cultures. In the context of phytosanitary measures, establishing reliable metabarcoding routines could drastically improve the scope of controlling for stowaways in international transport of plants, and reduce the threat alien invasive species pose to plant-based industries and endemic vegetation.

In this work, metabarcoding was employed as a tool to detect and identify oomycetes present in soil samples before and after enrichment. The investigated samples comprised soil imported to Norway attached to roots of ornamental trees and shrubs. A particular focus was the presence and dynamics of potentially plant pathogenic oomycete genera and species, such as *Phytophthora* spp., and *Pythium* spp./*Globisporangium* spp. The reproducible analysis workflow for Illumina paired-end oomycete ITS1 metabarcodes used in this work is applicable to future work and can be modified to accommodate additional primer sets. The following hypotheses were tested:

*Hypothesis* 1: Internationally transported soil attached to plants contains potentially non-native, plant pathogenic oomycetes.
*Hypothesis* 2: Metabarcoding of the ITS1 region provides reliable detection and identification of oomycetes in soil samples.

Hypothesis 1 was tested by taxonomical and functional classification into potentially plant pathogenic genera for all oomycetes detected by metabarcoding in imported soil samples. Hypothesis 2 was tested by comparing detection and taxonomic identification by metabarcoding of the ITS1 region to isolates obtained through the traditionally used enrichment by baiting.

## Materials and Methods

### Sampling

A total of 73 soil samples were collected from three major entry points for plant import to Norway in 2018. All the soil sampling *in situ* was performed by regional inspectors from the Norwegian Food Safety Authority. The samples, each approximately 1 L, mainly originated from the root zone of woody ornamental plants, while a few constituted debris that had accumulated in the transport containers. A list of the samples with the exporting country, Norwegian region they were collected in, and the host plants are given in Supplementary Table S1.

### Enrichment of Oomycetes and Identification of Isolates

Oomycetes were enriched by baiting with asymptomatic leaves of *Rhododendron* cv. “Cunningham’s White” from greenhouse-grown plants. In preparation for baiting, each soil sample was homogenized in deionized water in suitable plastic trays. After a day, when sediments had settled, leaves were placed on the water surface with the abaxial side down, and incubated for up to a week at room temperature (~20°C). Where water soaked or necrotic lesions developed, sections from the margin between diseased and healthy tissue were dissected (pieces of approx. 0.5 cm^2^) using sterile scalpels. The leaf pieces were placed on P_10_ARPH agar (17 g corn meal agar in 1 L water; with addition of 250 mg ampicillin, 100 mg pentachloronitrobenzene, 35 mg hymexazol, 10 mg pimaricin, 10 mg rifampicin, and 1 ml dimethylsulphoxide), which is selective for *Phytophthora* species.

The agar plates were incubated at room temperature (~20°C) and checked daily for the presence of colonies. Hyphae resembling *Phytophthora* spp., were transferred to potato dextrose agar (PDA) plates to achieve pure cultures. The isolates were identified by sequencing their complete ribosomal ITS DNA region. In brief, DNA of cultured oomycetes was isolated using the DNeasy Plant Mini kit (Qiagen) according to the manufacturer’s instructions. The ITS region was amplified using the ITS5 and ITS4 primers (Supplementary Table S2) as described by White et al. (1990). The PCR products were submitted to Eurofins (Germany) for Sanger sequencing. Raw sequences were trimmed, assembled, and manually checked, and the final sequences were used to support identification of the isolates based on searches in public databases (GenBank and BOLD Systems).

### Metabarcode Sequencing

From each 1 L soil sample, 50 ml was sub-sampled for metabarcode analysis before and after enrichment. The sub-samples were homogenized, and total eDNA was isolated from 250 mg soil per sub-sample using the DNeasy PowerSoil kit (Qiagen). The 250 mg fractions of each sub-sample were chemically lysed in buffer C1 (DNeasy PowerSoil kit) for 10 min before mechanical homogenization using beads in a FastPrep-24 homogenizer (MP Biomedicals) for 2 × 45 s, speed 5.0. After homogenization and centrifugation, DNA isolation from the supernatant was automatized in a QIAcube (Qiagen) using the manufacturer’s PowerSoil protocol. Indexed libraries for paired-end sequencing of the ITS1 metabarcoding region were generated using a one-step PCR with indexed Oomycete specific primers from Agler et al. (2016) (see Supplementary Table S2). After amplification, the DNA concentrations of the PCR products were measured using a Qubit 2.0 Fluorometer (Invitrogen), and 100 ng DNA per sample was used for library generation. The libraries were purified twice using the MinElute PCR Purification Kit (Qiagen), the DNA concentration measured again using the Qubit Fluorometer, and integrity of the DNA was confirmed in the 2100 Bioanalyzer (Agilent). Sequencing was performed on an Illumina MiSeq System using MiSeq Reagent Kit v3 (600-cycle) chemistry (Illumina), with 15 and 20 pM of the libraries in two separate runs together with 10% PhiX control and sequencing primers. In addition to the soil samples, five replicates of a positive control containing DNA from 12 oomycete species and 11 fungal species were included in the libraries for both sequencing runs. A list of the species included in the positive control as well as the resulting amplicon sequence variants (ASVs) is given in Supplementary Table S1 (Sheet 2).

### Bioinformatics Analysis

Reads were automatically demultiplexed on the MiSeq system by the included MiSeq Reporter software, and the generated fastq files were used in downstream analysis. Adapters in the 3' region were removed from reads using “cutadapt,” based on the sequence of the 3' primers and a minimum match length of 15 bp (Martin, 2011). The DADA2 pipeline was used to derive ASVs, largely following Callahan et al. (2016). In short, reads were quality filtered using the “filterAndTrim” function; DADA2 was trained on the error rates for each of the two sequencing runs using the “learnError” function; reads were dereplicated using the “derepFastq” function; error rates were inferred using the “dada” function; paired reads were merged into a single sequence using the “mergePairs” function; the ASV table was generated using the “makeSequenceTable” function; samples “mergeSequenceTables” function; and chimeras were removed using the “removeBinerasDenovo” function.

The taxonomy used in this work was derived from a BLAST search against a local copy of the complete NCBI nucleotide database (nt, version 5), last updated from the FTP server in January 2020 (Tao et al., 2011). BLAST+ commands and derivation of the complete lineage for BLAST hits are documented in the “Blast_lineage_headers” R-Markdown document in the “Additional scripts” subdirectory of the GitLab repository under https://gitlab.nibio.no/simeon/oomycete-metabarcoding-supplementary.

Detailed parameters used in the DADA2 pipeline are found in Supplementary Material 2 and a full documentation of the data analysis contained in this work, mainly performed using the Phyloseq and tidyverse packages, are given in Supplementary Material 3. DADA2 and downstream data analysis scripts were last run in RStudio version 1.3 under R version 4.0; packages used, and their versions are documented in Supplementary Material 3.

## Results

### Metabarcoding Overview

DNA from the rhizospheric soil and debris samples of imported plants was sequenced in two Illumina MiSeq runs (with 20 and 45 samples, respectively). Subsamples before and after enrichment were always sequenced in the same run. The run with fewer samples produced more reads per sample on average, which was expected due to more available space in the flow cell (Supplementary Figure S1). Positive controls (a mix of oomycete species, five replicates per flow cell) included with both flow cells showed even and equivalent amplification across sequencing runs (Supplementary Figure S2).

After quality filtering and defining ASVs, the samples and controls contained ~35,000 ASV counts on average. Samples with more than 5,000 ASV counts were retained for analysis (Supplementary Figure S1).

Of 146 soil sub-samples initially taken in for analysis, (73 before enrichment, 73 after enrichment), 128 sub-samples were sequenced (63 before enrichment, 65 after enrichment), the remainder was disqualified due to insufficient PCR product during library generation. After processing, 58 samples achieved sufficient sequencing depth before and after enrichment (116 sub-samples total), while six samples yielded usable results for only one of the sampling points, for one sample, both sub-samples had to be discarded due to insufficient sequencing depth. As a result, 64 samples were analyzed with full or partial sub-sample coverage and renumbered as S01–S64. A full tabular overview of the samples, processing notes for discarded samples and metadata are provided in Supplementary Table S1. A full tabular overview of all ASVs with their taxonomy and ASV counts for each sample is provided in Supplementary Table S3.

### Oomycetes in eDNA

**Figure 1:**
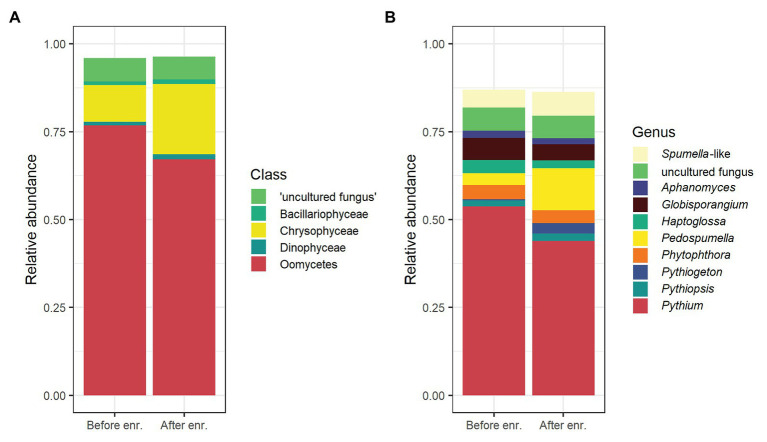
Overview of the most abundant organisms in rhizospheric soil samples detected by oomycete internal transcribed spacer (ITS) metabarcoding, means of all samples. Relative abundance of amplicon sequence variants (ASVs) belonging to the five most abundant taxonomic classes **(A)** and the 10 most abundant genera **(B)** before (“Before enr.”) and after enrichment (“After enr.”). In **(B)**, *Globisporangium*, *Pythium*, and *Phytophthora* are highlighted in bordeaux, red, and orange, respectively, while *Aphanomyces* and *Phytiogeton* are colored in shades of purple/dark blue. Supplementary Figure S3 shows a per-sample overview for the top five classes and top 10 genera.

A total of 5,195 ASVs were identified in the samples, and 1,832 were classified as oomycete sequences, representing ~72% of all reads. This relative dominance of oomycete ASVs was more pronounced in the untreated soil samples than the samples after enrichment (Figure 1). The five most abundant taxonomic classes, in order of abundance, were Oomycetes, Chrysophyceae, “uncultured fungi,” Bacilliarophyceae, and Dinophyceae, representing ~96% of all reads (Figure 1). The 10 most abundant genera covered approx. 87% of all reads. The most abundant genus was *Pythium* (mean 49%), *Globisporangium* and *Phytophthora* were also among the 10 most abundant genera (mean 6% each). The distribution of the top five classes and top 10 genera per sample is shown in Supplementary Figure S3. The number of oomycete ASVs and their relative proportion per sample is given in Table 1.

**Table 1:** Overview of ASVs annotated as oomycetes and potentially plant pathogenic genera in soil samples before and after enrichment, and isolates after enrichment.

				# Oomycete ASVs		% Oomycete ASVs		# PP ASVs		% PP ASVs		
Soil	Type	Country	SeqRun	Before	After	Before	After	Before	After	Before	After	Isolates
S01	Rhiz.	NL	A	83	104	86.8	88.4	55	66	75.1	81.7	–
S02	Rhiz.	NL	A	66	64	85.4	95.5	37	34	77.7	87	2
S03	Debris	NL	A	107	101	99.1	97.2	80	62	96.7	28.5	1
S04	Rhiz.	NL	A	80	101	93.3	80.8	56	72	76.5	66.4	1
S05	Rhiz.	NL	A	75	77	67.3	71.4	58	58	57.1	63	–
S06	Rhiz.	DK	A	19	39	36.9	61.1	11	20	25.6	44.7	–
S07	Rhiz.	NL	A	49	74	97.2	60.6	29	55	81	47.6	–
S08	Rhiz.	NL	A	72	78	96.4	97.6	42	46	73.9	92.1	–
S09	Rhiz.	NL	A	-	65	-	96.7	-	31	-	35.1	1
S10	Rhiz.	NL	A	-	20	-	42.2	-	10	-	38.4	–
S11	Rhiz.	DE	A	19	17	78.9	20.1	11	9	40	13.9	–
S12	Rhiz.	NL	A	-	68	-	90	-	52	-	74.1	–
S13	Rhiz.	IT *via* DK	A	16	57	45	74.4	12	39	44	68.6	–
S14	Rhiz.	NL	A	55	73	98.6	95.2	36	48	93.7	86.5	–
S15	Rhiz.	NL	A	38	43	40.4	58.8	31	36	35.5	55.5	–
S16	Rhiz.	NL	A	38	53	93.3	84.6	25	40	77.1	74.6	–
S17	Rhiz.	NL	A	15	-	91.1	-	14	-	87	-	–
S18	Rhiz.	NL	A	10	-	43.7	-	9	-	43.1	-	–
S19	Rhiz.	NL	A	53	58	73	58.9	38	36	52.8	51.4	–
S20	Rhiz.	NL	B	27	15	74.7	13.9	12	9	49	6.9	–
S21	Rhiz.	NL	B	51	36	72.1	58.8	10	15	4.2	12.2	–
S22	Rhiz.	NL	B	36	22	66.1	65.9	26	12	57.5	50.3	–
S23	Rhiz.	DK	B	53	57	73.4	82.5	32	33	54.7	51.4	–
S24	Rhiz.	NL	B	34	46	74.3	74.2	28	35	63.5	65.6	–
S25	Rhiz.	DE	B	47	25	90.9	74.6	25	16	62.5	32.5	–
S26	Rhiz.	IT *via* DK	B	29	34	97.9	17	17	25	95.1	15.5	–
S27	Rhiz.	NL	B	-	45	-	69.1	-	34	-	57.8	–
S28	Rhiz.	NL	B	22	49	74.2	58	13	33	59.6	45.3	–
S29	Rhiz.	NL	B	72	51	92.4	86.8	57	42	78.6	78.5	2
S30	Rhiz.	NL	B	24	59	89.5	59.7	15	35	61.2	32.8	1
S31	Rhiz.	NL	B	37	25	58.3	59.2	24	20	39.7	39.6	–
S32	Rhiz.	NL	B	36	30	97.1	84.8	24	14	91.1	71.3	–
S33	Rhiz.	PL	B	19	12	6.7	10.1	6	4	0.9	0.5	–
S34	Rhiz.	NL	B	32	49	73.4	43.1	24	37	57.3	38.3	–
S35	Rhiz.	NL	B	52	57	99	97.7	45	44	96.1	94.2	–
S36	Rhiz.	NL	B	47	46	89.8	62.2	27	23	81	41	–
S37	Rhiz.	NL	B	78	77	97.6	94.2	62	60	96	80.4	–
S38	Rhiz.	NL	B	53	21	79	76.9	32	16	58.9	70.4	–
S39	Debris	NL	B	24	24	75	84.8	20	15	70.4	53.1	–
S40	Rhiz.	DK	B	51	64	89.8	77.3	32	39	80.1	66.9	–
S41	Rhiz.	SE	B	59	31	85.9	53.8	47	23	79.4	41.8	–
S42	Rhiz.	NL	B	21	13	6.2	6.9	11	5	3.3	2.3	–
S43	Rhiz.	DK	B	71	61	94	93.8	59	49	84.7	81.2	–
S44	Rhiz.	NL	B	19	7	67.3	39.6	11	3	48.3	37.1	–
S45	Rhiz.	NL	B	26	17	13.1	17	5	5	2.4	1.9	–
S46	Rhiz.	DE	B	49	21	83.4	79.9	32	14	61.5	70.3	1
S47	Rhiz.	DE	B	51	48	94.6	83.9	34	36	57.2	70.8	–
S48	Rhiz.	DK	B	46	67	96	96.7	35	48	90.5	81.4	1
S49	Rhiz.	IT *via* DK	B	26	26	90.9	83.4	20	20	77.7	68.5	–
S50	Rhiz.	IT *via* DK	B	35	47	97.8	79.4	25	32	96.1	72.4	–
S51	Rhiz.	IT *via* DK	B	35	61	65.3	51	25	50	22.7	32	1
S52	Rhiz.	DE	B	32	28	76	71.8	23	20	31.1	33.3	–
S53	Rhiz.	DK	B	41	23	84.3	70.9	31	16	76	54.8	–
S54	Rhiz.	DK	B	44	29	85.5	61.4	37	24	77.8	55.9	–
S55	Rhiz.	NL	B	31	22	83.6	77.4	18	17	55.8	64.9	–
S56	Rhiz.	DK	B	22	32	95.4	64.3	18	22	83.3	52	–
S57	Rhiz.	NL	B	16	21	57.3	26.2	9	13	9.7	13.9	1
S58	Rhiz.	IT *via* DK	B	28	22	94.3	69.2	21	15	31.8	25.6	1
S59	Rhiz.	DE	B	33	45	77.6	27.3	19	24	52.8	10	–
S60	Rhiz.	DE	B	23	60	61.6	73.3	17	40	59.4	61.4	–
S61	Rhiz.	DK	B	52	43	97.1	95.4	36	31	86.9	87.9	–
S62	Debris	DK	B	33	23	97.5	80.8	23	12	71	3.5	–
S63	Rhiz.	SE	B	28	19	15.5	48.1	19	13	6.7	16.3	–
S64	Rhiz.	IT *via* DK	B	67	44	87.6	83.5	41	25	75.8	59.8	–

For each soil sample included in the final analysis (S01–S64) the sample type (rhizospheric soil = “Rhiz.” or “Debris”), the exporting country (two letter country code) and the sequencing run (“SeqRun”) are given. The number of ASVs (#) and their relative abundance in the sample (in percent) is given for ASVs annotated as oomycetes and ASVs annotated as potentially plant pathogenic genera (“PP”) before enrichment (“Before”) and after enrichment (“After”). The number of isolates recovered from the soil samples after enrichment is also provided. Missing values due to samples that were discarded because of low read count or unsuccessful amplification are indicated by “-”.

### Abundance of Putative Plant Pathogenic Oomycetes

**Figure 2:**
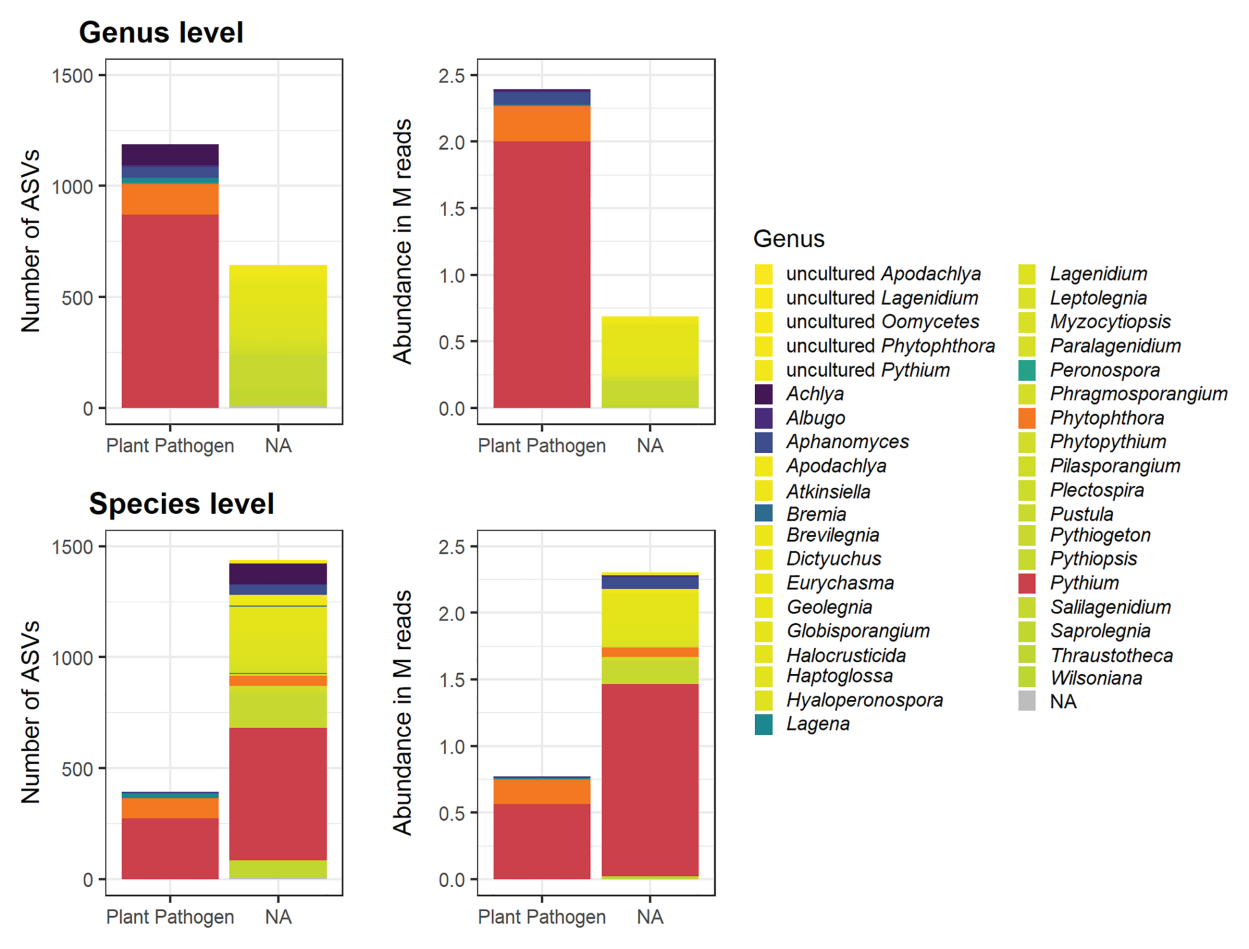
Oomycete ASVs in rhizospheric soil samples detected by ITS metabarcoding, and their classification in the FUNGuild database on a genus and species level. The number of ASVs with a given designation is shown in the left panels and the total abundance of these ASVs in million (M) reads is shown in the right panels. In both the genus and species level plots, the bars are colored according to the genus assignment. *Pythium* and *Phytophthora* are highlighted in red and orange, respectively. Other genera that were assigned to the “plant pathogen” guild and species belonging to them are colored in shades between dark purple and dark green. Oomycete genera that were not assigned any guild in the FUNGuild database (“NA”) and the species belonging to them are colored in shades between yellow and lime.

To determine the plant pathogenic potential of the identified oomycete ASVs, their taxonomic classification was matched against the FUNGuild database that categorizes organisms into guilds according to their ecological role (Nguyen et al., 2016). While the primary focus of the database is on fungi, it contains entries on several other organism groups, including oomycetes. Of the 1,832 oomycete ASVs detected in this work, 1,188 were annotated as probable members of the “plant pathogen” guild on a genus level (Figure 2). On a species level, 395 ASVs were annotated as probable plant pathogens. The remaining ASVs did not correspond to entries in the FUNGuild database and were therefore not assigned a guild. Since the identification of ASVs to a species level is not possible based on metabarcoding of a single marker with high confidence, the guild classification on a genus level was used as the basis of further analysis. These ASVs are referred to as “potentially plant pathogenic” (abbreviated as “PP”) on the genus level below. It is important to note that *Pythium*, the most abundant genus, was annotated as potentially plant pathogenic in FUNGuild, but more than half of the reads belonging to *Pythium* ASVs were not assigned a guild on a species level (Figure 2). Likewise, a large number of species belonging to other genera were not assigned any guild, even if the genus they belonged to was classified as a plant pathogen, either because the species were not found in the database or because they lacked guild assignments on the species level (Figure 2). The number of PP ASVs and their relative proportion per sample is given in Table 1.

### Decrease of Potentially Plant Pathogenic Oomycetes During the Enrichment

**Figure 3:**
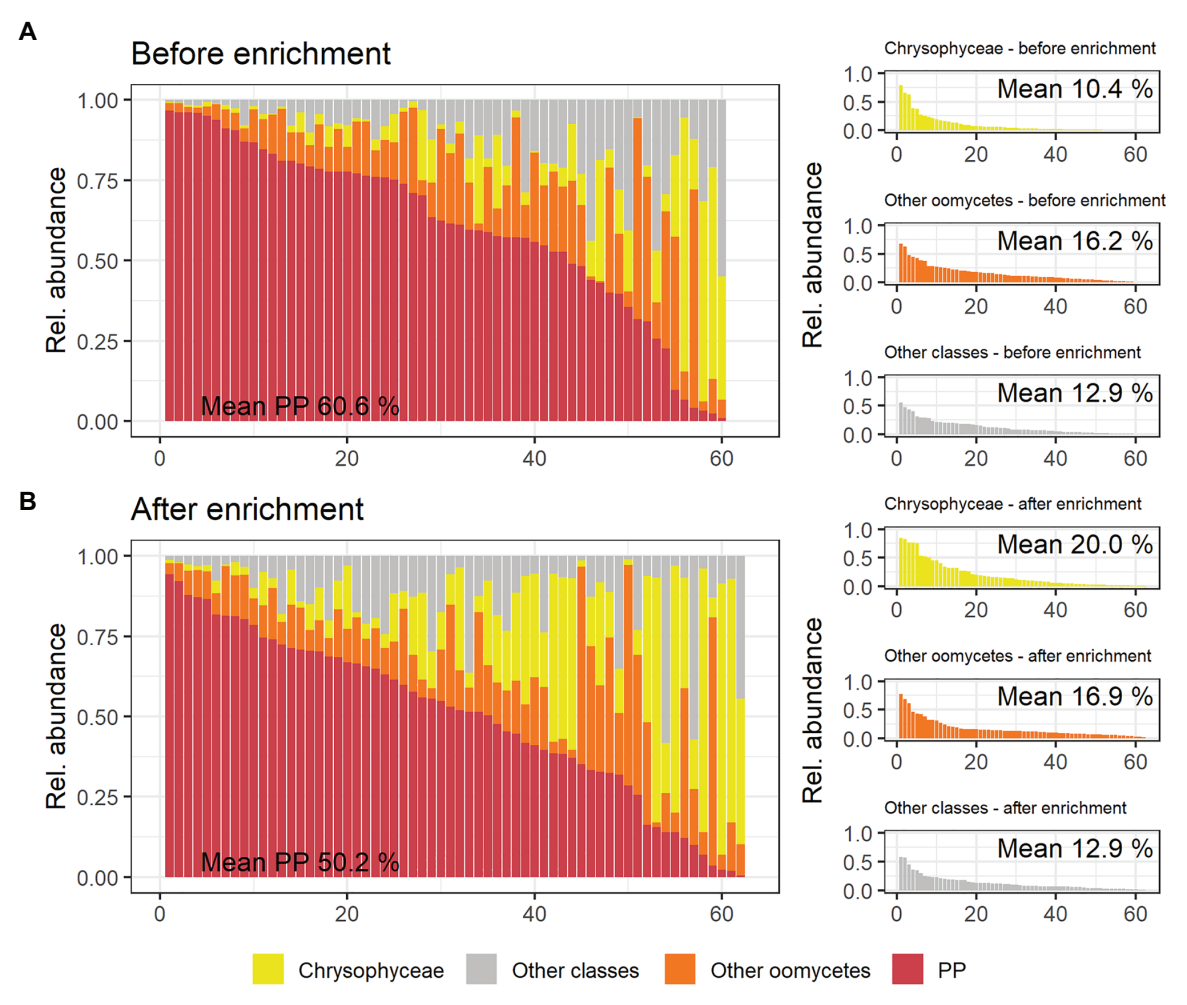
Overview of taxonomic classes of interest and their relative abundance per rhizospheric soil sample detected by metabarcoding using the oomycete ITS1 marker. **(A)** shows the relative abundances in soil samples before enrichment, **(B)** shows corresponding results in soil after enrichment. The large panels show relative abundances of plant pathogenic oomycete genera (PP, red) per sample ranked from the sample with the highest to the lowest relative abundance. The relative abundances of other Oomycetes, Chrysophyceae, and all other classes (including not taxonomically assigned ASVs) are shown in orange, yellow, and gray, respectively. These groups are also shown as separate, smaller graphs in the right panels of **(A)** and **(B)**, with the mean percentages of reads belonging to the different taxonomic classes given in each panel.

Mean relative abundance of plant pathogenic oomycetes significantly (*p* = 0.03) decreased from 60.6% in untreated soil to 50.2% in soil after enrichment (“PP” in Figure 3). Unlike plant pathogenic oomycetes, the relative abundance of oomycetes belonging to other genera did not significantly change during enrichment (16.2 and 16.9% mean). The relative decrease of plant pathogenic oomycete genera was largely caused by a significant (*p* = 0.008) increase in mean relative abundance of golden-brown algae from the class Chrysophyceae, which increased from 10.4% in untreated soil to 20% in enriched soil. This observed shift in mean relative abundance was facilitated by an absolute increase in Chrysophyceae reads, not a decrease in reads from plant pathogenic oomycetes (Supplementary Figure S4). Reads from classes other than Oomycetes and Chrysophyceae, including taxonomically not assigned reads, were not significantly impacted by the enrichment process. The full results of the statistical analysis of mean relative abundances can be found in Supplementary Table S4. Table 1 shows that the significantly lower abundance of potentially plant pathogenic oomycetes after enrichment observed for mean values was not observed in all samples equally.

### ASVs Corresponding to Isolated Oomycetes Recovered in Metabarcoding

**Figure 4:**
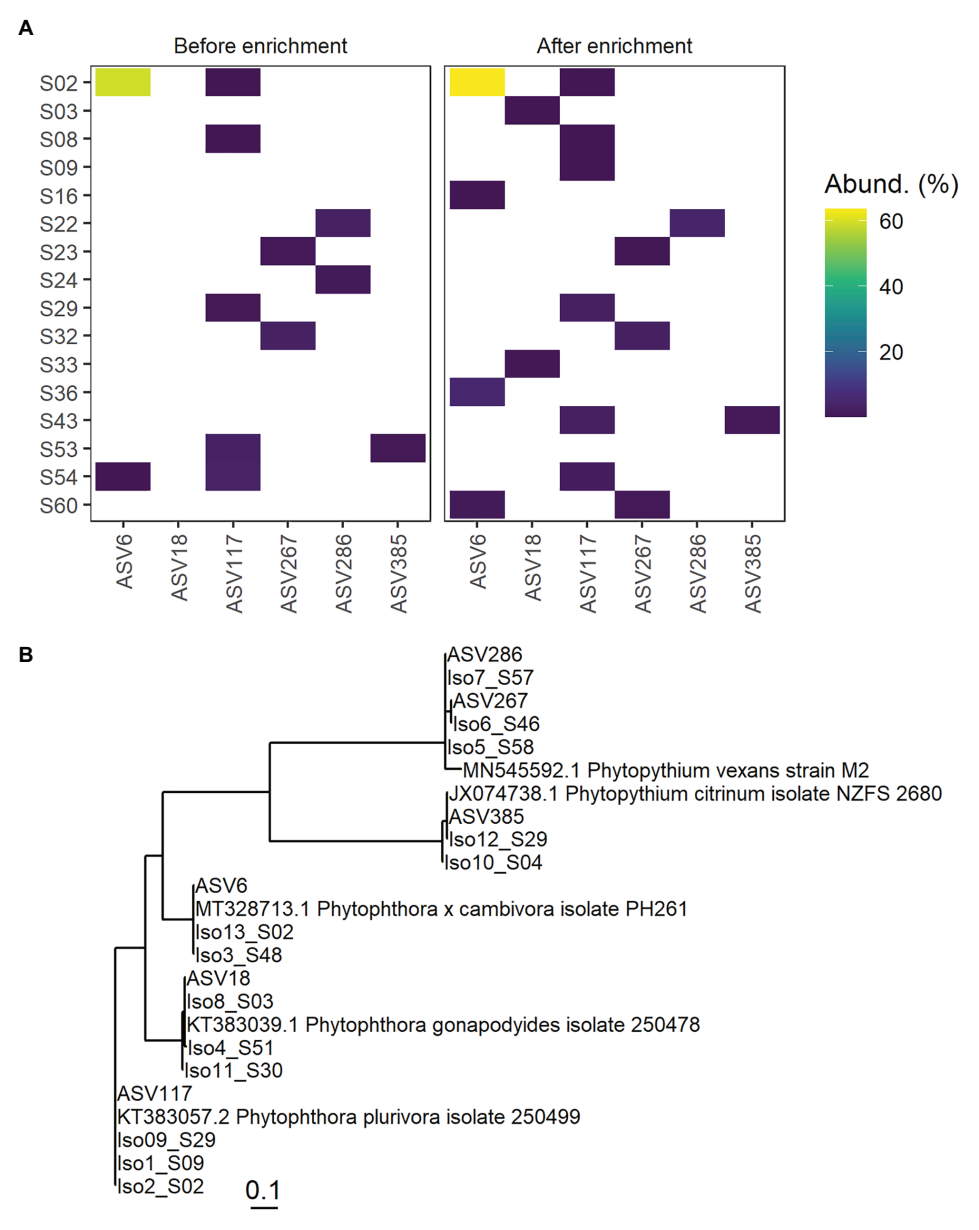
Detection of ASVs corresponding to oomycetes isolated from rhizospheric soil samples. In **(A)**, the left panel shows ASV detection by metabarcoding before enrichment, the right panel after enrichment. ASVs are given on the *x*-axis and soil samples on the *y*-axis. The relative abundance of ASVs in percent of the whole soil sample is indicated on a continuous color scale, no color was assigned if the abundance was 0%. **(B)** shows a phylogenetic tree based on ITS-sequences of isolates, corresponding ASVs, and publicly available sequences for identified strains. Represented in a Neighbor Joining tree fitted with a General time reversible model with discrete Gamma distribution and evolutionarily invariable sites (GTR + G + I). The tree’s scale (substitutions per site) is indicated on the bottom.

Thirteen isolates recovered from 11 soil samples after enrichment were identified using traditional ITS sequencing and used to assess the accuracy of metabarcoding. The isolates and corresponding ASVs (>98% sequence similarity) were identified as *Phytophthora* and *Phytopythium* species by traditional ITS-sequencing and metabarcoding (Table 2). The corresponding ASVs were detected in multiple soil samples by metabarcoding and clustered phylogenetically with the isolate sequences and reference isolates (Figure 4). In some soil samples, ASVs corresponding to an isolate sequence were observed either only before or only after enrichment. Most ASVs that were highly similar to isolate sequences were detected at low abundance relative to other ASVs in the soil sample. An exception was ASV6 (*Phytophthora cambivora*) which was detected with a relative abundance of approx. 60% in soil sample S02 before and after enrichment. While all ASVs corresponding to isolates were detected in multiple soil samples, the ASVs were detected in the same soil samples as the isolate was obtained from in only five of 13 cases. The full lineage assigned to ASVs in the taxonomic pipeline and the number of times any ASV was observed in each soil sample is given in Supplementary Table S3.

**Table 2:** Thirteen isolates obtained from rhizospheric soil samples after enrichment, their species identities and corresponding ASVs recovered in metabarcoding.

Soil	Isolate ID	Identified as	Most similar ASV	ID%	ASV identified as
S02	2	*Phytophthora plurivora*	ASV117	98.4	*Phytophthora plurivora*
S02	13	*Phytophthora cambivora*	ASV6	100	*Phytophthora cambivora*
S03	8	*Phytophthora gonapodyides*	ASV18	100	*Phytophthora gonapodyides*
S04	10	*Phytopythium citrinum*	ASV385	98.5	*Phytopythium citrinum*
S09	1	*Phytophthora plurivora*	ASV117	100	*Phytophthora plurivora*
S29	9	*Phytophthora plurivora*	ASV117	100	*Phytophthora plurivora*
S29	12	*Phytopythium citrinum*	ASV385	100	*Phytopythium citrinum*
S30	11	*Phytophthora gonapodyides*	ASV18	99.1	*Phytophthora gonapodyides*
S46	6	*Phytopythium vexans*	ASV267	100	*Phytopythium vexans*
S48	3	*Phytophthora cambivora*	ASV6	100	*Phytophthora cambivora*
S51	4	*Phytophthora gonapodyides*	ASV18	99.6	*Phytophthora gonapodyides*
S57	7	*Phytopythium vexans*	ASV286	100	*Phytopythium vexans*
S58	5	*Phytopythium vexans*	ASV286	99.6	*Phytopythium vexans*

For each isolate, the soil sample it was recovered from (S01–S64), the species it was identified as based on the full ITS sequence, and the most similar ASV are given. Percent (ID%) refer to nucleotide similarity between these ASVs and corresponding isolate’s ITS sequence (obtained by Sanger sequencing) over the length of the ASV (approximately 170–270 bp).

## Discussion

### Presence of Potentially Plant Pathogenic Oomycetes in Imported Rhizosphere Soil

As described above, nucleotide reads were readily obtained from 128 rhizospheric soil samples using the ITS1 primers optimized for metabarcoding of oomycetes developed by Agler et al. (2016). An additional 18 samples were excluded from the analysis because they did not yield usable DNA or contained too few reads to be analyzed, likely due to unsuccessful processing, not an actual absence of oomycetes in those samples. Two oomycete isolates were obtained from one of these samples after enrichment, but DNA isolation and/or ITS1 PCR were unsuccessful both before and after enrichment. Since problems with DNA isolation and/or PCR amplification most often occurred for both sampling points of the same soils, these soil samples were either completely free of biomaterial targeted by the ITS1 primers, or, more likely, contained inhibitors that interfered with successful DNA isolation and/or PCR amplification. While this affected only a minority of samples (12%), improvements in the isolation and amplification procedure could likely remedy this, e.g., extra purification steps prior to PCR. The number of samples that were amplified successfully was sufficient to address the aims stated for this work, thus further attempts to amplify the remaining samples were not made. The integrity and evenness of the positive controls over two independent sequencing runs indicate that the PCR amplifications were uniform and reliable. Well-known issues in high throughput sequencing, such as index-hopping resulting in the incorrect assignment of sequences from the true sample to a different sample, or PCR amplification preference for certain ASVs due to primer bias are more likely to affect relative abundance estimates in samples with very few reads. Therefore, six samples with total ASV counts of less than 5,000 were excluded from the analysis. Overall, a large majority of samples yielded a sufficient number of high-quality reads (Supplementary Figure S1).

The most abundant ASVs were typically identified as originating from oomycetes, as expected for the employed primer set (Figure 1). *Phytophthora* and *Pythium* were among the 10 most abundant genera identified in the samples, with *Pythium* being most abundant (Figure 1B). The high prevalence of these two potentially plant pathogenic genera was likely not an effect of primer biases, since previous experiments using this set yielded primarily the oomycetes *Albugo* and *Hyaloperenospora* and far fewer *Pythium* and *Phytophthora* from plant material (Figure S6 in Agler et al., 2016).

Potentially plant pathogenic oomycetes, which are frequently species belonging to the *Phytophthora* genus and, to a lesser extent, *Pythium* species were of particular interest and represented a large part of the detected oomycetes (Figure 2). The classification based on the FUNGuild database proved to be a useful tool for automated annotation of this trait but was only reliable to a limited extent. The primary issue was that the FUNGuild database is primarily aimed at functional grouping of fungi, not oomycetes. The oomycete entries are mostly automated imports from the database entries of the American Phytopathological Society (APS) and not curated by humans. This may lead to some degree of false functional classification. The lack of focus on oomycetes in the FUNGuild database also means that some important genera and species have no entry there. This was the case for, e.g., *Globisporangium*, which was recently branched off from *Pythium* and inherited some of the most problematic plant pathogens formerly belonging to *Pythium*, such as the former *Py. irregulare*, *Py. sylvaticum*, and *Py. ultimum* (Uzuhashi et al., 2010). In the NCBI BLAST nucleotide database and corresponding taxonomic database used for the automatic taxonomy pipeline in this work, some but not all these former *Pythium* species, are correctly annotated as *Globisporangium*, meaning that there was only partial correspondence between the annotation obtained in this work and the FUNGuild database. Because of these issues, the FUNGuild database when used for oomycete functional classification may have produced a high rate of false negatives, where oomycete ASVs may actually belong to the plant-pathogens but were not assigned a guild. However, in cases where ASVs were classified as originating from plant pathogenic oomycetes, it is reasonable to assume that they were correctly annotated as such, meaning a likely low risk of false positives. The annotation provided by this method therefore offers a conservative functional classification on the genus level in this case. It is important to note that neither the NCBI nt database used for taxonomical annotation, nor the FUNGuild database used for functional annotation were optimal, but currently the best available tools. Alternatives, primarily curated ITS databases such as BOLD and UNITE were tested but performed worse for taxonomical assignment (higher amount of unassigned sequences and lower confidence assignments) due to their relative lack of diverse and well-annotated oomycete sequences (Ratnasingham and Hebert, 2007; Nilsson et al., 2018). For functional assignment, no suitable alternative databases were found. Future efforts to employ metabarcoding for oomycete detection and identification need to reevaluate the use of these resources and carefully consider whether better, curated databases are available or existing databases like UNITE have been amended sufficiently.

### Bias Introduced by Enrichment

The enrichment process significantly reduced the relative abundance of potentially plant pathogenic oomycetes in favor of Chrysophyceae (golden-brown algae). We expected oomycetes, such as *Phytophthora* spp., that are captured by the employed enrichment method to increase rather than decrease, because the biomass of such oomycetes should accumulate drastically. There are two likely causes that may explain the observed dynamics: first, the accumulation of plant pathogenic oomycetes may have been hyperlocalized to the leaves that were used for baiting without affecting the biomass of these oomycetes in the soil samples. Second, the conditions during the enrichment process, with the soil having been submerged in water for approximately a week likely favored the growth of Chrysophyceae, leading to an unintended relative increase of these organisms. Indeed, the absolute number of reads attributed to oomycetes from genera of interest in the soil remained relatively stable while mean Chrysophyceae reads more than doubled during enrichment (Supplementary Figure S4). While not a reliable measure of abundance, the absolute number of reads may allow some conclusions on the dynamics when comparing soil samples before and after enrichment since both were always sequenced on the same flow cell.

### Reliability and Usefulness of Metabarcoding in Detection

Amplicon sequence variants identical or highly similar to sequences obtained by ITS barcoding of *Phytophthora* and *Phytopythium* isolates were obtained by metabarcoding (Table 2; Figure 4). For the highly abundant ASV6 before and after enrichment in soil sample S02, a corresponding *Phytophthora cambivora* culture was isolated from that sample. *Phytophthora plurivora* isolates identical or highly similar in sequence to ASV117 were detected by metabarcoding in soil samples S02, S09, and S29 and isolated from those samples. *Phytophthora gonapodyides* was isolated from S03 and the identical ASV18 was detected in the same sample. For the remaining *Phytophthora* isolates and all *Phytopythium* species, the ASVs corresponding to isolates were not observed in the soil samples the isolates were found in. This was likely an effect of the small soil samples (250 mg from 50 ml) used for DNA isolation compared to the large total volume used in the enrichment (approx. 1 L). A hyperlocalization of the infecting oomycete at the leaves without a corresponding increase in the soil during enrichment may also have contributed to lack of ASV detection where isolates were found. To avoid an effect of potential hyperlocalization and get a better correlation with isolates, DNA could be isolated from the water used during enrichment, a higher soil volume should be used for DNA isolation and soil from the entire sample should be homogenized thoroughly before sampling for DNA isolation. In general, submerging soil in water for some time and subsequently filtering the water and extracting DNA from the filter may yield a more representative census of microorganisms in large volumes of soil, even if no enrichment is planned. The use of additional primer sets could also increase detection frequency further by balancing potentially less efficient amplification of some genera.

For identification purposes, traditional isolation-based approaches that employ Sanger sequencing of specific DNA marker regions like ITS have some advantages over metabarcoding in its current form, as they have fewer restrictions on sequence length or targeted region. Additionally, having access to isolates offers the possibility to study morphology, biochemical features, as well as pathogenicity. Purely DNA-based methods like metabarcoding cannot distinguish between DNA from dead and living organisms, although it is likely that DNA from dead organisms in the soil samples tested here would have been degraded by living microorganisms during transport of the plants, and/or during the enrichment. Metabarcoding as a means of simultaneous and sensitive detection and identification of oomycetes in soil will improve with additional primer sets, increased length of sequenced fragments, and dedicated databases for taxonomic and functional assignment. Despite some current shortcomings, metabarcoding showed itself to have several advantages in the detection of potentially plant pathogenic oomycetes, as it produced useful results from untreated soil, negating the need for potentially biased treatment and laborious isolation of pure cultures. It furthermore enabled the agnostic detection of a wide variety of *Phytophthora* and *Pythium*, two genera of high interest. Detection that does not take preconceived notions of relevance into account is important, because *Pythium* (~*Globisporangium*) species like *Py. ultimum*, *Py. irregulare*, and *Py. sylvaticum* are problematic plant pathogens, particularly on tree seeds and seedlings, that are often discarded or ignored in isolation-based screenings because they are less dangerous than *Phytophthora* species after the nursery stage.

While ASV-based processing yields sequences that can be regarded as proxies for unique organisms analogous to “operational taxonomical units” (OTUs), it is challenging to annotate these sequences with traditional taxonomical units on the lowest level, such as species or strains, since species affiliation can only be reliably determined from longer sequences and might include morphological or phenotypical characteristics. Despite stringent quality filtering, and although false-positive rates are lower than with comparable methods, false-positive ASVs may be derived from Illumina-reads due to sequencing errors regardless of the compensation for such errors in the data processing (Callahan et al., 2016). Furthermore, some *Phytophthora* species are identical or highly similar across the ITS region traditionally used for identification of isolates, increasing the chance of incorrect or ambiguous species assignment even when using a longer segment of the ITS region (Yang and Hong, 2018). Even so, annotation of ASVs down to the species level based on DNA sequence similarity is possible and was done in this work (Supplementary Table S3). However, genus level assignment of ASV taxonomy is more reliable than species level annotation and should be focused on when interpreting metabarcoding results without additional information, given the currently available databases. While the approach used in this work produced nearly complete taxonomic lineage annotation and good results, there is a need for a curated and balanced collection of oomycete reference sequences for taxonomic and functional annotation of ASVs. Unlocking the full potential of metabarcoding as an identification tool requires such sequence databases tailored to the organisms of interest (Dormontt et al., 2018; Richardson et al., 2018).

In summary, metabarcoding showed several advantages compared to isolation-based strategies: (1) Unbiased, sensitive, broad detection of all oomycetes; (2) For specific oomycetes, such as *Phytophthora* spp., metabarcoding can give near-complete detection of variants present in a sample, while isolating all variants present in a given sample with a high degree of certainty requires considerable effort, metabarcoding therefore has a lower theoretical false-negative detection rate in routine applications; (3) Shorter turn-around time from sampling to detection in soil without enrichment; (4) Increased amount of samples that can be processed per person-hour; and (5) Less rigid processing schedule as the samples can be frozen for prolonged periods between any of the steps without losing information. There are also major drawbacks of metabarcoding, some of which can be addressed in the future and some that are inherent to the method: (1) There is no definitive proof of the presence of live oomycetes without isolating them; (2) Short amplicon sizes make species identification unreliable, this may be addressed by amplifying with additional primer pairs or by increasing amplicon size with, e.g., PacBio sequencing technology; and (3) Databases for reliable taxonomic and functional identification of oomycetes are lacking, this should be addressed by increased efforts to create new databases and improve existing ones.

The relatively high abundance of *Pythium* and *Phytophthora* detected in this work implies wide-spread presence of considerable amounts of potentially plant pathogenic oomycetes in soil associated with the rhizosphere of woody ornamentals that are traded internationally. Our results therefore strengthen the first hypothesis stated in the introduction. The second hypothesis underlying this work was supported to a large degree by the results presented here, but further improvements on aspects of metabarcoding implementation are needed. Compared to the conventional approach that relies on enrichment and isolation of certain oomycete genera, metabarcoding detected and identified a far larger diversity of oomycetes from untreated soil and showed the growth of undesirable organisms during the enrichment process. With sufficient research data and further development of the underlying technology, metabarcoding can eventually become a valuable standalone tool for direct detection and identification of plant pathogenic oomycetes in phytosanitary control schemes.

## Data Availability Statement

The datasets presented in this study can be found in online repositories. All R code and files required to reproduce the analysis of results presented here can be found on the GitLab repository at: gitlab.nibio.no/simeon/oomycete-metabarcoding-supplementary. Raw read data is archived at the European Nucleotide Archive (ENA) under the accession number PRJEB40676 (www.ebi.ac.uk/ena/browser/view/PRJEB40676).

## Author Contributions

MB, EL, and VT conceived the ideas and designed the methodology. MS and VT collected the data. SR and EL analyzed the data. SR led the writing of the manuscript. All authors contributed to the article and approved the submitted version.

### Conflict of Interest

The authors declare that the research was conducted in the absence of any commercial or financial relationships that could be construed as a potential conflict of interest.

## Supplementary Material

Supplementary Table 1 Overview of all rhizospheric soil samples, whether they yielded DNA and sufficient PCR amplicons for sequencing, the read counts through the processing and associated metadata. The metadata for each numbered soil sample includes the countries from which the samples were imported, which plant species the soil was imported with and processing notes when samples were discarded. Sheet 2 contains information on the species used as positive controls and whether they were found in sequencing.

Supplementary Table 3 Occurrence of ASVs in the samples and their full taxonomy. For each ASV, the ASV count is given for all samples. The taxonomy derived from local BLAST against the NCBI nt database is provided on seven taxonomic levels (where available): Kingdom, Phylum, Class, Order, Family, Genus, Species.

Supplementary Material 2 Complete documentation of the R code used for the DADA2 pipeline. The code and outputs are compartmentalized in chunks for separate processes. The raw reads used as input for DADA2 are deposited on www.ebi.ac.uk/ena/data/view/PRJEB40676. Version information for R, RStudio and the libraries used is found at the bottom of the document. The R Markdown file is available on GitLab under gitlab.nibio.no/simeon/oomycete-metabarcoding-supplementary.

Supplementary Material 3 Complete data analysis documentation including a clear description of the input data, the code that was used to analyse it and the various outputs as well as version information for R, RStudio and the libraries used. The code and outputs are compartmentalized in chunks for separate processes. The data files used as input for the Phyloseq section and the R Markdown file are available on GitLab under gitlab.nibio.no/simeon/oomycete-metabarcoding-supplementary.
