# Supplementary material for: DNA Metabarcoding Reveals Broad Presence of Plant Pathogenic Oomycetes in Soil From Internationally Traded Plants
Source: Front Microbiol. 2021 Mar 25;12:637068. doi: 10.3389/fmicb.2021.637068 (PMC8027490; doi:10.3389/fmicb.2021.637068)
Supplement: Supplementary file 1 [file Data_Sheet_1.docx]

Supplementary Material

# Supplementary Figures


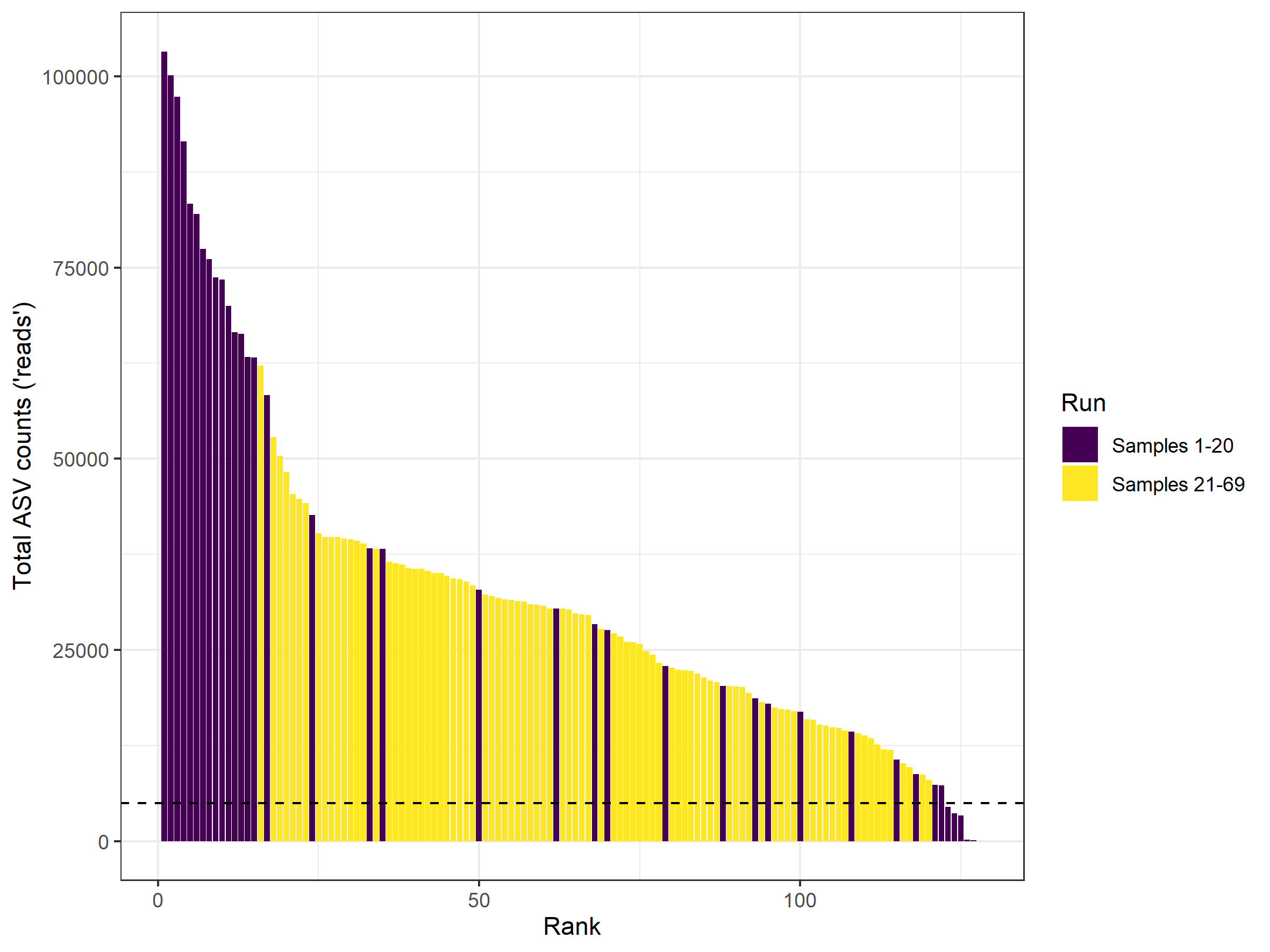


Supplementary figure 1. Overview of amplicon sequence variants (ASVs) obtained from rhizospheric soil samples, after processing the data with DADA2. The sequenced samples are ranked by total ASV counts. The bar colours indicate separate Illumina Miseq runs: purple bars are from run 1 (samples 1-20); yellow bars are from run 2 (samples 21-69). This figure shows all sequenced samples before and after enrichment (128 total); the cut-off for insufficient sequencing depth (< 5000 ASV counts) is indicated by a dashed line.


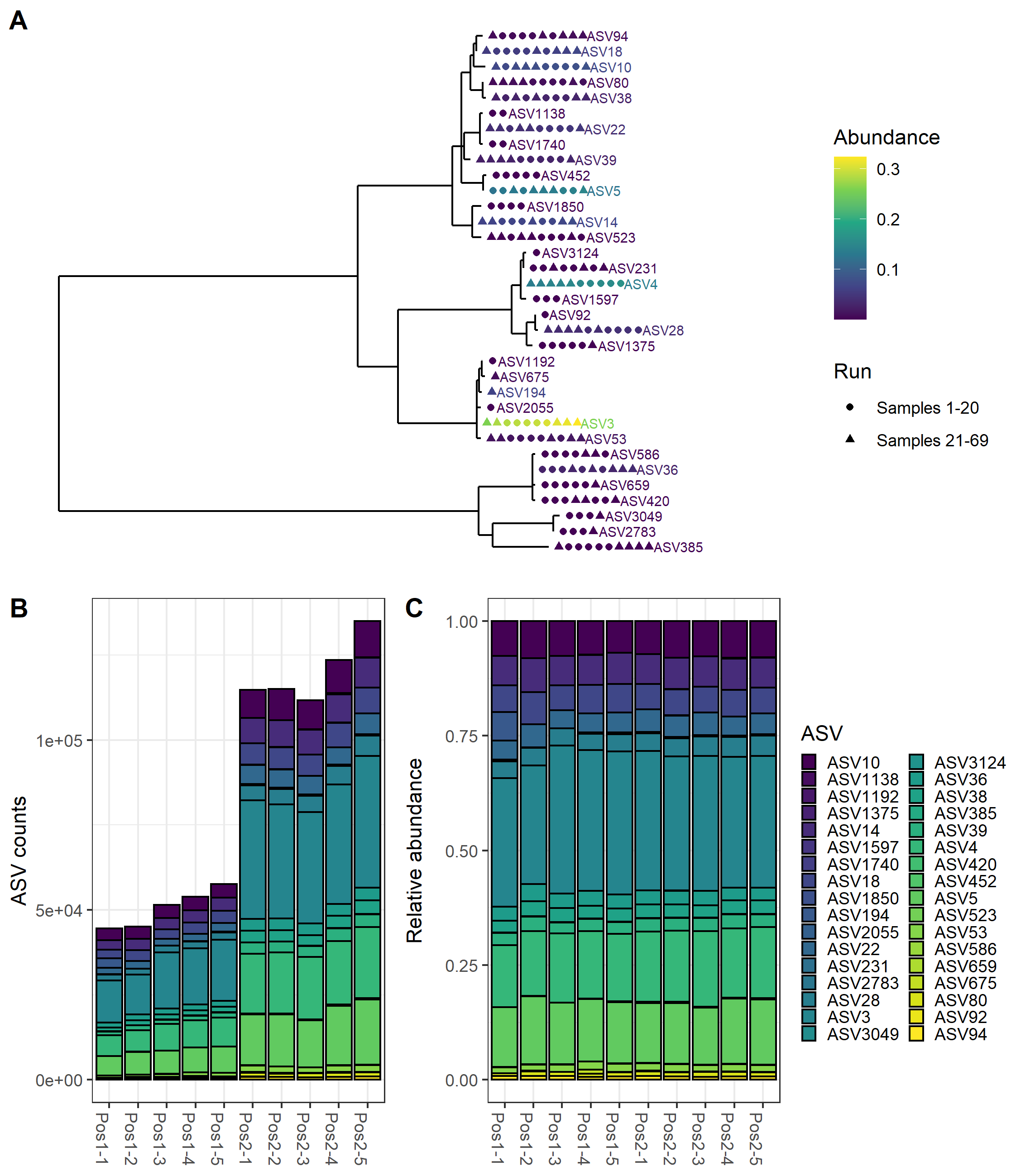


Supplementary figure 2. Phylogeny and ASV (amplicon sequence variant) counts in positive controls used in metabarcoding of rhizospheric soil. In A, Neighbour joining tree of ASVs identified in the positive controls that indicate presence and relative abundance of the ASV per positive control by colour, and the Miseq run the positive control was processed in by shape. The tree, represents a subsection of the Neighbour Joining tree fitted with a GTR+G+I model of all ASVs, rooted in the leaf with the longest path (the root is not in this subsection). In B and C, the absolute and relative abundance of the ASVs in the positive controls are shown respectively. Each bar represents one positive control, each containing the same mixture of DNA from different species with segments of each bar representing one ASV. Pos1-1 to Pos1-5 were run with samples 21-69 and Pos2-1 to 2-5 were run with samples 1-20.


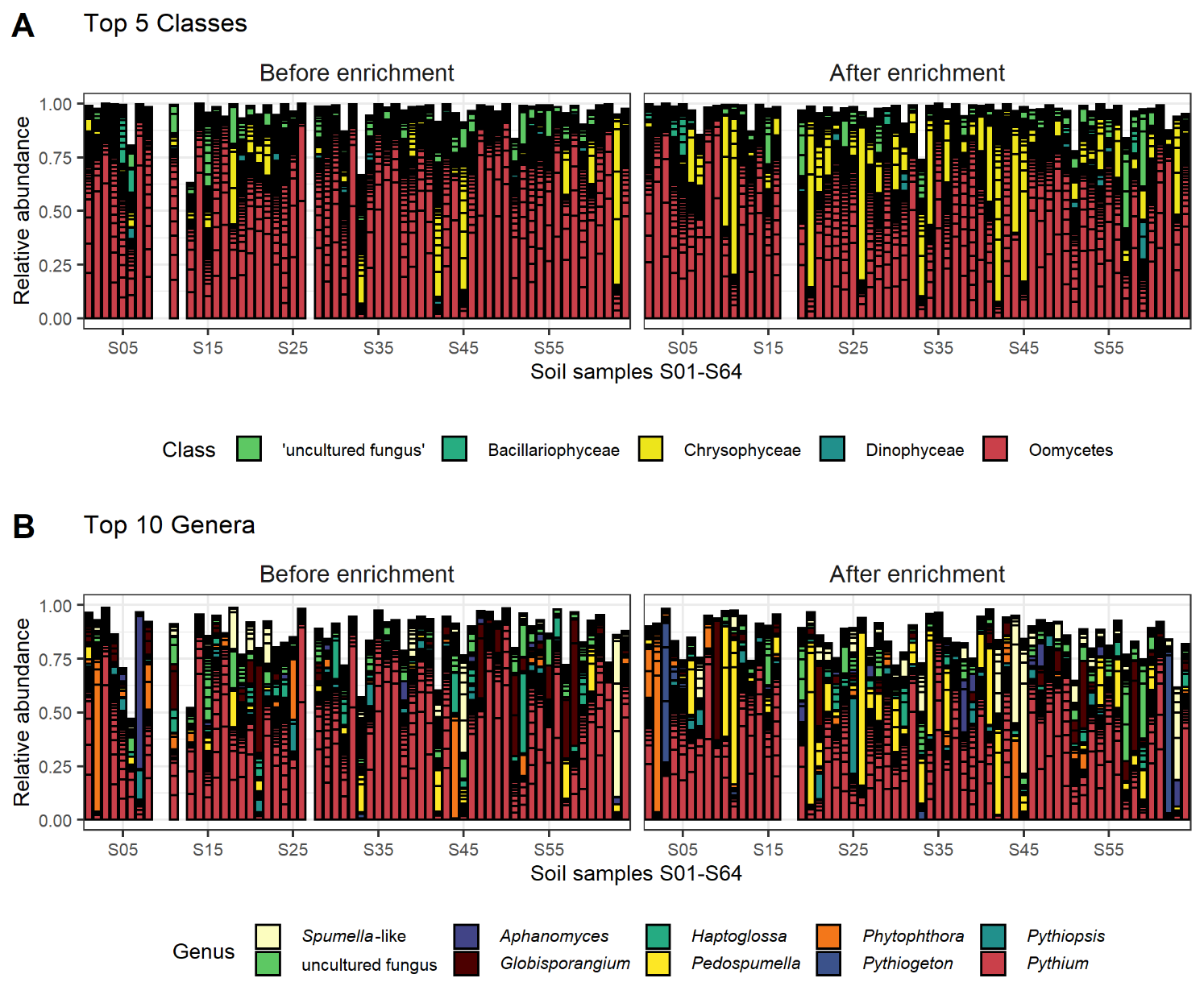


Supplementary figure 3. Overview of the most abundant organisms in rhizospheric soil samples detected by oomycete ITS metabarcoding. Relative abundance of ASVs belonging to the five most abundant taxonomic classes (A) and the 10 most abundant genera (B) before (left) and after enrichment (right). Within a column, one segment represents a single ASV, color coded by taxonomic class/genus. In B, *Globisporangium*, *Pythium* and *Phytophthora* are highlighted in bordeaux, red and orange respectively, while *Aphanomyces* and *Phytiogeton* are colored in shades of purple/dark blue.


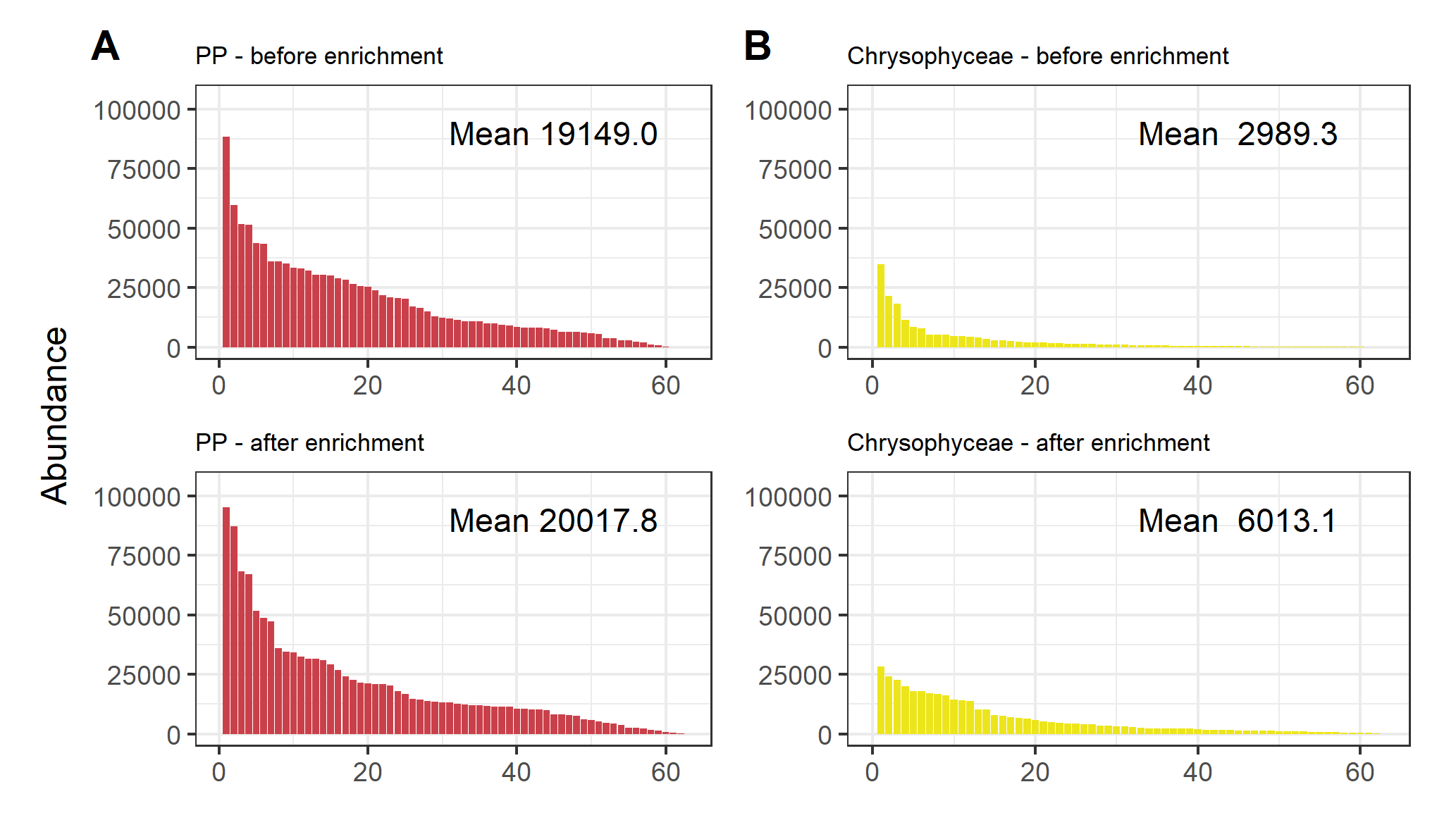


Supplementary figure 4. Absolute abundance of reads belonging to plant-pathogenic (PP) oomycetes according to the FUNGuild classification and Chrysophyceae in samples before and after enrichment. A The absolute abundance of PP per sample in red, ranked from the sample with the highest to the lowest relative abundance of PP before enrichment in the upper graph and after enrichment in the lower graph. B The ranked absolute abundance of Chrysophyceae (yellow) before enrichment (top) and after enrichment (bottom). The mean number of reads belonging to the different taxonomic classes is indicated in each graph.

# Supplementary tables

Supplementary table 1. Overview of all rhizospheric soil samples, whether they yielded DNA and sufficient PCR amplicons for sequencing, the read counts through the processing and associated metadata. The metadata for each numbered soil sample includes the countries from which the samples were imported, which plant species the soil was imported with and processing notes when samples were discarded. Sheet 2 contains information on the species used as positive controls and whether they were found in sequencing.

[**Provided in separate xlsx file**]

**Supplementary table 2.** PCR amplification primers for DNA barcoding and metabarcoding targeting the ITS region.

| Name | Function | Sequence (5’-3’) | Source |
| --- | --- | --- | --- |
| ITS5 | Fwd barcoding | TCCTCCGCTTATTGATATGC | White et al. (1990) |
| ITS4 | Rev barcoding | GGAAGTAAAAGTCGTAACAAGG | White et al. (1990) |
| ITS1-O | Fwd metabarcoding | CGGAAGGATCATTACCAC | Agler et al. (2016) |
| 5.8s-O-Rev | Rev metabarcoding | AGCCTAGACATCCACTGCTG | Agler et al. (2016) |

Supplementary table 3. Occurrence of ASVs in the samples and their full taxonomy. For each ASV, the ASV count is given for all samples. The taxonomy derived from local BLAST against the NCBI nt database is provided on seven taxonomic levels (where available): Kingdom, Phylum, Class, Order, Family, Genus, Species.

[**Provided in separate xlsx file**]

Supplementary table 4. Results of the pairwise t-tests performed for the classes shown in Figure 3. For each class/group of classes, the mean relative abundance before and after enrichment are compared. Full R code used for the analysis can be found in Supplementary Material 3.

| **Genera from** | **group1** | **group2** | **n1** | **n2** | **p** | **p.signif** |
| --- | --- | --- | --- | --- | --- | --- |
| Plant pathogenic oomycetes | Before enrichment | After enrichment | 60 | 62 | 0.03050 | * |
| Chrysophyceae | Before enrichment | After enrichment | 60 | 62 | 0.00818 | ** |
| Other oomycetes | Before enrichment | After enrichment | 60 | 62 | 0.79300 | ns |
| Other classes | Before enrichment | After enrichment | 60 | 62 | 0.98300 | ns |
