# Supplementary material for: DNA Metabarcoding Reveals Broad Presence of Plant Pathogenic Oomycetes in Soil From Internationally Traded Plants
Source: Front Microbiol. 2021 Mar 25;12:637068. doi: 10.3389/fmicb.2021.637068 (PMC8027490; doi:10.3389/fmicb.2021.637068)
Supplement: Supplementary Material 2 — Complete documentation of the R code used for the DADA2 pipeline. The code and outputs are compartmentalized in chunks for separate processes. The raw reads used as input for DADA2 are deposited on www.ebi.ac.uk/ena/data/view/PRJEB40676. Version information for R, RStudio and the libraries used is found at the bottom of the document. The R Markdown file is available on GitLab under gitlab.nibio.no/simeon/oomycete-metabarcoding-supplementary. [file Data_Sheet_2.pdf]

dada2 microbiome pipeline v.4.1

Marie L. Davey, Simeon Rossmann

16.12.2019

This is an R markdown document for the analysis of MiSeq amplicon sequencing data. In order to customise the pipeline, search 'CHANGE ME' to find different settings you should adjust according to your data. Comment SWR: A new chunk was added to allow for quick reanalysis of taxonomy and pooled analysis of multiple samples after error inference. Small changes in chunk 4 (exporting seqtab.RDP in addition) were made to facilitate this. Therefore it's not recommended to 'Run all chunks below' from chunk 4 in this version.

To reproduce the analysis for the manuscript "DNA metabarcoding reveals broad presence of plant pathogenic oomycetes in soil from internationally traded plants", please use the R Markdown version of this document available with all other code on the GitLab repository belonging to the publication

v4 update Comment SWR: Two more chunks were added to integrate LULU post-processing into this pipeline. The first of the two new chunks is a shell script chunk that generates a match list (most similar ASVs for each ASV) using vsearch. The match list can also be generated by other means (f. eks. BLAST). The second of the new chunks is the core LULU chunk that takes the seqtab_nochim file from the dada2 analysis and the match list and performs the LULU post-processing with standard parameters. After this, a new seqtab_nochim, ASV-fasta and RDP taxonomy (optional) are generated in a lulu-subdirectory.

v4.1

- Fixed an error when attempting to plot empty files
- Fixed an error when attempting to sample more files than available for plotting

General comments

This script was used per sequencing run, the runs were then combined using `Poolruns` in the last code chunk. This is recommended since the error profiles that DADA2 estimates may vary from run to run.

Setup R Environment

Comment SWR: If the OS is Linux, this chunk now passes the path and marker variables to the shell script chunk, making it unnecessary to specify this separately below and avoiding issues with misspelled variables. It is possible that this also works with Mac OS, but this was not included since no Mac machine was available for testing. It is generally recommended to run this script under a Linux OS. The raw reads for the manuscript "DNA metabarcoding reveals broad presence of plant pathogenic oomycetes in soil from internationally traded plants" used in this version of the pipeline can be downloaded from The European Nucleotide Archive (ENA).

```r
############set paths and marker name
# CHANGE ME to the directory (ABSOLUTE FILEPATH e.g. on Linux /home/usr/...)
# containing the fastq files after unzipping.
path="/home/simeon/Documents/Bioimmigrants/Run2_all/"
# CHANGE ME to the sequenced marker
marker=c("OITS1")
# CHANGE ME to the path for the taxonomy database, if RDP classifier is used
# (not used in the published version of the manuscript)
tax_database="/CoFiMiTr08_OomycITS1_dada2_DB.fasta"

# set knitr chunk options
knitr::opts_chunk$set(echo = TRUE)
knitr::opts_chunk$set(eval = FALSE)
knitr::opts_chunk$set(tidy = TRUE)
knitr::opts_chunk$set(root.dir=paste0(path))

# load libraries for R session
library(dada2)
library(phyloseq)
library(ggplot2)
library(Biostrings)
library(grid)
library(gridExtra)

# Get info on the operating system. If Linux, make 'path' and 'marker'
# available globally, if not, warn that the script needs additional attention.
os <- Sys.info()['sysname']
if(os=="Linux"){
  Sys.setenv(projectpath=path)
  Sys.setenv(marker=marker)
}else{
  cat(os, "does not support running shell code through R Markdown well.",
  "Please uncomment 'projectpath' and 'marker' in chunk 2 and 'dada2_folder'",
  "in chunk7 manually and adjust them to the appropriate values")
}
```

Setup bash Environment

#basic setup for all markers plus marker specific trimming of the reads Comment SWR: Uncomment and update projectpath and marker if NOT on Linux

```
# Start by creating a file structure for the output and analysis
# This assumes that all of the files from a run are located in a single folder
# basic structure being created is project/marker/raw_data and project/marker/trim

# define a variable with the path to the folder containing the R1/R2 reads
# UNCOMMENT AND CHANGE ME (only if you're not running Linux as your OS) to the
# directory containing the fastq files
  # projectpath="/mnt/c/R/dada2results/Empties_test"

# define a variable with the marker name
# UNCOMMENT AND CHANGE ME (only if you're not running Linux as your OS)
```

```
# to the name of the marker that has been sequenced -
# *NB make sure this EXACTLY matches the 'marker' you defined in the R chunk above
  # marker="OITS"

# make new folders for the marker and the raw data/processed data
 mkdir "${projectpath}"/"${marker}"/
 mkdir "${projectpath}"/"${marker}"/raw_data
 mkdir "${projectpath}"/"${marker}"/trim

 mv "${projectpath}"/*fastq* "${projectpath}"/"${marker}"/raw_data/

#cutadapt will need uncompressed files - use gunzip to do this
gunzip "${projectpath}"/"${marker}"/raw_data/*.fastq.gz

# define variables for the reverse complements of the F and R primers
# CHANGE ME according to the primers you have used. Remove the hashtag (#) from
# the beginning of the two lines that correspond to your marker

# for the Oomycete ITS1 marker:
  rc_Fprimer="GTGGTAATGATCCTTCCG"
  rc_Rprimer="CAGCAGTGGATGTCTAGGCT"

# define a variable for the minimum length parameter to be used in cutadapt
# CHANGE ME according to the length of your primers. It is advisable to use a
# value between 10 and the shorter of the two primer lengths
  MIN_LENGTH=15

## SWR: define a variable for the number of mismatches to be used in cutadapt
# CHANGE ME to the rate of mismatches (0 =< value < 1) you want cutadapt to
# accept when finding the primer. When choosing a value different from 0, it is
# recommended to compare how many sequences get trimmed when using both, 0 and
# the alternative value.
  MISMATCH=0

# define a variable with the path to the cutadapt program
# this command assumes that cutadapt has been added to your PATH variable during
# installation
# CHANGE ME if cutadapt is not in your PATH variable. Comment out the following
# line, and use the line after it.  Change the specified path accordingly

# CUTADAPT="$(which cutadapt) --minimum-length ${MIN_LENGTH} --no-indels \
# -e ${MISMATCH} -f fastq "

CUTADAPT="/usr/local/bin/cutadapt --minimum-length ${MIN_LENGTH} --no-indels \
-e ${MISMATCH} -f fastq "

#use cutadapt to remove the rev primer from the fwd reads and vice versa
for i in $(ls "${projectpath}"/"${marker}"/raw_data/*R1_001.fastq); do python3 \
${CUTADAPT} -a "${rc_Rprimer}"  -o $i.trim.fastq $i > \
"${projectpath}"/"${marker}"/R1_cutadapt_output.txt; done

for i in $(ls "${projectpath}"/"${marker}"/raw_data/*R2_001.fastq); do python3 \
${CUTADAPT} -a "${rc_Fprimer}"  -o $i.trim.fastq $i > \
```

```
"${projectpath}"/"${marker}"/R2_cutadapt_output.txt; done

#compress the files again to save space
gzip "${projectpath}"/"${marker}"/raw_data/*.fastq

#move the trimmed files into a new folder
mv "${projectpath}"/"${marker}"/raw_data/*trim.fastq.gz "${projectpath}"/"${marker}"/trim/
```

#look at a random sample of R1/R2 to assess run quality

```
#Create Lists of Forward and Reverse Filenames and a List of Sample Names
fnFs=sort(list.files(paste0(path,"/",marker,"/trim/"),
                     pattern="_R1_001.fastq.trim.fastq.gz", full.names = TRUE))
fnRs=sort(list.files(paste0(path,"/",marker,"/trim/"),
                     pattern="_R2_001.fastq.trim.fastq.gz", full.names = TRUE))

# SWR: Make a list of fnF and fnR for quality plotting that excludes very small
# files (default is < 100 bytes) which may cause problems in quality plotting
min_file_size <- function(files, minsize=100){
  info <- file.info(files)
  index_bigs <- which(info$size > 100)
  keep <- files[index_bigs]
}

fnFL <- min_file_size(fnFs)
fnRL <- min_file_size(fnRs)

sample_number <- if (length(fnFL) < 10) length(fnFL) else 10
sample_number <- if (length(fnRL) < sample_number) length(fnRL) else sample_number

lapply(c(sample(fnFL, sample_number), sample(fnRL, sample_number)),
       plotQualityProfile)
```

##set parameters for dada2

```
# CHANGE ME according to the quality of the sequencing run. For good quality
# runs, analysing both R1/R2 is desirable. For very poor quality runs, it may be
# worthwhile to analyse only R1. Select from the following: "R01"   "both"
analysis="both"

# CHANGE ME use "TRUE" to assign taxonomy, "FALSE" to proceed without taxonomic
# assignments
assign_taxonomy="FALSE"

# CHANGE ME use "TRUE" to plot quality profiles for each sample, and "FALSE" to
# speed up analysis by skipping this step (plotting takes some time) and "SUB"
# to plot a subset of 10 samples
plotQC="SUB"

# CHANGE ME according to the quality of the sequencing run. This determines the
# maximum expected errors for R1 and R2 during the filtering step. A reasonably
# conservative threshold is (2,2). If the data is of lower quality, it may be
# worthwhile to run with higher EEs ex/ (3,5)
```

```
my_maxEEf=2

# CHANGE ME according to the quality of the sequencing run. This determines the
# maximum expected errors for R1 and R2 during the filtering step. A reasonably
# conservative threshold is (2,2). If the data is of lower quality it may be
# worthwhile to run with higher EEs ex/ (3,5)
my_maxEEr=2

# CHANGE ME according to the quality of the sequencing run. This determines the
# maximum number of ambiguous bases allowed in the reads.
my_maxN=0

# CHANGE ME according to the quality of the sequencing run. This is a PHRED score
# quality treshold - all sequences will be truncated at the first base with a
# quality score below this value
my_truncQ=2

# CHANGE ME according to the quality of the sequencing run and according to the
# length of the target region. This is the length to cut the (forward,reverse)
# sequences at. Use 0,0 for no truncation.
my_truncLen=c(0,0)

# CHANGE ME according to the marker used - this is the minimum length of the
# reads after trimming
my_minLen=135

# CHANGE ME specify the minimum number of bases to overlap during merging,
# must be over 10!
my_minoverlap=30

# CHANGE ME to specify the minimum confidence interval for RDP assignment of
# taxonomy.
my_minBootstrap=80

# SWR: CHANGE ME to TRUE and give a value (number of bases) to collapse ASVs with
# an overlap of 'Collaps_minOverlap' (recommended to be the length of the
# shortest ASV that is identical to a longer one). This is only recommended to
# be used in cases where a number of ASVs occur that differ in length but are
# otherwise identical. If this frequently occurs, it may be an issue of primer
# trimming.
Collapse_overlapping=FALSE
Collapse_minOverlap=200

# SWR: Guide denoising with the help of sequences that are expected to be present.
# Set pseudo_pooling to 'TRUE' and provide a fasta-file of sequences to guide
# with. More info on this https://benjjneb.github.io/dada2/ReleaseNotes_1_8.html
pseudo_pooling = "FALSE"
guide_seqs= "/home/simeon/Documents/..."

parameters=c(paste0("Path: ",path),"","Samples :",
             sapply(strsplit(basename(sort(list.files(
               paste0(path,"/",marker,"/trim/"),
               pattern="_R1_001.fastq.trim.fastq.gz", full.names = TRUE))),
```

```
                "_"), `[`, 1),"",paste0("Analysis type: ",analysis),
              "",paste0("Database type: ",tax_database),"","dada2 Parameters:",
              paste("maximum expected errors R1: ",my_maxEEf),
              paste("maximum expected errors R2:",my_maxEEr),
              paste("maximum ambiguous basses: ",my_maxN),
              paste("minimum length: ",my_minLen),
              paste("truncate at first instance of Qscore: ",my_truncQ),
              paste("minimum overlap during merging: ",my_minoverlap),
              paste("taxonomy bootstrap threshold: ",my_minBootstrap),
              paste("Collapse overlapping ASVs: ", Collapse_overlapping),
              paste("Minimum overlap if collapsing overlapping ASVs: ",
                    Collapse_minOverlap) ,
              paste("Pseudo pooling (DADA with prior sequence info)",
                    pseudo_pooling))

write.table(parameters,paste0(path,"/",marker,"/parameters.txt"),row.names=F)
```

Computational code chunk, no changes needed.

Comment SWR: This chunk now passes the output directory to a global variable on Linux, so that the shell script chunk that generates the match list can access it.

```
#Create Lists of Forward and Reverse Filenames and a List of Sample Names
fnFs=sort(list.files(paste0(path,"/",marker,"/trim/"),
                     pattern="_R1_001.fastq.trim.fastq.gz", full.names = TRUE))
fnRs=sort(list.files(paste0(path,"/",marker,"/trim/"),
                     pattern="_R2_001.fastq.trim.fastq.gz", full.names = TRUE))

# SWR: Make a list of fnF and fnR for quality plotting that excludes very small
# files (default is < 100 bytes) which may cause problems in quality plotting
# and exit the chunk with error
min_file_size <- function(files, minsize=100){
  info <- file.info(files)
  index_bigs <- which(info$size > 100)
  keep <- files[index_bigs]
}

fnFL <- min_file_size(fnFs)
fnRL <- min_file_size(fnRs)

#Extract sample names
sample.names=sapply(strsplit(basename(fnFs), "_"), `[`, 1)

# begin commands for analysing R01/R02 together

if(analysis=="both"){

  #Create paths for the filtered files in a subdirectory called filtered/
  filtFs=file.path(path,marker, "/R01_R02/filtered/",
                   paste0(sample.names, "_F_filt.fastq"))
  filtRs=file.path(path,marker, "/R01_R02/filtered/",
                   paste0(sample.names, "_R_filt.fastq"))
```

```r
outp=paste0(path, "/", marker, "/R01_R02/")

#Filter sequences
##SWR: fixed paired end pairing by adding matchIDs=TRUE
out=filterAndTrim(fnFs, filtFs, fnRs, filtRs,
            maxN=my_maxN, maxEE=c(my_maxEEf,my_maxEEr), truncQ=my_truncQ,
            minLen=my_minLen, truncLen=my_truncLen, rm.phix=TRUE,
            compress=TRUE, multithread=TRUE, matchIDs=TRUE)
head(out)
write.table(out,paste0(path,"/",marker,"/R01_R02/out.txt"))

if(plotQC=="TRUE"){
  # Plot Forward/Reverse Quality scores for each sample
  # (not strictly necessary, time intensive)
  pl=lapply(c(fnFL),plotQualityProfile)
  print(pl)
  ml=marrangeGrob(pl,nrow=2,ncol=1)
  ggsave(paste0(path,"/",marker,"/R01_R02/qualityplots_fwd.pdf"),
         ml,width=20,height=26,unit="cm",dpi=300)
  plr=lapply(c(fnRL),plotQualityProfile)
  print(plr)
  mlr=marrangeGrob(plr,nrow=2,ncol=1)
  ggsave(paste0(path,"/",marker,"/R01_R02/qualityplots_rev.pdf"),
         mlr,width=20,height=26,unit="cm",dpi=300)
}

if(plotQC=="SUB"){
  #Plot Forward/Reverse Quality scores for each sample
  # (not strictly necessary, time intensive)
  sample_number <- if (length(fnFL) < 10) length(fnFL) else 10
  sample_number <- if (length(fnRL) < sample_number) length(fnRL) else sample_number

  pl=lapply(sample(c(fnFL),sample_number),plotQualityProfile)
  print(pl)
  ml=marrangeGrob(pl,nrow=2,ncol=1)
  ggsave(paste0(path,"/",marker,"/R01_R02/qualityplots_fwd.pdf"),
         ml,width=20,height=26,unit="cm",dpi=300)
  plr=lapply(sample(c(fnRL),sample_number),plotQualityProfile)
  print(plr)
  mlr=marrangeGrob(plr,nrow=2,ncol=1)
  ggsave(paste0(path,"/",marker,"/R01_R02/qualityplots_rev.pdf"),
         mlr,width=20,height=26,unit="cm",dpi=300)

}
#dereplicate
derepFs <- derepFastq(filtFs[out[,2]>0], verbose=TRUE)
derepRs <- derepFastq(filtRs[out[,2]>0], verbose=TRUE)

# Name the derep-class objects by the sample names
names(derepFs) <- sample.names[out[,2]>0]
names(derepRs) <- sample.names[out[,2]>0]
```

```r
#Train dada2 to your dataset
errF <- learnErrors(filtFs[out[,2]>0], multithread=TRUE,nbases=1e+09)
errR <- learnErrors(filtRs[out[,2]>0], multithread=TRUE,nbases=1e+09)


## Plot the Estimated Error Rates for the Transition Types
#check that model and data match reasonably well
eFplot=plotErrors(errF, nominalQ=TRUE)
ggsave(paste0(path,"/",marker,"/R01_R02/R1_error_profile.pdf"),
       eFplot,width=20,height=26,unit="cm",dpi=300)
eRplot=plotErrors(errR, nominalQ=TRUE)
ggsave(paste0(path,"/",marker,"/R01_R02/R2_error_profile.pdf"),
       eRplot,width=20,height=26,unit="cm",dpi=300)

#sample inference
if(pseudo_pooling=="TRUE"){
  prior_seqs <- readDNAStringSet(guide_seqs)
  dadaFs <- dada(derepFs, err=errF, priors = prior_seqs, multithread=TRUE)
  dadaRs <- dada(derepRs, err=errR, priors = prior_seqs, multithread=TRUE)
}else{
  dadaFs <- dada(derepFs, err=errF, multithread=TRUE)
  dadaRs <- dada(derepRs, err=errR, multithread=TRUE)
}

#merge forward and reverse reads
mergers <- mergePairs(dadaFs, derepFs, dadaRs, derepRs,
                      minOverlap=my_minoverlap,verbose=TRUE)

# Inspect the merger data.frame from the first sample
head(mergers[[1]])
merge.tab=data.frame(matrix(ncol=10,nrow=0))
colnames(merge.tab)=c("sample","sequence","abundance","forward",
                      "reverse","nmatch","nmismatch","nindel",
                      "prefer","accept")

for(i in (1:length(mergers))){
  if(nrow(mergers[[i]])>0){
    sub=cbind(names(mergers)[i],mergers[[i]])
    merge.tab=rbind(merge.tab,sub)
  } else {}
}
colnames(merge.tab)=c("sample","sequence","abundance","forward",
                      "reverse","nmatch","nmismatch","nindel",
                      "prefer","accept")
write.table(merge.tab,paste0(path,"/",marker,"/R01_R02/mergers.txt"),
            row.names=F)


# make sequence table and distribution table for sequence lengths
seqtab=makeSequenceTable(mergers)
# use only sequences longer than 50bp as the rdp classifier can't accept less
# than that
seqtab=seqtab[,nchar(colnames(seqtab)) >49]
```

```
  ## SWR: If Collapse_overlapping=TRUE, collapses identical sequences together
  # based on an overlap specified in 'Collapse_minOverlap'.
  if(Collapse_overlapping==TRUE){
     seqtab <- collapseNoMismatch(seqtab, minOverlap = Collapse_minOverlap,
                                 orderBy = "abundance", identicalOnly = FALSE,
                                 vec = TRUE, verbose = FALSE)
  }

  dim(seqtab)
  table(nchar(getSequences(seqtab)))

  ## SWR: save RDS format seqtab to allow for merging of multiple runs and later
  # analysis without rerunning the whole pipeline
  saveRDS(seqtab, paste0(path,"/",marker,"/R01_R02/seqtab.rds"))


  #remove chimeric sequences
  seqtab.nochim <- removeBimeraDenovo(seqtab, method="consensus",
                                     multithread=TRUE, verbose=TRUE)
  dim(seqtab.nochim)
  # calculate percent of sequences that are non-chimeric
  sum(seqtab.nochim)/sum(seqtab)
  write.table(seqtab.nochim,paste0(path,"/",marker,"/R01_R02/seqtab.nochim.txt"))

  ## SWR: additional .RDS output table for easy downstream use in e.g. phyloseq
  saveRDS(seqtab.nochim, paste0(path,"/",marker, "/R01_R02/seqtab_nochim.rds"))

  #output table for each sample
  #define a function that counts sequences in a file/object
  getN = function(x) sum(getUniques(x))
  if(min(out[,2])>0){
    track=cbind(out, sapply(dadaFs, getN), sapply(dadaRs, getN),
                sapply(mergers, getN), rowSums(seqtab.nochim),
                rowSums(seqtab.nochim)/out[,1]*100)
    colnames(track) <- c("input", "filtered", "denoisedF", "denoisedR",
                         "merged", "nonchim","percent_retained")
    rownames(track) =sample.names
  } else {
  track=cbind(out[out[,2]>0,], sapply(dadaFs, getN), sapply(dadaRs, getN),
              sapply(mergers, getN), ... = rowSums(seqtab.nochim),
              rowSums(seqtab.nochim)/out[,1][out[,2]>0]*100)
  track=rbind(track,cbind(out[,1][out[,2]==0],out[,2][out[,2]==0],NA,NA,NA,NA,NA))
  colnames(track) <- c("input", "filtered", "denoisedF", "denoisedR",
                       "merged", "nonchim","percent_retained")
  rownames(track) = c(sample.names[out[,2]>0],sample.names[out[,2]==0])
  }

  head(track)
  write.table(track,paste0(path,"/",marker,"/R01_R02/track.txt"))
} else {


  ##########################################################################
```

```r
#  Forward Reads Only
###########################################################################

# Create paths for the filtered files in a subdirectory called filtered/

filtFsR1=file.path(path,marker, "R01_only/filtered", paste0(sample.names,
                                                             "_F_filt.fastq"))

outp=paste0(path, "/", marker, "/R01_only/")

#Filter sequences
outR1=filterAndTrim(fnFs, filtFsR1,
            maxN=my_maxN, maxEE=my_maxEEf, truncQ=my_truncQ,minLen = my_minLen,
            rm.phix=TRUE,
            compress=TRUE, multithread=TRUE)
head(outR1)
write.table(outR1,paste0(path,"/",marker,"/R01_only/out.txt"))

if(plotQC=="TRUE"){
  # Plot Forward/Reverse Quality scores for each sample
  # (not strictly necessary, time intensive)
  pl=lapply(c(fnFL),plotQualityProfile)
  print(pl)
  ml=marrangeGrob(pl,nrow=2,ncol=1)
  ggsave(paste0(path,"/",marker,"/R01_only/qualityplots_fwd.pdf"),ml,
         width=20,height=26,unit="cm",dpi=300)

}

if(plotQC=="SUB"){
  # Plot Forward/Reverse Quality scores for each sample
  # (not strictly necessary, time intensive)
  sample_number <- if (length(fnFL) < 10) length(fnFL) else 10
  pl=lapply(sample(c(fnFL),sample_number),plotQualityProfile)
  print(pl)
  ml=marrangeGrob(pl,nrow=2,ncol=1)
  ggsave(paste0(path,"/",marker,"/R01_only/qualityplots_fwd.pdf"),ml,
         width=20,height=26,unit="cm",dpi=300)

}


#Train dada2 to your dataset
errFR1 <- learnErrors(filtFsR1[outR1[,2]>0], multithread=TRUE)

eFplot=plotErrors(errFR1, nominalQ=TRUE)
ggsave(paste0(path,"/",marker,"/R01_only/R1_error_profile.pdf"),eFplot,
       width=20,height=26,unit="cm",dpi=300)

#dereplicate
derepFsR1 <- derepFastq(filtFsR1[outR1[,2]>0], verbose=TRUE)
# Name the derep-class objects by the sample names
names(derepFsR1) <- sample.names[outR1[,2]>0]
```

```
#sample inference
if(pseudo_pooling=="TRUE"){
  prior_seqs <- readDNAStringSet(guide_seqs)
  dadaFsR1 <- dada(derepFsR1, err=errFR1, priors = prior_seqs,
                   multithread=TRUE)
}else{
  dadaFsR1 <- dada(derepFsR1, err=errFR1, multithread=TRUE)
}


#make sequence table and distribution table for sequence lengths
seqtabR1=makeSequenceTable(dadaFsR1)
##SWR: If Collapse_overlapping=TRUE, collapses identical sequences together
# based on an overlap specified in 'Collapse_minOverlap'.
if(Collapse_overlapping==TRUE){
  seqtabR1 <- collapseNoMismatch(seqtabR1, minOverlap = Collapse_minOverlap,
                                 orderBy = "abundance", identicalOnly = FALSE,
                                 vec = TRUE, verbose = FALSE)
}
dim(seqtabR1)
table(nchar(getSequences(seqtabR1)))
#RDP can't assign taxonomy to sequences shorter than 50bp, so filter to >50bp
seqtabR1=seqtabR1[,nchar(colnames(seqtabR1))>49]

## SWR: save RDS format seqtab to allow for merging of multiple runs and later
# analysis without rerunning the whole pipeline
saveRDS(seqtabR1, paste0(path,"/",marker,"/R01_only/seqtab.rds"))

#remove chimeric sequences
seqtabR1.nochim <- removeBimeraDenovo(seqtabR1, method="consensus",
                                      multithread=TRUE, verbose=TRUE)
dim(seqtabR1.nochim)
# calculate percent of sequences that are non-chimeric
sum(seqtabR1.nochim)/sum(seqtabR1)
write.table(seqtabR1.nochim,paste0(path,"/",marker,
                                   "/R01_only/seqtabR1.nochim"))

##SWR: additional .RDS output table for easy downstream use in e.g. phyloseq
saveRDS(seqtabR1.nochim, paste0(path,"/",marker,
                                "/R01_only/seqtab_nochim.rds"))

#output table for each sample
getN=function(x) sum(getUniques(x))
if(min(outR1[,2])>0){
  trackR1=cbind(outR1, sapply(dadaFsR1,getN),rowSums(seqtabR1.nochim),
                rowSums(seqtabR1.nochim)/outR1[,1]*100)
  colnames(trackR1) <- c("input", "filtered", "denoisedF",
                         "nonchim","percent_retained")
  rownames(trackR1) = c(sample.names)
} else {
  trackR1=cbind(outR1[outR1[,2]>0,], sapply(dadaFsR1, getN),
                rowSums(seqtabR1.nochim),
                rowSums(seqtabR1.nochim)/outR1[,1][outR1[,2]>0]*100)
```

```
    trackR1=rbind(trackR1,cbind(outR1[,1][outR1[,2]==0],
                                outR1[,2][outR1[,2]==0],NA,NA,NA))
    colnames(trackR1) <- c("input", "filtered", "denoisedF",
                           "nonchim","percent_retained")
    rownames(trackR1) = c(sample.names[!outR1[,2]==0],
                          sample.names[outR1[,2]==0])
  }

  head(trackR1)
  write.table(trackR1,paste0(path,"/",marker,"/R01_only/trackR1.txt"))
}

if(analysis=="both"){
if(assign_taxonomy=="TRUE"){
    taxa <- assignTaxonomy(seqtab.nochim,tax_database,multithread=TRUE,
                           minBoot=my_minBootstrap,outputBootstraps = TRUE)
    sum(rownames(taxa[[1]])==colnames(seqtab.nochim))==nrow(taxa[[1]])
    tmp=cbind(rownames(taxa[[1]]),taxa[[1]],taxa[[2]])
    rownames(tmp)=paste0("ASV",1:length(colnames(seqtab.nochim)))
    tmp=data.frame(tmp)
    write.table(tmp,paste0(path,"/",marker,"/R01_R02/taxonomy_ASVs_NC_R1R2.txt"))

    ## SWR: save RDS file omitting bootstrap values for downstream use in e.g
    # phyloseq
    saveRDS(taxa$tax, paste0(path,"/",marker,"/R01_R02/taxa.rds"))

    tmp2=cbind(t(seqtab.nochim),tmp)
    rownames(tmp2)=paste0("ASV",1:length(colnames(seqtab.nochim)))
    write.table(tmp2,paste0(path,"/",marker,"/R01_R02/seqtab.nochim_withtax.txt"))

    # write raw ASVs to a fasta file
    seqsnochim=DNAStringSet(colnames(seqtab.nochim))
    seqsnochim@ranges@NAMES=paste0("ASV",1:length(colnames(seqtab.nochim)))
    writeXStringSet(seqsnochim,paste0(path,"/",marker,"/R01_R02/ASVs_raw.fasta"),
                    format="fasta")

    # write ASVs with taxonmy in the header to a fasta file
    seqsnochim=DNAStringSet(colnames(seqtab.nochim))
    seqsnochim@ranges@NAMES=paste0("ASV",1:length(colnames(seqtab.nochim)),
                                   "|","|",
                                   paste0(tmp$Kingdom,";",tmp$Phylum,";",
                                          tmp$Class,";",tmp$Order,";",
                                          tmp$Family,";",tmp$Genus,";",
                                          tmp$Species))
    writeXStringSet(seqsnochim,paste0(
      path,"/",marker,"/R01_R02/ASVs_withtax.fasta"),format="fasta")
  } else {
    # write raw ASVs to a fasta file
    seqsnochim=DNAStringSet(colnames(seqtab.nochim))
    seqsnochim@ranges@NAMES=paste0("ASV",1:length(colnames(seqtab.nochim)))
    writeXStringSet(seqsnochim,paste0(
      path,"/",marker,"/R01_R02/ASVs_raw.fasta"),format="fasta")
  }
```

```
}else{
  if(assign_taxonomy=="TRUE"){
    taxaR1= assignTaxonomy(seqtabR1.nochim,tax_database,
                           multithread=TRUE,minBoot=my_minBootstrap,
                           outputBootstraps = TRUE)
    sum(rownames(taxaR1[[1]])==colnames(seqtabR1.nochim))==nrow(taxaR1[[1]])
    tmp=cbind(rownames(taxaR1[[1]]),taxaR1[[1]],taxaR1[[2]])
    rownames(tmp)=paste0("ASV",1:length(colnames(seqtabR1.nochim)))
    tmp=data.frame(tmp)
    write.table(tmp,paste0(path,"/",marker,"/R01_only/taxonomy_ASVs_NC_R1.txt"))

    ## SWR: save RDS file omitting bootstrap values for downstream use in e.g
    # phyloseq
    saveRDS(taxaR1$tax, paste0(path,"/",marker,"/R01_only/taxa.rds"))

    tmp2=cbind(t(seqtabR1.nochim),tmp)
    rownames(tmp2)=paste0("ASV",1:length(colnames(seqtabR1.nochim)))
    write.table(tmp2,paste0(
      path,"/",marker,"/R01_only/seqtabR1.nochim_withtax.txt"))

    #write raw ASVs to a fasta file
    seqsnochimR1=DNAStringSet(colnames(seqtabR1.nochim))
    seqsnochimR1@ranges@NAMES=paste0("ASV",1:length(colnames(seqtabR1.nochim)))
    writeXStringSet(seqsnochimR1,paste0(
      path,"/",marker,"/R01_only/ASVs_raw.fasta"),format="fasta")

    #write ASVs with taxonomy in the header to a fasta file
    seqsnochim=DNAStringSet(colnames(seqtabR1.nochim))
    seqsnochim@ranges@NAMES=paste0("ASV",1:length(colnames(seqtabR1.nochim)),"|",
                                   paste0(tmp$Kingdom,";",tmp$Phylum,";",
                                          tmp$Class,";",tmp$Order,";",
                                          tmp$Family,";",tmp$Genus,";",
                                          tmp$Species))
    writeXStringSet(seqsnochim,paste0(
      path,"/",marker,"/R01_only/ASVs_withtax_R1.fasta"),format="fasta")
  }else{
    #write raw ASVs to a fasta file
    seqsnochimR1=DNAStringSet(colnames(seqtabR1.nochim))
    seqsnochimR1@ranges@NAMES=paste0("ASV",1:length(colnames(seqtabR1.nochim)))
    writeXStringSet(seqsnochimR1,paste0(
      path,"/",marker,"/R01_only/ASVs_raw.fasta"),format="fasta")

  }
}

#make paths available to subsequent chunks
dada2_dir <- outp
#If Linux, make 'dada2_folder'available globally.
  if(os=="Linux"){
    Sys.setenv(dada2Folder=dada2_dir)
  }
```

Make match list

Comment SWR: This is a short bash chunk to create the match list necessary for LULU using vsearch (must be installed). If you are on Windows or would rather use BLAST than vsearch check the (LULU Github)[https://github.com/tobiasgf/lulu] for additional info.

```
# UNCOMMENT AND CHANGE ME (only if you're not running Linux as your OS) to the
# directory containing the dada2
dada2Folder="/home/simeon/Documents/Bioimmigrants/OITS1/with_priors_R1_R2"

ASV="${dada2Folder}"/ASVs_raw.fasta
matchList="${dada2Folder}"/match_list.txt

vsearch --usearch_global "${ASV}" --db "${ASV}" --self --id .84 --iddef 1 \
--userout "${matchList}" -userfields query+target+id --maxaccepts 0 \
--query_cov .9 --maxhits 10
```

LULU post-processing

Comment SWR: This chunk will run the LULU post-processing on the seqtab_nochim table and produce a new seqtab_nochim table, as well as a new raw ASV table. It requires the 'seqtab_nochim' and 'match_list' tables as input and will automatically get them when running the whole pipeline on a Linux OS. It also has the posibility to assign taxonomy to this new ASV table using the RDP classifier. All files will be saved to a subdirectory of the main output called 'lulu_output'. In the current form, the original ASV designations from the dada2 pipeline are not retained in the LULU output.

In the published version of the manuscript, LULU was not performed as it removed valid ASVs in the positive control, indicating overly conservative ASV fusion.

```
# If you want to run this as a stand-alone chunk or are on Windows, uncomment
# and change the 'dada2_dir' variable. The directory must contain
# 'match_list.txt' and 'seqtab.nochim.rds'. If you run the whole pipeline on
# Linux, this will be set.
dada2_dir = "/home/simeon/Documents/Bioimmigrants/OITS1/with_priors_R1_R2"

# CHANGE ME to the path for the taxonomy database you will be using for
# identification (if not already specified above)
tax_database=tax_database

# CHANGE ME to specify the minimum confidence interval for RDP assignment of
# taxonomy.
my_minBootstrap=80

# CHANGE ME to "TRUE" or "FALSE" if you want to use settings that differ from
# the setup chunk.
assign_taxonomy=assign_taxonomy

### NO CHANGES NEEDED BELOW
# load libraries (including dada2 so it can be run standalone)
library(lulu)
library(dada2)
library(Biostrings)
```

```r
#Create output directory, read and parse input files
lulu_output = paste0(dada2_dir, "/lulu_output")
dir.create(file.path(lulu_output))
knitr::opts_chunk$set(root.dir=lulu_output)

matchlist <- paste0(dada2_dir, "/match_list.txt")
matchlist <- read.table(matchlist, header=FALSE,
                        as.is=TRUE, stringsAsFactors=FALSE)

ASV_table <- readRDS(paste0(dada2_dir, "/seqtab_nochim.rds"))
ASV_table <- as.data.frame(t(ASV_table))
ASV_list <- paste0("ASV", seq(nrow(ASV_table)))
seq_list <- as.list(row.names(ASV_table))
names(seq_list) <- ASV_list
row.names(ASV_table) <- ASV_list

#Perform lulu post-processing
curated_ASVs <- invisible(lulu(ASV_table, matchlist))

#Parse and save curated seqtab_nochim
seqtab.nochim.lulu <- curated_ASVs$curated_table
seqtab.nochim.lulu$order <- row.names(seqtab.nochim.lulu)
seqtab.nochim.lulu <- seqtab.nochim.lulu[match(ASV_list,
                                               seqtab.nochim.lulu$order),]
seqtab.nochim.lulu <- seqtab.nochim.lulu[complete.cases(seqtab.nochim.lulu),]
seqtab.nochim.lulu$order <- NULL
delASVs <- curated_ASVs$discarded_otus
delASVs <- which(names(seq_list) %in% delASVs)
seq_list <- seq_list[-delASVs]
row.names(seqtab.nochim.lulu) <- seq_list
seqtab.nochim.lulu <- t(seqtab.nochim.lulu)

saveRDS(seqtab.nochim.lulu, file = paste0(lulu_output, "/seqtab_nochim.rds"))

# Optional RDS taxonomy and create seqtab_nochim_withtax and ASV_withtax,
# otherwise just output a new raw ASV table
if(assign_taxonomy=="TRUE"){
  taxa <- assignTaxonomy(seqtab.nochim.lulu,tax_database,multithread=TRUE,
                         minBoot=my_minBootstrap,outputBootstraps = TRUE)
  sum(rownames(taxa[[1]])==colnames(seqtab.nochim.lulu))==nrow(taxa[[1]])
  tmp=cbind(rownames(taxa[[1]]),taxa[[1]],taxa[[2]])
  rownames(tmp)=paste0("ASV",1:length(colnames(seqtab.nochim.lulu)))
  tmp=data.frame(tmp)
  write.table(tmp,paste0(lulu_output,"/taxonomy_ASVs_NC.txt"))
  saveRDS(taxa$tax, paste0(lulu_output, "/taxa.rds"))

  tmp2=cbind(t(seqtab.nochim.lulu),tmp)
  rownames(tmp2)=paste0("ASV",1:length(colnames(seqtab.nochim.lulu)))
  write.table(tmp2,paste0(lulu_output,"/seqtab.nochim_withtax.txt"))

  #write raw ASVs to a fasta file
  seqsnochim=DNAStringSet(colnames(seqtab.nochim.lulu))
  seqsnochim@ranges@NAMES=paste0("ASV",1:length(colnames(seqtab.nochim.lulu)))
```

```
  writeXStringSet(seqsnochim,paste0(lulu_output,"/ASVs_raw.fasta"),
                  format="fasta")

  #write ASVs containing taxonomy in the header to a fasta file
  seqsnochim=DNAStringSet(colnames(seqtab.nochim.lulu))
  seqsnochim@ranges@NAMES=paste0("ASV",1:length(colnames(seqtab.nochim.lulu)),
                                 "|","|",
                                 paste0(tmp$Kingdom,";",tmp$Phylum,";",
                                        tmp$Class,";",tmp$Order,";",tmp$Family,
                                        ";",tmp$Genus,";",tmp$Species))
  writeXStringSet(seqsnochim,paste0(
    lulu_output,"/ASVs_withtax.fasta"),format="fasta")
} else {
  #write raw ASVs to a fasta file
  seqsnochim=DNAStringSet(colnames(seqtab.nochim.lulu))
  seqsnochim@ranges@NAMES=paste0("ASV",1:length(colnames(seqtab.nochim.lulu)))
  writeXStringSet(seqsnochim,paste0(
    lulu_output,"/ASVs_raw.fasta"),format="fasta")
}
```

SWR: Extra chunk to reanalyse

Reanalyse e.g. with a different taxonomy database or pool runs. This chunk can be run completely independently (all libraries load again), given that the seqtab.RDS files were generated before

```
#load libraries for R session
library(dada2)
library(Biostrings)

# reset paths and marker name and reload libraries to make this chunk standalone
# CHANGE ME to the directory containing the file seqtab.rds for run1
path1="/home/simeon/Documents/Bioimmigrants/Run1_rerun_all/OITS1/R01_R02/"
# CHANGE ME to the directory containing the file seqtab.rds for run2 (if applicable)
path2="/home/simeon/Documents/Bioimmigrants/Run2_all/OITS1/R01_R02/"
# CHANGE ME to the directory containing the file seqtab.rds for run3 (if applicable)
path3="C:/Users/mada/Documents/dada2_test/test/"
# CHANGE ME to the directory containing the file seqtab.rds for run4 (if applicable)
path4="C:/Users/mada/Documents/dada2_test/test/"

# CHANGE ME to the path you want to save the output to. If reanalyzing one run,
# output will automatically reset to be equal to 'path1'.
output="/home/simeon/Documents/Bioimmigrants/OITS1/R1_R2_pooled_final/qual2/"

# CHANGE ME  to the path for the taxonomy database you will be using for
# identification (if not already specified above)
tax_database="CoFiMiTr08_OomycITS1_dada2_DB.fasta"

# CHANGE ME to specify the minimum confidence interval for RDP assignment of
# taxonomy.
my_minBootstrap=80

# CHANGE ME "TRUE" to pool multiple runs (add more paths if more than 4 runs are
# to be analysed), "FALSE" to reanalyse a single run
```

```
Poolruns="TRUE"

# CHANGE ME to "TRUE" or "FALSE" if you want to use settings that differ from
# the setup chunk.
assign_taxonomy="FALSE"

if(Poolruns=="TRUE"){
  #could implement for loop here, if ever used widely
  st1 <- readRDS(paste0(path1,"/seqtab.rds"))
  st2 <- readRDS(paste0(path2,"/seqtab.rds"))
  #st3 <- readRDS(paste0(path3,"/seqtab.rds"))
  #st4 <- readRDS(paste0(path4,"/seqtab.rds"))

  #CHANGE ME according to the number of runs to be pooled)
  st.all <- mergeSequenceTables(st1, st2)
}else{
  #If Poolruns=FALSE, only the first path will be reanalysed.
  seqtab.nochim <- readRDS(paste0(path1,"/seqtab_nochim.rds"))
  output <- path1
}

# remove chimeric sequences
if(Poolruns=="TRUE"){
  seqtab.nochim <- removeBimeraDenovo(st.all, method="consensus",
                                       multithread=TRUE, verbose=TRUE)
  dim(seqtab.nochim)
  # calculate percent of sequences that are non-chimeric
  sum(seqtab.nochim)/sum(st.all)
  write.table(seqtab.nochim,paste0(output,"/seqtab.nochim.txt"))
  saveRDS(seqtab.nochim, paste0(output, "/seqtab_nochim.rds"))
}

if(assign_taxonomy=="TRUE"){
  taxa <- assignTaxonomy(seqtab.nochim,tax_database,multithread=TRUE,
                         minBoot=my_minBootstrap,outputBootstraps = TRUE)
  sum(rownames(taxa[[1]])==colnames(seqtab.nochim))==nrow(taxa[[1]])
  tmp=cbind(rownames(taxa[[1]]),taxa[[1]],taxa[[2]])
  rownames(tmp)=paste0("ASV",1:length(colnames(seqtab.nochim)))
  tmp=data.frame(tmp)
  write.table(tmp,paste0(output,"/taxonomy_ASVs_NC.txt"))
  saveRDS(taxa$tax, paste0(output, "/taxa.rds"))

  tmp2=cbind(t(seqtab.nochim),tmp)
  rownames(tmp2)=paste0("ASV",1:length(colnames(seqtab.nochim)))
  write.table(tmp2,paste0(output,"/seqtab.nochim_withtax.txt"))

  #write raw ASVs to a fasta file
  seqsnochim=DNAStringSet(colnames(seqtab.nochim))
  seqsnochim@ranges@NAMES=paste0("ASV",1:length(colnames(seqtab.nochim)))
  writeXStringSet(seqsnochim,paste0(output,"/ASVs_raw.fasta"),format="fasta")

  #write ASVs containing taxonomy in the header to a fasta file
  seqsnochim=DNAStringSet(colnames(seqtab.nochim))
```

```
  seqsnochim@ranges@NAMES=paste0("ASV",1:length(colnames(seqtab.nochim)),"|","|",
                                  paste0(tmp$Kingdom,";",tmp$Phylum,";",
                                         tmp$Class,";",tmp$Order,";",tmp$Family,
                                         ";",tmp$Genus,";",tmp$Species))
  writeXStringSet(seqsnochim,paste0(output,"/ASVs_withtax.fasta"),format="fasta")
} else {
  #write raw ASVs to a fasta file
  seqsnochim=DNAStringSet(colnames(seqtab.nochim))
  seqsnochim@ranges@NAMES=paste0("ASV",1:length(colnames(seqtab.nochim)))
  writeXStringSet(seqsnochim,paste0(output,"/ASVs_raw.fasta"),format="fasta")
}

dada2_dir <- output

#If Linux, make 'dada2_folder'available globally.
  if(os=="Linux"){
    Sys.setenv(dada2Folder=dada2_dir)
  }
```
