# Supplementary material for: DNA Metabarcoding Reveals Broad Presence of Plant Pathogenic Oomycetes in Soil From Internationally Traded Plants
Source: Front Microbiol. 2021 Mar 25;12:637068. doi: 10.3389/fmicb.2021.637068 (PMC8027490; doi:10.3389/fmicb.2021.637068)
Supplement: Supplementary Material 3 — Complete data analysis documentation including a clear description of the input data, the code that was used to analyse it and the various outputs as well as version information for R, RStudio and the libraries used. The code and outputs are compartmentalized in chunks for separate processes. The data files used as input for the Phyloseq section and the R Markdown file are available on GitLab under gitlab.nibio.no/simeon/oomycete-metabarcoding-supplementary. [file Data_Sheet_3.pdf]

Supplementary material 3 - Metabarcoding data processing

Revision 1

Simeon Rossmann

15/02/2021

Introduction

If all needed packages (see below) are installed and the entire Git repository belonging to the publication was cloned so that the file organization is identical, it should be possible to run and reproduce the entire data analysis using this R Markdown file without any changes or adjustments.

This is an R Markdown file containing code to document the data analysis for the manuscript "DNA metabarcoding reveals broad presence of potentially plant pathogenic oomycetes in cross-border transported soil". Results of the DADA2 analysis are parsed into phyloseq and other, generic data objects and metadata is added. In addition to documenting the analysis, the analysis should be fully reproducible using the provided files at the GitLab repository. It is separated into chunks that may be run independently by pressing the *play* button. To reproduce the reported results, all chunks have to be run from top to bottom. **Several additional files** are required in order to run the entire pipeline successfully:

- A sequence table called **'seqtab_nochim.rds'** (automatically generated by the DADA2 pipeline)
- A taxonomy table called **'taxa.rds'** (automatically generated by the Dada2 pipeline)
- A table describing the sample properties called **'descriptors.txt'** (Provided separately)
- Optional (some plots require this): A phylogenic tree file called **'phylotree.rds'** (provided, calculated automatically if not provided)
- Although the pipeline accepts the **'taxa.rds'** taxonomy output from the RDP classifier in DADA2, a **custom_BLAST_tophits_taxonomy** containing the top hits of a BLAST search against the NCBI nt database is provided and used in the manuscript (tab-delimited text)

The packages used in the analysis and their versions are documented at the bottom of the document. These packages can be installed from CRAN or Bioconductor. For the custom **CuPhyR** (Customized phyloseq in R) package that contains functions written by the author, the source GitHub repository is explicitly pointed to and installation should proceed automatically.

Setup

Descriptor table and initialization The file 'descriptors.txt' is a tab-delimited .txt table containing metadata for all samples. It was generated using the chunk below (optional_sample_check = TRUE) and filled using the metadata from Supplementary table 1 but also contains data processing related columns that help adress certain data groups. This chunk points to the path of the input files and loads the necessary libraries. Warnings are disabled for this chunk as at least one function (dir.create) throws an expected warning every time the output path already exists. Set 'warning=TRUE' in the chunk header if you want to output warnings to the knitted document and output space under a given chunk (e.g. if you experience trouble loading certain libraries).

```r
# CHANGE ME according to the location containing 'seqtab_nochim.rds' and other
# relevant files.
path = "Data_files/"

# CHANGE ME to 'TRUE' to list all samples and generate an empty metadata file
optional_sample_check <- "FALSE"

# CHANGE ME to TRUE to update the cuphyr package. This is a package the author
# published to separate out general purpose functions from this script. If the
# package is not installed, it will be installed, regardless of the value chosen
# here.
update_cuphyr <- TRUE

################# NO CHANGES NECESSARY BELOW #################

# global knitr settings to determine the output of the markdown document upon
# 'knitting'
knitr::opts_chunk$set(echo = TRUE)
knitr::opts_chunk$set(root.dir = paste0(path))

# Messages and warnings are turned off globally for a cleaner output.
# Change to TRUE for complete output
knitr::opts_chunk$set(message = FALSE)
knitr::opts_chunk$set(warning = FALSE)
knitr::opts_chunk$set(tidy = FALSE)

# CuPhyR is the author's own package that contains several functions used to
# process phyloseq objects. It is hosted on GitHub and installed/updated using
# this command if update_cuphyr is TRUE
if (update_cuphyr | !"cuphyr" %in% rownames(installed.packages())) {
  devtools::install_github("simeross/cuphyr")
}

# Loading required packages
library(phyloseq)
library(dada2)
library(tidyverse)
library(Biostrings)
library(stringr)
library(phangorn)
library(DECIPHER)
library(ggsci)
library(ggpubr)
library(gridExtra)
library(viridis)
library(treeio)
library(ggtree)
library(ape)
library(cuphyr)
library(data.table)


# Defining output directory, this is where generated plots will be saved to.
```

```r
outp <- paste0(path,"/phyloseq_output")

# Checks whether output path exists and creates it if not. Throws warning if
# directory exists.
dir.create(file.path(outp))

if (optional_sample_check == "TRUE") {
  seqtabcheck <- readRDS(paste0(path,"/seqtab_nochim.rds"))
  samps <- rownames(seqtabcheck)
  lensamps <- length(samps)
  blankcol <- vector(mode = "character", length = lensamps)
  blanktable <- data.frame(SampleIDs = samps,
                           ExampleProperty1 = blankcol,
                           ExampleProperty2 = blankcol,
                           ExampleProperty3 = blankcol)
  write.table(blanktable, file = paste0(path, "/descriptors_blank.txt"),
              sep = "\t", row.names = F)
  cat("'seqtab_nochim.rds' contains samples in the following order:\n", samps,
      "\nThe number of samples in the file is:", lensamps,sep = "\n")
}

# Setting seed for reproducible results of randomized functions (mostly
# phylogenetic trees)
set.seed(20301)

#minmal message to confirm successful chunk pass
cat("Chunk successfully run")
```

```
## Chunk successfully run
```

Parameters This chunk allows the adjustment of several analysis parameters, such as **setting the pruning of control samples** based on keywords, **requiring that a phylogenetic tree be provided or generated**, **defining a minimum ASV count** and **providing an alternative taxonomy**.

```r
## CHANGE ME to "TRUE" to remove control samples from the analysis or "FALSE" to
# analyse all samples.
Prune_Controls <- "TRUE"

## CHANGE ME to a list of unique identifiers that only occur in the names of
# control samples to classify. These samples will be separated into a dedicated
# phyloseq object to be analyzed apart from the core samples. Common examples
# are provided.
controls <- c("Pos", "H2O", "Neg", "Kontr", "Contr", "posK", "NTC", "POSK")

## CHANGE ME to a number of ASV counts [~reads] that analyzed samples should
# minimally have. Samples with lower ASV counts than 'minASVcount' will be
# pruned.
minASVcount <- 5000

## CHANGE ME to "TRUE", if you want to provide a custom taxonomy table instead
# of using the default dada2 output ('taxa.rds').
customTax <- "TRUE"
```

```r
  ## CHANGE ME to the location of the custom taxonomy file. This only matters if
  # customTax="TRUE", otherwise it will be ignored.
  taxfile <- file.path(path, "custom_BLAST_taxonomy_nt.txt")

## CHANGE ME to "TRUE" to generate a phylogenetic tree. This process takes a
# long time depending on the number of sequences (up to days for thousands).
# If a tree is provided as 'phylotree.rds' in 'path', then it will be used
# regardless of the value of 'maketree'
maketree <- "TRUE"

## CHANGE ME to "TRUE" to root the used phylogenetic tree (if one exists) on the
# leaf with the longest branch (outgroup). This makes analyses that rely on the
# phylogenetic tree reproducible instead of picking a random leaf as root when
# calculating UNIFRAC distances. Implementation based on
# http://john-quensen.com/r/unifrac-and-tree-roots/ and answers in
# https://github.com/joey711/phyloseq/issues/597
roottree <- "TRUE"

# Determines whether a new version of the FUNGuild database should be downloaded
# and saved in 'path'. Otherwise uses existing RDS file in 'path'. If none
# exists, a new one will be downloaded regardless of the value of
# 'update_to_current'.
update_to_current <- "FALSE"

#Setting a seed so functions that use randomization produce reproducible results
set.seed(4433)

cat("Chunk successfully run")
```

```
## Chunk successfully run
```

Parsing input data This chunk parses the input data.**This chunk does not require any user inputs**. If no phylogenetic tree with the name 'phylotree.rds' was provided and 'maketree <- "TRUE"', it will be calculated here. The phylogenetic tree is necessary for certain plots that incorporate taxonomic relationships beyond the annotations, such as PCoA (not included here) or tree plots.

```r
### No changes necessary in this chunk

##Read in variables
#Read in Sequence table
seqtabp <- readRDS(paste0(path,"/seqtab_nochim.rds"))
#Read in taxonomy (custom or RDP)
if (customTax == "TRUE") {
  taxap <- read.delim(taxfile, header = TRUE, sep = "\t")
  rownames(taxap) <- colnames(seqtabp)
  taxap <- as.matrix(taxap)
}else{
  taxap <- readRDS(file.path(path,"taxa.rds"))}
#Read in metadata
samp_table <- read.delim(paste0(path, "/descriptors.txt"), header = TRUE,
                         sep = "\t")
#List samples from Sequence table
```

```r
samp_list <- rownames(seqtabp)


# generate phylogenetic tree of ASVs only if there is no file called
# 'phylotree.rds' in the working directory and 'maketree' is "TRUE"
if (!file.exists(paste0(path, "/phylotree.rds"))) {
  if (maketree == "TRUE") {
    ASVs <- getSequences(seqtabp)
    names(ASVs) <- ASVs
    ASV_align <- alignment <- AlignSeqs(DNAStringSet(ASVs), anchor = NA)
    ASV_phang <- phyDat(as(ASV_align, "matrix"), type = "DNA")
    dm <- dist.ml(ASV_phang)
    treeNJ <- NJ(dm)
    fit = pml(treeNJ, data = ASV_phang)
    fitGTR <- update(fit, k = 4, inv = 0.2)
    fitGTR <- optim.pml(fitGTR, model = "GTR", optInv = TRUE, optGamma = TRUE,
                        rearrangement = "stochastic",
                        control = pml.control(trace = 0))
    saveRDS(fitGTR, file = paste0(path, "/phylotree.rds"))}}

## parse into phyloseq object, either with or without phylogenetic tree
# information
row.names(samp_table) <- samp_list
if (file.exists(paste0(path, "/phylotree.rds"))) {
  treep <- readRDS(paste0(path, "/phylotree.rds"))
  p <- phyloseq(otu_table(seqtabp, taxa_are_rows = FALSE),
                sample_data(samp_table), tax_table(taxap),
                phy_tree(treep$tree))
}else{
  p <- phyloseq(otu_table(seqtabp, taxa_are_rows = FALSE),
                sample_data(samp_table), tax_table(taxap))}

##Adding nucleotide info and giving sequences ASV## identifiers
dna <- Biostrings::DNAStringSet(taxa_names(p))
names(dna) <- taxa_names(p)
p <- merge_phyloseq(p, dna)
taxa_names(p) <- paste0("ASV", seq(ntaxa(p)))

##optional pruning
if (Prune_Controls == "TRUE") {
  if (!is.null(controls)) {
    samp_clean <- samp_list[!samp_list %in% grep(paste0(controls,
                                                        collapse = "|"),
                                                 samp_list, value = T)]
    contr_pruned <- setdiff(samp_list, samp_clean)
    ps <- prune_samples(samp_clean, p)
    cat("\n",
        "Number of control samples that were pruned and will not be analysed:\n",
        length(samp_list) - length(samp_clean),
        "\n",
        "The following controls were pruned:\n",
        contr_pruned, "\n", sep = "\n")
  }else{
```

```
    cat("\n\nPrune_Controls is TRUE but 'controls' is empty. No samples were pruned.\n\n")}
}else{ps <- p}
```

```
##
##
## Number of control samples that were pruned and will not be analysed:
##
## 14
##
##
## The following controls were pruned:
##
## H2O-OITS1
## Positiv1-OITS1
## Positiv2-OITS1
## Positiv3-OITS1
## Positiv4-OITS1
## Positiv5-OITS1
## OITS1-H2O2
## OITS1-H2O3
## OITS1-H2O4
## OITS1-Pos1
## OITS1-Pos2
## OITS1-Pos3
## OITS1-Pos4
## OITS1-Pos5
```

```
if (minASVcount > 0) {
  ps.raw <- ps
  samp_pruned <- names(which(sample_sums(ps) < minASVcount))
  ps <- prune_samples(sample_sums(ps) >= minASVcount, ps)
  if (length(samp_pruned) > 0) {
    cat("The following samples were pruned because ASV counts were lower than",
        minASVcount,  ":\n")
    cat(samp_pruned, "\n", sep = "\n")}
  }
```

```
## The following samples were pruned because ASV counts were lower than 5000 :
## 10A-OITS1
## 12A-OITS1
## 17A-OITS1
## 17B-OITS1
## 18B-OITS1
## 19B-OITS1
```

```
#Remove 0 count ASVs (e.g. control ASVs that remain) from the base object
ps <- prune_taxa(taxa_sums(ps) > 0, ps)
ps
```

```
## phyloseq-class experiment-level object
## otu_table()   OTU Table:         [ 5195 taxa and 122 samples ]
## sample_data() Sample Data:       [ 122 samples by 9 sample variables ]
```

```
## tax_table()   Taxonomy Table:    [ 5195 taxa by 7 taxonomic ranks ]
## phy_tree()    Phylogenetic Tree: [ 5195 tips and 5193 internal nodes ]
## refseq()      DNAStringSet:      [ 5195 reference sequences ]
```

```
cat("","Summary of sample counts",
    "Average ASV count per sample (incl. controls and unfiltered):",
    mean(phyloseq::sample_sums(p)),
    "Average ASV count per sample after pruning controls and filtering low count samples:",
    mean(phyloseq::sample_sums(ps)), sep = "\n")
```

```
##
## Summary of sample counts
## Average ASV count per sample (incl. controls and unfiltered):
## 35056.64
## Average ASV count per sample after pruning controls and filtering low count samples:
## 33716.98
```

```
cat("Chunk successfully run")
```

```
## Chunk successfully run
```

Functions This chunk defines various custom functions that are used in chunks below. **This chunk is absolutely necessary for downstream code to work.**

```
################################################

# This funtion defines the leaf with the longest path as the root of the
# phylogenetic tree. This makes results reproducible by avoiding the behaviour
# of some functions that would otherwise pick a random leaf as the root of an
# unrooted phylogenetic tree. Based on answers in
# https://github.com/joey711/phyloseq/issues/597
pick_new_outgroup <- function(tree.unrooted){
      require("magrittr")
      require("data.table")
      require("ape") # for ape::Ntip
      treeDT <-
          cbind(
              data.table(tree.unrooted$edge),
              data.table(length = tree.unrooted$edge.length)
          )[1:Ntip(tree.unrooted)] %>%
          cbind(data.table(id = tree.unrooted$tip.label))
      # longest terminal branch as outgroup
      new.outgroup <- treeDT[which.max(length)]$id
      return(new.outgroup) }

################################################

# Generic save function for plots that checks whether file exists and if so,
# creates a new one with d/m/y+time info to avoid overwriting.
save_plot <- function(pl, filetype = ".tiff", plot_name = "my_plot"){
  name <- paste0("/",plot_name,filetype)
  if (file.exists(paste0(outp, name))) {
```

```
    name <- paste0("/", plot_name, "_", format(Sys.time(),
                                              "%d-%m-%y_%H%M%S"),filetype)}
  ggsave(paste0(outp, name), pl, width = wp, height = hp,
         unit = "cm", dpi = 300)
}

#################################################

# Function to download and parse the FUNGuild, adapted from S. Faye Smith (2018)
# in https://rpubs.com/faysmith/metabarcoding
parse_funguild <- function(url = 'http://www.stbates.org/funguild_db.php',
                           tax_name = TRUE){
   require(XML)
   require(jsonlite)
   require(RCurl)
  ## Parse data
  tmp <- XML::htmlParse(url)
  tmp <- XML::xpathSApply(doc = tmp, path = "//body", fun = XML::xmlValue)
  ## Read url and convert to data.frame
  db <- jsonlite::fromJSON(txt = tmp)
  ## Remove IDs
  db$`_id` <- NULL
  if (tax_name == TRUE) {
    ## Code legend
    ## Taxon Level: A numeral corresponding the correct taxonomic level for the
    # taxon
    taxons <- c(
      "keyword",                                                      # 0
      "Phylum", "Subphylum", "Class", "Subclass", "Order", "Suborder", # 3:8
      "Family", "Subfamily", "Tribe", "Subtribe", "Genus",             # 9:13
      "Subgenus", "Section", "Subsection", "Series", "Subseries",      # 15:19
      "Species", "Subspecies", "Variety", "Subvariety", "Form",        # 20:24
      "Subform", "Form Species")
    ## Table with coding
    taxmatch <- data.frame(
      TaxID = c(0, 3:13, 15:26),
      Taxon = factor(taxons, levels = taxons))
    ## Match taxon codes
    db$taxonomicLevel <- taxmatch[match(x = db$taxonomicLevel,
                                        table = taxmatch$TaxID), "Taxon"]
  }
  ## Add database dump date as attributes to the result
  attr(db, "DownloadDate") <- date()
  db <- as_tibble(db)
  return(db)
}


cat("Chunk successfully run")
```

```
## Chunk successfully run
```

This chunk calculates most phyloseq objects, vectors, lists and tables that are used to generate figures and

other output below. This somewhat clutters the global environment but keeps the data available and makes the chunks that generate output more efficient. **This chunk is absolutely necessary for downstream code to work.**

```
# Get a tbl of the base objects for easier access in some phyloseq-independent
# analyses
ps_tbl <- as_tibble(psmelt(ps))

samp_sums <- ps_tbl %>%
            dplyr::group_by(SampleIDs) %>%
            summarise(sum_Abundance = sum(Abundance))

ps_tr_tbl <- left_join(ps_tbl, samp_sums, by = "SampleIDs") %>%
  dplyr::mutate(Rel_Abundance = Abundance/sum_Abundance) %>%
  dplyr::mutate(Prcnt_Abundance = Rel_Abundance*100)

# Transformed
ps.trans <- transform_sample_counts(ps, function(ASV) ASV/sum(ASV))

# Just controls
ps.contr <- subset_samples(p, Comment == "Control")
ps.contr <- prune_taxa(taxa_sums(ps.contr) >= 20, ps.contr)
ps.transcont <- transform_sample_counts(ps.contr, function(ASV) ASV/sum(ASV))

# FUNGuild update check and/or load
if (update_to_current == "TRUE" | !file.exists(paste0(path,
                                                      "/FUNGuild-db.rds"))) {
  FUNGuild <- parse_funguild()
  saveRDS(FUNGuild, file = paste0(path, "/FUNGuild-db.rds"))
}else{
  FUNGuild <- readRDS(paste0(path, "/FUNGuild-db.rds"))
}
FUNGuild_tbl <- select(FUNGuild, taxon, trophicMode:growthForm)

# Generate a ps_tbl subset for oomycetes
ps_tbl_Oomycs <- filter(ps_tbl, str_detect(Class, 'Oomycetes')) %>%
                select(OTU, Abundance, Kingdom:Species) %>%
                mutate(Species = gsub("_", " ", Species)) %>%
                group_by(OTU)
# Make a tbl of all oomycete ASVs and their classificaiton according to FUNGuild
ASVs_funguild <- summarise(ps_tbl_Oomycs, Abundance_sum = sum(Abundance)) %>%
                left_join(ps_tbl_Oomycs, by = "OTU") %>%
                select(-Abundance) %>%
                distinct() %>%
                left_join(FUNGuild_tbl, by = c("Genus" = "taxon")) %>%
                left_join(FUNGuild_tbl, by = c("Species" = "taxon")) %>%
                dplyr::rename(trophic_genus = trophicMode.x,
                              guild_genus = guild.x,
                              trophic_species = trophicMode.y,
                              guild_species = guild.y) %>%
                arrange(Genus) %>%
                unite(Plant_pathogenic, c(guild_genus, guild_species),
                      remove = FALSE) %>%
                mutate(Plant_pathogenic = str_replace(
```

```
                  Plant_pathogenic, "Plant Pathogen_Plant Pathogen", "++")) %>%
                mutate(Plant_pathogenic = str_replace(
                  Plant_pathogenic, "Plant Pathogen_NA", "+")) %>%
                mutate(Plant_pathogenic = str_replace(
                  Plant_pathogenic, "NA_NA", "-")) #%>%

# Guild status of all oomyc genera and subets of pp genera and other genera
genus_list <- ASVs_funguild %>%
              dplyr::group_by(Genus, guild_genus) %>%
              summarise()

genera_pp <- filter(genus_list, guild_genus == "Plant Pathogen") %>%
              select(Genus) %>%
              unlist() %>%
              unname()

genera_other <- filter(genus_list,
                       guild_genus != "Plant Pathogen" | is.na(guild_genus)) %>%
              select(Genus) %>%
              unlist() %>%
              unname()

# Subset by vector of taxonomy
## CHANGE me to a vector containing taxonomic terms to subset on
subVector <- c("Oomycetes")
## CHANGE ME to the taxonomic level at which you want to search for an entry
subTax <- "Class"

subASVs <- cuphyr::list_subset_ASVs(subv = subVector, taxlvlsub = subTax)
ps.subs <- prune_taxa(subASVs, ps)
ps.transsub <- prune_taxa(subASVs, ps.trans)

# Subset by sample descriptor
ps.bait <- subset_samples(ps, Bait == "Enriched")
ps.untr <- subset_samples(ps, Bait == "Before enrichment")
samps.bait <- sample_names(ps.bait)
samps.untr <- sample_names(ps.untr)
ps.bait <- prune_samples(samps.bait, ps)
ps.untr <- prune_samples(samps.untr, ps)
ps.bait.trans <- prune_samples(samps.bait, ps.trans)
ps.untr.trans <- prune_samples(samps.untr, ps.trans)

# Create subset lists
subASVsAlg <- cuphyr::list_subset_ASVs(subv = c("Chrysophyceae"),
                                        taxlvlsub = "Class")
subASVsOom <- cuphyr::list_subset_ASVs(subv = c("Oomycetes"),
                                        taxlvlsub = "Class")
subASVsPP <- cuphyr::list_subset_ASVs(subv = c(genera_pp),
                                       taxlvlsub = "Genus")
subASVs_uncultured <- cuphyr::list_subset_ASVs(subv = c("uncultured"),
                                                taxlvlsub = "Genus")
# remove 'uncultured' entries from the PP ASVs, since these should not be
# counted as PP.
```

```r
subASVsPP <- setdiff(subASVsPP, subASVs_uncultured)

# Taxonomic subset on sample descriptor subset
ps.b.tr.Alg <- prune_taxa(subASVsAlg, ps.bait.trans)
ps.u.tr.Alg <- prune_taxa(subASVsAlg, ps.untr.trans)
ps.b.Alg <- prune_taxa(subASVsAlg, ps.bait)
ps.u.Alg <- prune_taxa(subASVsAlg, ps.untr)
ps.b.tr.Oom <- prune_taxa(subASVsOom, ps.bait.trans)
ps.u.tr.Oom <- prune_taxa(subASVsOom, ps.untr.trans)
ps.b.tr.PP <- prune_taxa(subASVsPP, ps.bait.trans)
ps.u.tr.PP <- prune_taxa(subASVsPP, ps.untr.trans)
ps.b.PP <- prune_taxa(subASVsPP, ps.bait)
ps.u.PP <- prune_taxa(subASVsPP, ps.untr)

# Rank some ps objects
ranksBaitAlgae <- cuphyr::make_ranked_sums(ps.b.tr.Alg, myset = "Chrysophyceae")
ranksUntrAlgae <- cuphyr::make_ranked_sums(ps.u.tr.Alg, myset = "Chrysophyceae")
ranksBaitOom <- cuphyr::make_ranked_sums(ps.b.tr.Oom, myset = "All oomycetes")
ranksUntrOom <- cuphyr::make_ranked_sums(ps.u.tr.Oom, myset = "All oomycetes")
ranksBaitPP <- cuphyr::make_ranked_sums(ps.b.tr.PP, myset = "PP")
ranksUntrPP <- cuphyr::make_ranked_sums(ps.u.tr.PP, myset = "PP")

ranksBaitAlgae_tot <- cuphyr::make_ranked_sums(ps.b.Alg,
                                               myset = "Chrysophyceae_tot")
ranksUntrAlgae_tot <- cuphyr::make_ranked_sums(ps.u.Alg,
                                               myset = "Chrysophyceae_tot")
ranksBaitPP_tot <- cuphyr::make_ranked_sums(ps.b.PP, myset = "PP_tot")
ranksUntrPP_tot <- cuphyr::make_ranked_sums(ps.u.PP, myset = "PP_tot")

### rerank tables for combined figures
OomRankPP <- ranksBaitOom
# Adopt rank number from PP so that the sample order is the same
OomRankPP$Rank <- ranksBaitPP$Rank
OomRankPP$Set <- "Other oomycetes"
# Analogous to above
UntrOomRankPP <- ranksUntrOom
UntrOomRankPP$Rank <- ranksUntrPP$Rank
UntrOomRankPP$Set <- "Other oomycetes"
AlgaeInvRankOom <- ranksBaitAlgae
AlgaeInvRankOom$Rank <- ranksBaitPP$Rank
# Add ASV counts so that Chrysophyceae appear above the total Oomycete bars in
# the overlay
AlgaeInvRankOom$Abundance <- AlgaeInvRankOom$Abundance + OomRankPP$Abundance
UntrAlgaeInvRankOom <- ranksUntrAlgae
UntrAlgaeInvRankOom$Rank <- ranksUntrPP$Rank
UntrAlgaeInvRankOom$Abundance <- UntrAlgaeInvRankOom$Abundance +
  UntrOomRankPP$Abundance
# Oom without PP
ranksBaitOwoP <- OomRankPP
ranksBaitOwoP$Set <- "Other oomycetes"
ranksBaitOwoP$Abundance <- ranksBaitOwoP$Abundance - ranksBaitPP$Abundance
ranksBaitOwoP <- ranksBaitOwoP[order(-ranksBaitOwoP$Abundance),]
ranksBaitOwoP$Rank <- c(1:nrow(ranksBaitOwoP))
```

```
ranksUntrOwoP <- UntrOomRankPP
ranksUntrOwoP$Set <- "Other oomycetes"
ranksUntrOwoP$Abundance <- ranksUntrOwoP$Abundance - ranksUntrPP$Abundance
ranksUntrOwoP <- ranksUntrOwoP[order(-ranksUntrOwoP$Abundance),]
ranksUntrOwoP$Rank <- c(1:nrow(ranksUntrOwoP))
# Make dummy tables for 'other' ASV counts that fills up the sample bars to 1
# and contains the total ASV count for ASVs other than Oomycetes and
# Chrysophyceae in the column 'OtherASVcounts'
OtherASVcounts <- 1 - AlgaeInvRankOom$Abundance
OthersRankPP <- cbind(AlgaeInvRankOom, OtherASVcounts)
OthersRankPP$Abundance <- 1
OthersRankPP$Set <- "Other classes"
# Rank Other ASVs for untreated and enriched samples to display in B and C
# respectively
rankedOthersBait <- OthersRankPP[order(-OthersRankPP$OtherASVcounts),]
rankedOthersBait$Rank <- c(1:nrow(rankedOthersBait))
# Analogous to above
OtherASVcounts <- 1 - UntrAlgaeInvRankOom$Abundance
UntrOthersRankPP <- cbind(UntrAlgaeInvRankOom, OtherASVcounts)
UntrOthersRankPP$Abundance <- 1
UntrOthersRankPP$Set <- "Other classes"
rankedOthersUntr <- UntrOthersRankPP[order(-UntrOthersRankPP$OtherASVcounts),]
rankedOthersUntr$Rank <- c(1:nrow(rankedOthersUntr))

if (roottree == "TRUE") {
  my.tree <- phy_tree(ps)
  out.group <- pick_new_outgroup(my.tree)
  new.tree <- ape::root(my.tree, outgroup = out.group, resolve.root = TRUE)
  phy_tree(ps) <- new.tree}

# export ps in clean table format for Supplementary Material
ps_tbl_wide <- ps_tbl %>%
               arrange(Soil, Bait) %>%
               pivot_wider(id_cols = c(OTU, Kingdom, Phylum,
                                       Class, Order, Family,
                                       Genus, Species),
                           names_from = c(Soil, Bait), values_from = Abundance)
ps_tbl_export <- ps_tbl_wide %>%
                 mutate(num_ASV = as.numeric(str_remove(OTU, "ASV"))) %>%
                 arrange(num_ASV) %>% select(-num_ASV) %>%
                 dplyr::rename(ASV = OTU)
# export as csv
write_csv(ps_tbl_export, path = file.path(path, "Supplementary_table_3.csv"))

# Optional: export as xlsx, requires 'writexl' package
#writexl::write_xlsx(ps_tbl_export, path = file.path(path,
#                                                  "Supplementary table 3.xlsx"))

cat("Chunk successfully run")
```

```
## Chunk successfully run
```

Output

The chunks below will produce various plots and other output. Each chunk is headed by a description of the output and may contain some parameters to adjust the output.

Plot looks This chunk sets the background structure and color palette. Viridis was chosen because it is optimized for grey-scale printing and various types of color blindness and More info on the Viridis palette can be found on the Viridis info page.

```
## CHANGE ME to preferred ggplot2 theme. Recommended: "theme_bw()".
theme_set(theme_bw())

my_scale_col <- scale_color_viridis(discrete = TRUE)
my_scale_fill <- scale_fill_viridis(discrete = TRUE)

# Custom, more narrow color ranges based on viridis
# Base order to have adjacent colors be distinct from each other
sort_colors <- c(rbind(c(1:5), c(6:10), c(11:15), c(16:20)))

# Customized color vectors
n_col <- 20
viridis_greens <- viridis(n_col,  option = "D",
                          begin = 0.85 ,end = 0.7)[sort_colors]
viridis_reds <- viridis(n_col,  option = "B",
                        begin = 0.7 ,end = 0.5)[sort_colors]
viridis_blues <- viridis(n_col,  option = "D",
                         begin = 0.2 ,end = 0.4)[sort_colors]
viridis_yellows <- viridis(n_col,  option = "D",
                           begin = 1 ,end = 0.9)[sort_colors]
viridis_dark <- viridis(n_col,  option = "A",
                        begin = 0 ,end = 0.1)[sort_colors]
viridis_light <- viridis(n_col,  option = "A",
                         begin = 1 ,end = 0.9)[sort_colors]
sub_viridis <- list(viridis_greens, viridis_blues, viridis_yellows,
                    viridis_light, viridis_reds, viridis_dark)

cat("Chunk successfully run")
```

```
## Chunk successfully run
```

Positive controls This chunk generates an overview over the positive controls (Supplementary Fig. 2)

```
## CHANGE ME to the secondary parameter of interest (categories on the x-axis).
# Accepted values are the column headers in your descriptor file.
comboPar <- "Soil"

# CHANGE ME to the primary parameter of interest (separate panels).
# Accepted values are the column headers in your descriptor file.
treePar <- "Run"

## CHANGE ME to the taxonomic level of interest (color coding).
# Accepted values are the column headers in your descriptor file.
```

```r
taxlvltre <- "OTU"
taxlvlbar <- "OTU"

##CHANGE ME to change the width (in cm) of the output.
wp <- 18
#CHANGE ME to change the height (in cm) of the output.
hp <- 20.8
#CHANGE ME to change the resolution (in dpi) of the output.
res <- 150

topnpplot <- plot_bar(ps.contr, x = comboPar, fill = taxlvlbar) +
  scale_fill_viridis_d() +
  theme(axis.title.x = element_blank(), legend.position = "none",
        legend.key.size = unit(3, "mm")) + ylab("ASV counts") +
  guides(col = guide_legend(ncol = 3)) + labs(fill = "ASV")

topntplot <- plot_bar(ps.transcont, x = comboPar, fill = taxlvlbar) +
  scale_fill_viridis_d() +
  theme(axis.title.x = element_blank(),
        legend.position = "none", legend.key.size = unit(3, "mm")) +
  ylab("Relative abundance") +
  guides(col = guide_legend(ncol = 3)) +
  labs(fill = "ASV")

tre <- plot_tree(ps.transcont, ladderize = "left",
                 label.tips = taxlvltre, color = "abundance",
                 text.size = 2.5, shape = "Run") +
  scale_color_viridis_c(aesthetics = c("color","fill")) +
  theme(legend.position = "left", panel.border = element_blank())

combocontr <- ggarrange(tre, ggarrange(topnpplot, topntplot,
                                       ncol = 2, labels = c("B", "C"),
                                       align = "hv", common.legend = TRUE,
                                       legend = "right"), nrow = 2,
                        legend = "right", labels = c("A"))

save_plot(combocontr, plot_name = "Barplot_controls_combined_supfig2")

combocontr
```

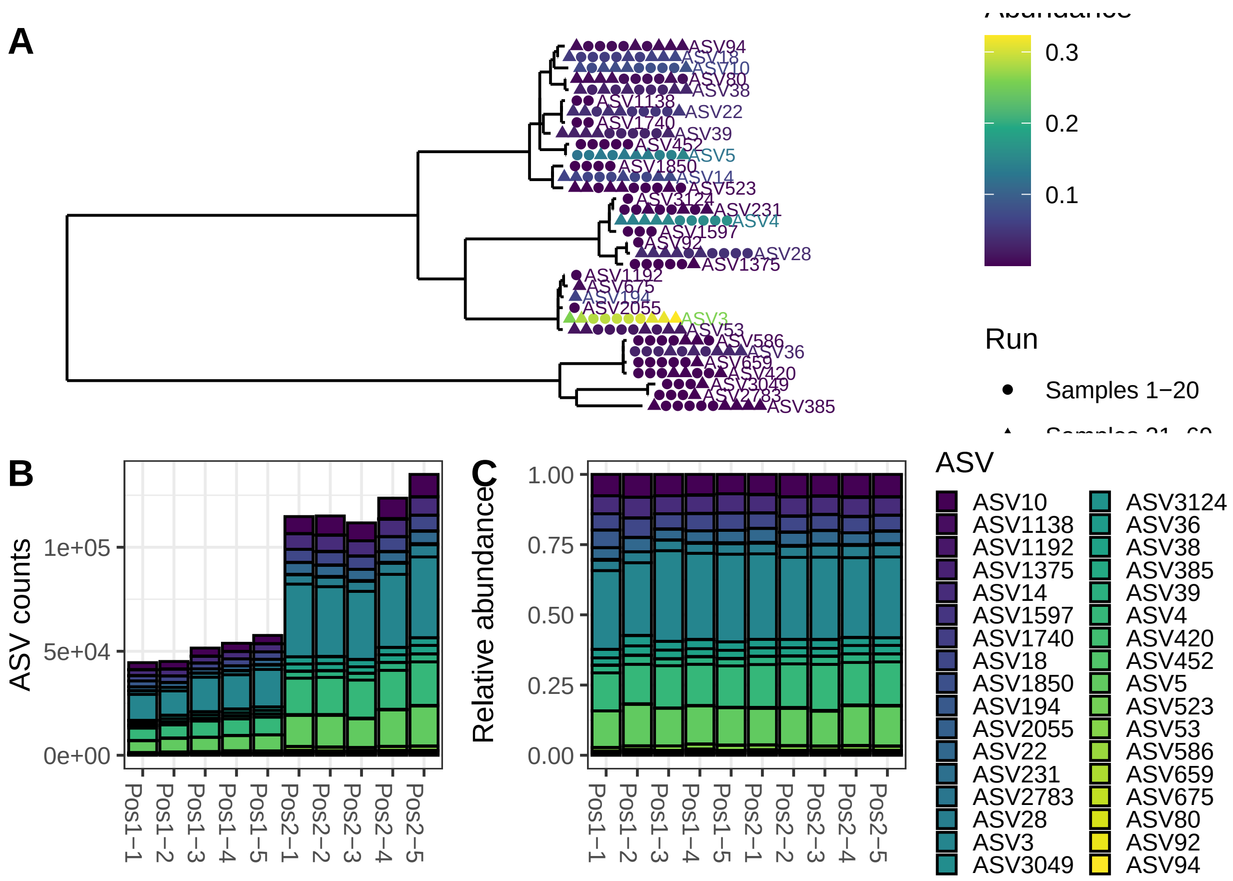


```r
cat("Chunk successfully run")
```

```
## Chunk successfully run
```

Phylogenetic tree of positive controls This chunk generate a phylogenetic tree of the positive control sequences and ASVs detected in the positive controls.

```r
## CHANGE ME to change the width (in cm) of the output.
wp <- 18
# CHANGE ME to change the height (in cm) of the output.
hp <- 13.5

real_contr <- refseq(ps.contr)
seqs <- readDNAStringSet(file = "Data_files/Pos_contrseqs.fasta",
                         format = "fasta")
seqs <- DNAStringSet(c(real_contr, seqs))
align <- AlignSeqs(DNAStringSet(seqs), anchor = NA)
```

```
## Determining distance matrix based on shared 9-mers:
## ================================================================================
##
## Time difference of 0.02 secs
##
## Clustering into groups by similarity:
```

```
## ================================================================================
##
## Time difference of 0.03 secs
##
## Aligning Sequences:
## ================================================================================
##
## Time difference of 1.15 secs
##
## Iteration 1 of 2:
##
## Determining distance matrix based on alignment:
## ================================================================================
##
## Time difference of 0.01 secs
##
## Reclustering into groups by similarity:
## ================================================================================
##
## Time difference of 0.02 secs
##
## Realigning Sequences:
## ================================================================================
##
## Time difference of 0.52 secs
##
## Iteration 2 of 2:
##
## Determining distance matrix based on alignment:
## ================================================================================
##
## Time difference of 0.01 secs
##
## Reclustering into groups by similarity:
## ================================================================================
##
## Time difference of 0.02 secs
##
## Realigning Sequences:
## ================================================================================
##
## Time difference of 0.03 secs
##
## Refining the alignment:
## ================================================================================
##
## Time difference of 0.19 secs
```

```
seqs_phang <- phangorn::phyDat(as(align, "matrix"), type = "DNA")
seqs_dm <- phangorn::dist.ml(seqs_phang)
seqs_treeNJ <- NJ(seqs_dm)
seqs_fit = pml(seqs_treeNJ, data = seqs_phang)
fitGTRseqs <- update(seqs_fit, k = 4, inv = 0.2)
fitGTRseqs <- optim.pml(fitGTRseqs, model = "GTR", optInv = TRUE,
```

```r
                           optGamma = TRUE,
                          rearrangement = "stochastic",
                          control = pml.control(trace = 0))

out.group <- pick_new_outgroup(fitGTRseqs$tree)
new.tree <- ape::root(fitGTRseqs$tree, outgroup = out.group,
                      resolve.root = TRUE)
fitGTRseqs_outgroup <- new.tree

# GTR_tree <- treeio::as.treedata(fitGTRseqs, type="ml")
kontr_ASV_tree <- ggtree(fitGTRseqs_outgroup) +
  geom_tree() + geom_treescale(x = 1) + geom_tiplab() + xlim(0,4)

save_plot(kontr_ASV_tree, plot_name = "Pos-ASVs_kontr-seqs_tree")
kontr_ASV_tree
```

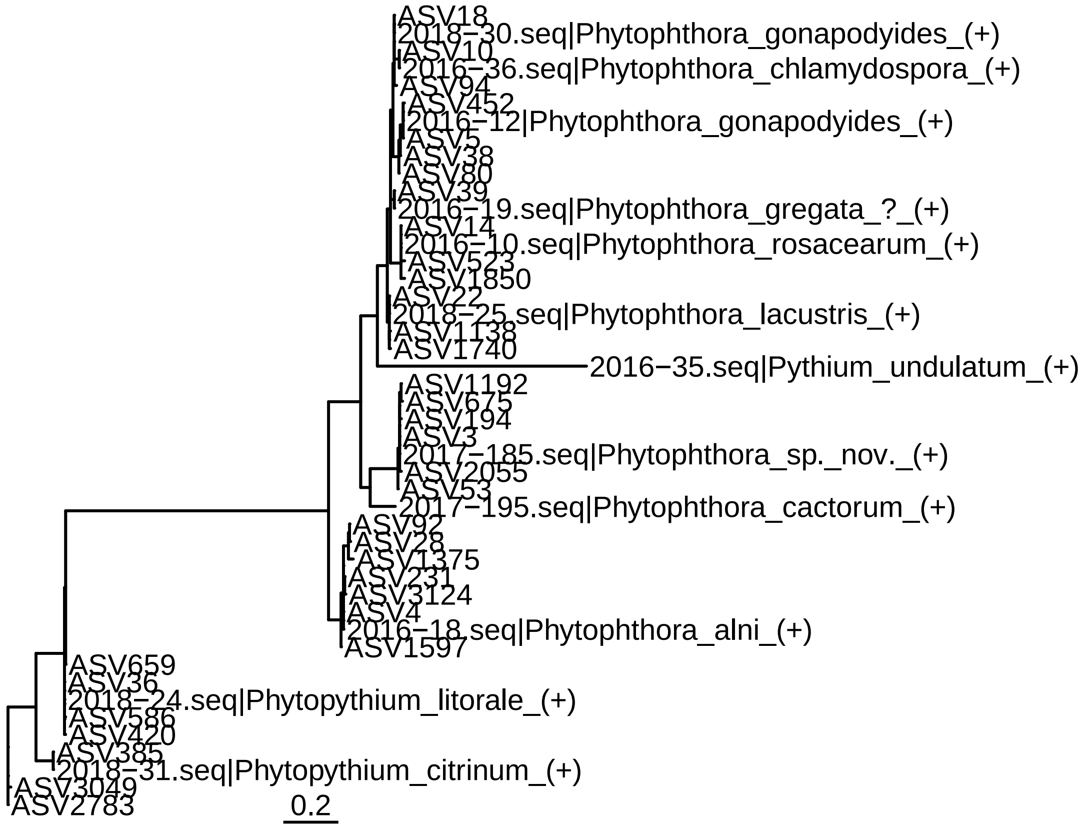


```r
cat("Chunk successfully run")
```

```
## Chunk successfully run
```

Data overview This chunk generates an overview over read counts for all samples and additional info on the data (Supplementary Fig. 1). ASV counts are summed up for all samples in the provided phyloseq object and assigned a rank based on this sum. The sample with the highest amount of total ASV counts will receive rank 1, the one with the second highest rank 2 and so on. A bar plot is generated, plotting the

total ASV counts against the sample rank with color filling by run and a dashed line indicating the chosen cutoff defined in minASVcount (Parameter chunk).

```
## CHANGE ME to change the width (in cm) of the output.
wp = 25
# CHANGE ME to change the height (in cm) of the output.
hp = 13.5
# CHANGE ME to change the resolution (in dpi) of the output.
res = 150

# Rank all samples, including those that were discarded from 'ps',
# but excluding controls
all <- cuphyr::make_ranked_sums(ps.raw, myset = "Total")

# This calculates an average read count for all samples that can be added to
# the plot as e.g. a dashed line or text
avg <- mean(all$Abundance)
# Calculate per group averages in a basic table that can be included in the
# 'overview' plot.
avgs_group <- all %>%
  group_by(Run, Bait) %>%
  summarise("Mean ASV counts" = mean(Abundance)) %>%
  dplyr::rename(Sampling = Bait) %>%
  mutate(Sampling = str_replace(Sampling, "Enriched", "After enrichment"))
pall <- ggplot(data = all, aes(y = Abundance, x = Samples))

# plot figure
overview <- ggplot(data = all, aes(x = Rank, y = Abundance, fill = Run)) +
  geom_col() + my_scale_fill +
  geom_hline(yintercept = minASVcount, linetype = "dashed") +
  ylab("Total ASV counts ('reads')") #+
  ## optional table overlay containing summary values in figure
  #annotation_custom(tableGrob(avgs_group, rows = NULL,
  #                             theme = ttheme_minimal(base_size = 8)),
  #                   xmin = 40, xmax = 110,
  #                   ymin = 75000, ymax = 100000)

# Alternative, more compact, presentation of the 'overview' plot.
# Not included in the manuscript
overview_compact <- ggplot(data = all,
                           aes(x = Run, y = Abundance, color = Run)) +
  geom_boxplot() + geom_jitter(aes(shape = Bait)) + my_scale_col +
  geom_hline(yintercept = minASVcount, linetype = "dashed") +
  ylab("Total ASV counts ('reads')") +
  theme(axis.text.x = element_blank(),
        #axis.text.x = element_text(angle = 20, hjust = 1, vjust = 1),
        axis.title.y = element_blank(),
        axis.ticks.x = element_blank())

# Combination of both plots, not included in the manuscript
overview_combo <- ggarrange(overview, overview_compact, widths = c(2,1),
                            labels = c("A", "B"), align = "hv")

# Also give the number of ASVs, oomycete ASVs and calculate the percentage of
```

```r
# ASVs identified as oomycetes
oomlist <- cuphyr::list_subset_ASVs(subv = c("Oomycetes"), taxlvlsub = "Class")

# Save figure
save_plot(overview, plot_name = "Overview_SupFig1")
#save_plot(overview_combo, plot_name = "Overview_combo_Supfig1alt")

overview
```

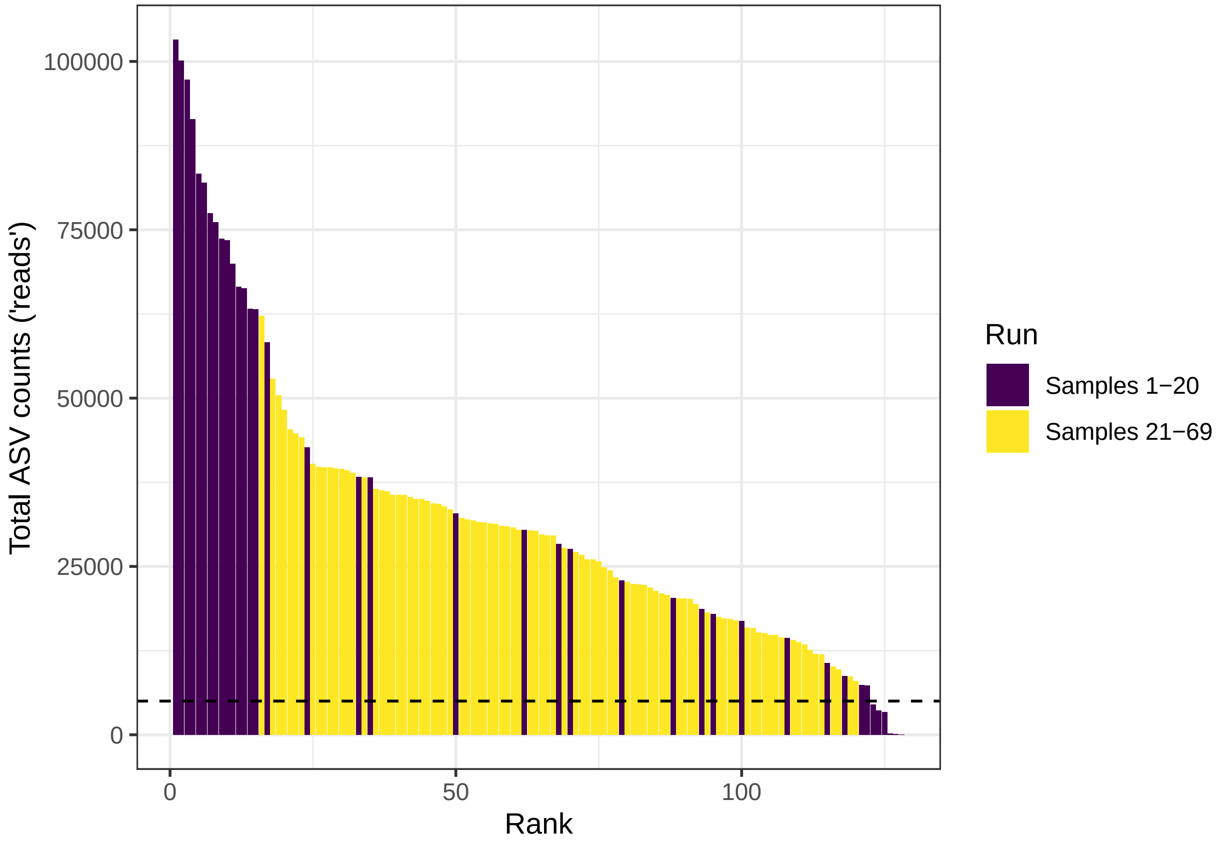


```r
cuphyr::summarise_physeq(physeq = ps,
                         ASV_sublist = oomlist,
                         sublist_id = "Oomycetes", samp_names = FALSE)
```

```
## There are 5195 ASVs in the phyloseq object 'ps'.
## Of this, 1832 belong to the provided subset (Oomycetes), representing 71.84 percent of abundance per
```

```r
cat("Chunk successfully run")
```

```
## Chunk successfully run
```

Top N ASVs/taxa Bar plot This chunk plots abundance of the Top n ASVs and taxa at a given level as a bar plot, giving an insight into the presence of the n ASV and most common taxa for the primary and secondary parameters. In revision 1 of the manuscript, the top 5 classes and top 10 genera per sample are shown in Supplementary fig. 3.

```r
# CHANGE ME to the amount of top taxlvl to plot (e.g. 'numt = 20' plots Top20
# Taxa at taxlvl)
numt <- 5

# CHANGE ME to the amount of top taxa you want to plot (e.g. 'numt2 = 25' plots
# Top25 Taxa at taxlvl2)
numt2 <- 10

# CHANGE ME to the primary parameter of interest (x-axis). Accepted values are
# the column headers in the descriptor file.
primaryPar <- "Soil"

## CHANGE ME to the secondary parameter of interest (panels). Accepted values
# are the column headers in the descriptor file.
secondaryPar <- "Bait"

## CHANGE ME to the taxonomic level of interest (color coding). Accepted values
# are available taxonomic levels. 'taxlvl' should be higher than 'taxlvl2', e.g.
# Class and Genus
taxlvl <- "Class"
taxlvl2 <- "Genus"

# Adds formatted legend labels for the specific output in the manuscript.
# WARNING! Will overwrite actual values and needs to be turned off/adjusted
# manually when data is updated
customLabels <- TRUE

## CHANGE ME to change the width (in cm) of the output.
wp <- 18
# CHANGE ME to change the height (in cm) of the output.
hp <- 15
# CHANGE ME to change the resolution (in dpi) of the output.
res <- 150

# Get total number of reads and number of Pythium/Phytophthora reads (without
# control samples!) to find out percentage of Pyhium and Phytophthora
total_reads_no_contr <- ps_tbl %>%
                        dplyr::filter(Pass == "Yes") %>%
                        dplyr::mutate(sumAbu = sum(Abundance)) %>%
                        select(sumAbu) %>%
                        unique() %>%
                        unlist() %>%
                        unname()

pythium_reads_no_contr <- ps_tbl %>%
                          dplyr::filter(Pass == "Yes") %>%
                          dplyr::group_by(Genus) %>%
                          summarise(sumAbu = sum(Abundance)) %>%
                          dplyr::filter(Genus == "Pythium") %>%
                          select(sumAbu) %>%
                          unlist() %>%
                          unname()
```

```r
phytoph_reads_no_contr <- ps_tbl %>%
                          dplyr::filter(Pass == "Yes") %>%
                          dplyr::group_by(Genus) %>%
                          summarise(sumAbu = sum(Abundance)) %>%
                          dplyr::filter(Genus == "Phytophthora") %>%
                          select(sumAbu) %>%
                          unlist() %>%
                          unname()

prctg_pyth_phyt_str <- paste0("\n\nTotal number of reads in the samples: ",
                              total_reads_no_contr ,
                          ", number of Pythium reads: ",
                          pythium_reads_no_contr,
                          ", number of Phytophthora reads: ",
                          phytoph_reads_no_contr,
                          "\nPercentage of Pythium total: ",
                          pythium_reads_no_contr/total_reads_no_contr*100,
                          "%\nPercentage of Phytophthora total: ",
                          phytoph_reads_no_contr/total_reads_no_contr*100, "%")

# Most abundant at specified levels, phyloseq objects
ps.topnt <- cuphyr::abundant_tax_physeq(
  physeq = ps.trans, lvl = taxlvl, top = numt,
  ignore_na = TRUE, silent = FALSE)
```

```
##
## The top 5 most abundant annotated groups at the taxonomic level 'Class' are:
## Oomycetes
## Chrysophyceae
## 'uncultured fungus'
## Bacillariophyceae
## Dinophyceae
```

```r
ps.topnt2 <- cuphyr::abundant_tax_physeq(
  physeq = ps.trans,lvl = taxlvl2, top = numt2,
  ignore_na = TRUE, silent = FALSE)
```

```
##
## The top 10 most abundant annotated groups at the taxonomic level 'Genus' are:
## Pythium
## Pedospumella
## Phytophthora
## Globisporangium
## 'uncultured fungus'
## 'Spumella-like'
## Pythiogeton
## Haptoglossa
## Aphanomyces
## Pythiopsis
```

```r
# Abundance lists (sorted by abundance)
taxlist_topnt1 <- cuphyr::abundant_tax_physeq(
```

```
  physeq = ps.trans, lvl = taxlvl, top = numt,
  output_format = "tops", ignore_na = TRUE)
taxlist_topnt2 <- cuphyr::abundant_tax_physeq(
  physeq = ps.trans, lvl = taxlvl2, top = numt2,
  output_format = "tops", ignore_na = TRUE)

# tidy evaluation translation magic (needed so the dplyr functions that extract
# 'tax_lookup' below can parse the strings from the variable)
lvl1 <- sym(taxlvl)
lvl2 <- sym(taxlvl2)
lvl1 <- enquo(lvl1)
lvl2 <- enquo(lvl2)

#make lookup table to guide coloring
tax_lookup <- ps_tbl %>%
                    group_by(!!lvl1, !!lvl2) %>%
                    summarise() %>%
                    filter(!!lvl2 %in% taxlist_topnt2)

# Define fixed color schemes for classes for consistency between plots. This is
# a color scheme specified for this particular published figure. Can be used for
# orientation in general cases.
# Background colors to be overwritten for special cases
colors_lvl1 <- viridis(length(taxlist_topnt1),
                       option = "D", begin = 1 , end = 0.5)
names(colors_lvl1) <- taxlist_topnt1
colors_lvl2 <- viridis(length(taxlist_topnt2),
                       option = "D", begin = 1 , end = 0.5)
names(colors_lvl2) <- taxlist_topnt2
colors_combo <- c(colors_lvl1, colors_lvl2)

#Special cases
colors_combo["Chrysophyceae"] <- viridis_yellows[2]
colors_combo["Pedospumella"] <- viridis_yellows[1]
colors_combo["'Spumella-like'"] <- viridis_light[1]

colors_combo["Oomycetes"] <- viridis_reds[4]
colors_combo["Phytophthora"] <- viridis_reds[1]
colors_combo["Pythium"] <- viridis_reds[4]
colors_combo["Aphanomyces"] <- viridis_blues[1]
colors_combo["Globisporangium"] <- "#4C0000FF"
colors_combo["Pythiogeton"] <- viridis_blues[2]
colors_combo["Saprolegnia"] <- viridis_blues[4]

#Format legend labels
if (customLabels) {
  labelling_lvl2 <- c(expression(paste(italic("Spumella"), "-like")),
  "uncultured fungus", expression(italic("Aphanomyces"),
  italic("Globisporangium"), italic("Haptoglossa"), italic("Pedospumella"),
  italic("Phytophthora"), italic("Pythiogeton"), italic("Pythiopsis"),
  italic("Pythium")))
}else{
  labelling_lvl2 <- names(colors_lvl2) %>% sort()
```

```r
}

#Re-label the facets
enrich_labs <- c("Before enrichment", "After enrichment")
names(enrich_labs) <- c("Before enrichment", "Enriched")

theme_bar <- theme(legend.position = "bottom",
                   legend.key.size = unit(0.4, "cm"),
                   legend.spacing.x = unit(0.3, 'cm'),
                   text = element_text(size = 9),
                   strip.text.x = element_text(size = 10),
                   strip.background = element_blank(),
                   axis.text.x = element_text(angle = 0, hjust = 0.5),
                   legend.text.align = 0)

topnt <- plot_bar(ps.topnt, x = primaryPar, fill = taxlvl,
                  title = paste("Top", numt, "Classes")) +
  facet_wrap(paste0("~", secondaryPar),
             labeller = labeller(Bait = enrich_labs)) +
  scale_x_discrete(breaks = c("S05" ,"S15", "S25", "S35", "S45" ,"S55")) +
  scale_fill_manual(values = colors_combo) +
  ylab("Relative abundance") +
  theme_bar +
  xlab("Soil samples S01-S64") +
  ylim(0, 1)

topnt_summary <- cuphyr::summarise_physeq(physeq = ps,
                                          ASV_sublist = taxa_names(ps.topnt),
                                          sublist_id =
                                            paste0("top ", numt, " ", taxlvl),
                                          samp_names = FALSE)
```

```
## There are 5195 ASVs in the phyloseq object 'ps'.
## Of this, 3706 belong to the provided subset (top 5 Class), representing 96.10 percent of abundance p
```

```r
topnt2 <- plot_bar(ps.topnt2, x = primaryPar, fill = taxlvl2,
                   title = paste("Top", numt2, "Genera")) +
  facet_wrap(paste0("~", secondaryPar),
             labeller = labeller(Bait = enrich_labs)) +
  scale_x_discrete(breaks = c("S05" ,"S15", "S25", "S35", "S45" ,"S55")) +
  scale_fill_manual(values = colors_combo, labels = labelling_lvl2) +
  ylab("Relative abundance") +
  theme_bar + xlab("Soil samples S01-S64") +
  ylim(0, 1)

topnt2_summary <- cuphyr::summarise_physeq(physeq = ps,
                                           ASV_sublist = taxa_names(ps.topnt2),
                                           sublist_id =
                                             paste0("top ", numt2, " ", taxlvl2),
                                           samp_names = FALSE)
```

```
## There are 5195 ASVs in the phyloseq object 'ps'.
## Of this, 2649 belong to the provided subset (top 10 Genus), representing 86.63 percent of abundance p
```

```
#Combine the plots
combobar1 <- ggarrange(topnt, topnt2, nrow = 2,
                       labels = c("A", "B"), align = "hv")

#Save
save_plot(combobar1, plot_name = "Topn_barplot_Suppfig3")

#Print to standard out
cat(topnt_summary, "\n\n", topnt2_summary)
```

```
cat(prctg_pyth_phyt_str)
```

```
##
##
## Total number of reads in the samples: 4113471, number of Pythium reads: 2003562, number of Phytophth
## Percentage of Pythium total: 48.7073325665843%
## Percentage of Phytophthora total: 6.40254908810588%
```

```
combobar1
```

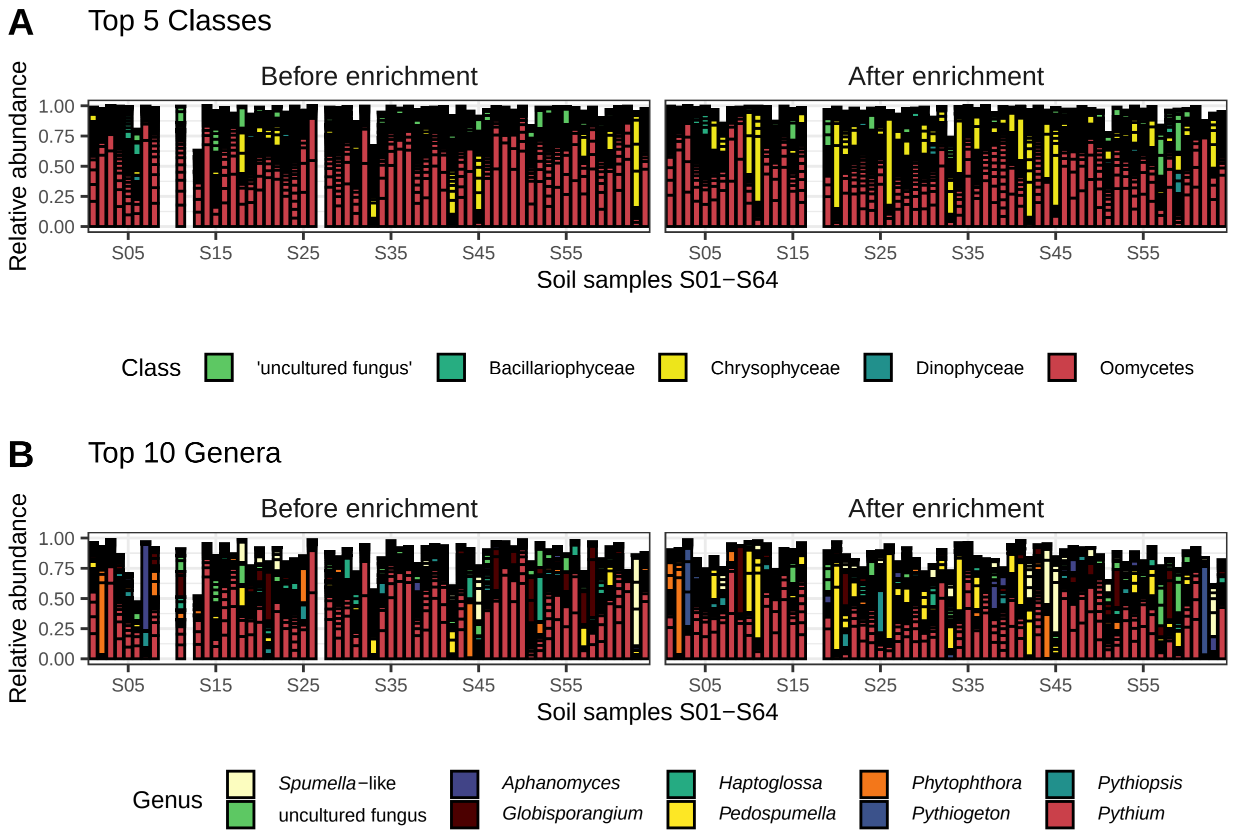


```
cat("Chunk successfully run")
```

```
## Chunk successfully run
```

Top N ASVs/taxa means Bar plot (Rev1) This chunk plots abundance of the Top n ASVs and taxa at a given level as a bar plot, giving an insight into the presence of the n ASV and most common taxa for the primary and secondary parameters. This plot does not show the per-sample distribution given in the chunk above but instead shows mean presence over all samples. In the manuscript, the top 5 classes and top 10 genera are shown in Fig. 1.

```
# CHANGE ME to the amount of top taxlvl to plot (e.g. 'numt = 20' plots Top20
# Taxa at taxlvl)
numt <- 5

# CHANGE ME to the amount of top taxa you want to plot (e.g. 'numt2 = 25' plots
# Top25 Taxa at taxlvl2)
numt2 <- 10

# CHANGE ME to the primary parameter of interest (x-axis). Accepted values are
# the column headers in the descriptor file.
primaryPar <- "Soil"

## CHANGE ME to the secondary parameter of interest (panels). Accepted values
# are the column headers in the descriptor file.
secondaryPar <- "Bait"

## CHANGE ME to the taxonomic level of interest (color coding). Accepted values
# are available taxonomic levels. 'taxlvl' should be higher than 'taxlvl2', e.g.
# Class and Genus
taxlvl <- "Class"
taxlvl2 <- "Genus"

# Adds formatted legend labels for the specific output in the manuscript.
# WARNING! Will overwrite actual values and needs to be turned off/adjusted
# manually when data is updated
customLabels <- TRUE

## CHANGE ME to change the width (in cm) of the output.
wp <- 18
# CHANGE ME to change the height (in cm) of the output.
hp <- 10
# CHANGE ME to change the resolution (in dpi) of the output.
res <- 150

# Define fixed color schemes for classes for consistency between plots. This is
# a color scheme specified for this particular published figure. Can be used for
# orientation in general cases.
# Background colors to be overwritten for special cases
colors_lvl1 <- viridis(length(taxlist_topnt1),
                       option = "D", begin = 1 , end = 0.5)
names(colors_lvl1) <- taxlist_topnt1
colors_lvl2 <- viridis(length(taxlist_topnt2),
                       option = "D", begin = 1 , end = 0.5)
names(colors_lvl2) <- taxlist_topnt2
colors_combo <- c(colors_lvl1, colors_lvl2)

# Special cases
colors_combo["Chrysophyceae"] <- viridis_yellows[2]
```

```r
colors_combo["Pedospumella"] <- viridis_yellows[1]
colors_combo["'Spumella-like'"] <- viridis_light[1]

colors_combo["Oomycetes"] <- viridis_reds[4]
colors_combo["Phytophthora"] <- viridis_reds[1]
colors_combo["Pythium"] <- viridis_reds[4]
colors_combo["Aphanomyces"] <- viridis_blues[1]
colors_combo["Globisporangium"] <- "#4C0000FF"
colors_combo["Pythiogeton"] <- viridis_blues[2]
colors_combo["Saprolegnia"] <- viridis_blues[4]

# Format legend labels
if (customLabels) {
  labelling_lvl2 <- c(expression(paste(italic("Spumella"), "-like")),
  "uncultured fungus", expression(italic("Aphanomyces"),
  italic("Globisporangium"), italic("Haptoglossa"), italic("Pedospumella"),
  italic("Phytophthora"), italic("Pythiogeton"), italic("Pythiopsis"),
  italic("Pythium")))
}else{
  labelling_lvl2 <- names(colors_lvl2) %>% sort()
}

# Define theme for the plot
theme_bar_means <- theme(legend.position = "right",
                   legend.key.size = unit(0.4, "cm"),
                   legend.spacing.x = unit(0.3, 'cm'),
                   text = element_text(size = 10),
                   strip.text.x = element_text(size = 10),
                   strip.background = element_blank(),
                   axis.text.x = element_text(angle = 0, hjust = 0.5),
                   legend.text.align = 0,
                   axis.title.x = element_blank())

# Averages before and after enrichment for plotting
group_avgs <- ps_tr_tbl %>%
  group_by(Bait, OTU) %>%
  summarise(avg_abu_rel = mean(Rel_Abundance)) %>%
  arrange(desc(avg_abu_rel))

group_avgs <- group_avgs %>%
  left_join(ps_tr_tbl, by = c("OTU", "Bait")) %>%
  select(!(Sample:Type)) %>%
  select(!(sum_Abundance:Prcnt_Abundance)) %>%
  unique()

# Restrict to the chosen taxlvls
group_avgs_taxlvl <- group_avgs %>%
  filter(!!ensym(taxlvl) %in% taxlist_topnt1)

group_avgs_taxlvl2 <- group_avgs %>%
  filter(!!ensym(taxlvl2) %in% taxlist_topnt2)

# plot
```

```
lvl_means <- ggplot(data = group_avgs_taxlvl,
                      aes(x = Bait, y = avg_abu_rel, fill = Class),
                      labeller()) +
  geom_col() +
  scale_fill_manual(values = colors_combo) +
  scale_x_discrete(labels = c("Before enrichment" = "Before enr.",
           "Enriched" = "After enr.")) +
  ylab("Relative abundance") +
  theme_bar_means +
  ylim(0,1)

lvl2_means <- ggplot(data = group_avgs_taxlvl2,
                      aes(x = Bait, y = avg_abu_rel, fill = Genus)) +
  geom_col() +
  scale_fill_manual(values = colors_combo, labels = labelling_lvl2) +
  scale_x_discrete(labels = c("Before enrichment" = "Before enr.",
           "Enriched" = "After enr.")) +
  ylab("Relative abundance") +
  theme_bar_means +
  ylim(0,1)

#Combine the plots
combobar2 <- ggarrange(lvl_means, lvl2_means, ncol = 2,
                       labels = c( "A", "B"), align = "v")

#Save
save_plot(combobar2, plot_name = "Topn_barplot_Fig1_Rev1")

#Print to standard out
combobar2
```

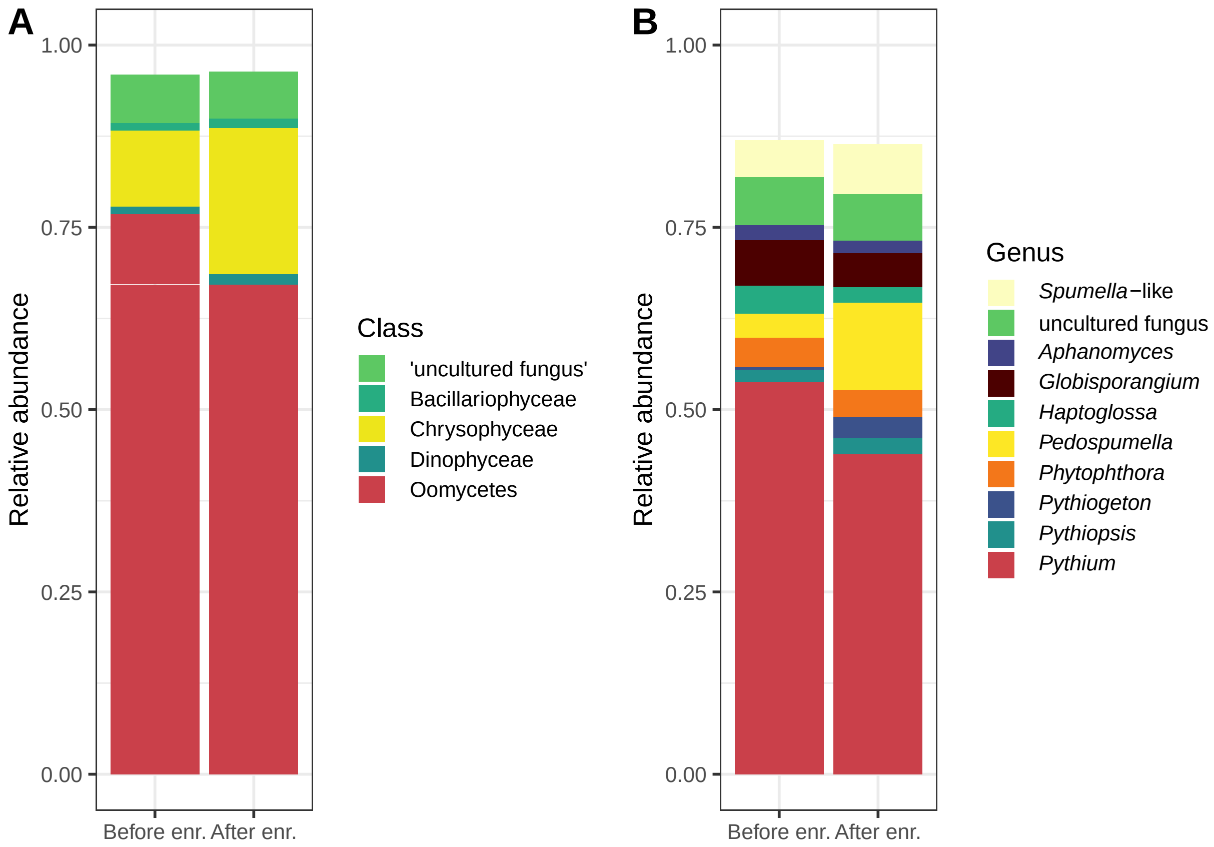


```
cat("Chunk successfully run")
```

```
## Chunk successfully run
```

Functional classification using the FUNGuild database This chunk imports the FUNGuild database based on code by S. Faye Smith (2018), then classifies and plots all oomycete genera in the data set (Fig. 2).

```
# Plot save dimensions
wp <- 18
hp <- 13.5
res <- 150

# Adds formatted legend labels for the specific output in the manuscript.
# WARNING! Will overwrite actual values and needs to be turned off/adjusted
# manually when data is updated
customLabels <- TRUE

# Custom color coding to emphasize pp genera, particularly Pythium and
# Phytophthora
colors_genera_pp <- viridis(length(genera_pp), option = "D",
                            begin = 0 , end = 0.8)
names(colors_genera_pp) <- genera_pp
colors_genera_other <- viridis(length(genera_other), option = "D",
```

```r
                              begin = 1 , end = 0.9)
names(colors_genera_other) <- genera_other
colors_oomyc_genera <- c(colors_genera_pp, colors_genera_other)

# Sort alphabetically by vector names to avoid mixing up color assignments
# (origin of issue not resolved)
colors_oomyc_genera <- colors_oomyc_genera[order(names(colors_oomyc_genera))]
colors_oomyc_genera["Phytophthora"] <- viridis_reds[1]
colors_oomyc_genera["Pythium"] <- viridis_reds[4]

#define ggplot theme (shared between genus and species level plots)
shared_theme <- theme(legend.key.size = unit(3, "mm"),
                      legend.position = "none",
                      axis.title.x = element_blank(),
                      plot.margin = margin(20,10,10,10),
                      legend.text.align = 0,
                      text = element_text(size = 10))

shared_theme_abu <- theme(legend.key.size = unit(3, "mm"),
                          legend.position = "none",
                          axis.title.x = element_blank(),
                          plot.margin = margin(20,10,10,10),
                          legend.text.align = 0,
                          text = element_text(size = 10))

#Format legend labels
if(customLabels){
  labelling <- c(expression(paste("uncultured ", italic("Apodachlya"))),
    expression(paste("uncultured ", italic("Lagenidium"))),
    expression(paste("uncultured ", italic("Oomycetes"))),
    expression(paste("uncultured ", italic("Phytophthora"))),
    expression(paste("uncultured ", italic("Pythium"))),
    expression(italic("Achlya"), italic("Albugo"), italic("Aphanomyces"),
    italic("Apodachlya"), italic("Atkinsiella"), italic("Bremia"),
    italic("Brevilegnia"), italic("Dictyuchus"), italic("Eurychasma"),
    italic("Geolegnia"), italic("Globisporangium"), italic("Halocrusticida"),
    italic("Haptoglossa"), italic("Hyaloperonospora"), italic("Lagena"),
    italic("Lagenidium"), italic("Leptolegnia"), italic("Myzocytiopsis"),
    italic("Paralagenidium"), italic("Peronospora"), italic("Phragmosporangium"),
    italic("Phytophthora"), italic("Phytopythium"), italic("Pilasporangium"),
    italic("Plectospira"), italic("Pustula"), italic("Pythiogeton"),
    italic("Pythiopsis"), italic("Pythium"), italic("Salilagenidium"),
    italic("Saprolegnia"), italic("Thraustotheca"), italic("Wilsoniana")), NA)
}else{
  labelling <- unique(ASVs_funguild$Genus)
}

guild_g <- ggplot(ASVs_funguild, aes(x = guild_genus, fill = Genus)) +
  geom_bar() + shared_theme +
  scale_fill_manual(values = colors_oomyc_genera,
                    na.value = "grey", labels = labelling) +
  ylab("Number of ASVs") + ylim(0,1500)
```

```
guild_s <- ggplot(ASVs_funguild, aes(x = guild_species, fill = Genus)) +
  geom_bar() + shared_theme +
  scale_fill_manual(values = colors_oomyc_genera,
                    na.value = "grey", labels = labelling) +
  ylab("Number of ASVs") + ylim(0,1500)

guild_g_abu <- ggplot(ASVs_funguild, aes(x = guild_genus,
                                         y = Abundance_sum/1000000,
                                         fill = Genus)) +
  geom_col() + shared_theme_abu +
  scale_fill_manual(values = colors_oomyc_genera,
                    na.value = "grey", labels = labelling) +
  ylab("Abundance in M reads") + ylim(0,2.5)

guild_s_abu <- ggplot(ASVs_funguild, aes(x = guild_species,
                                         y = Abundance_sum/1000000,
                                         fill = Genus)) +
  geom_col() + shared_theme_abu +
  scale_fill_manual(values = colors_oomyc_genera,
                    na.value = "grey", labels = labelling) +
  ylab("Abundance in M reads") + ylim(0,2.5)

guilds <- ggarrange(guild_g, guild_g_abu, guild_s,
                    guild_s_abu, nrow = 2, ncol = 2,
                    common.legend = TRUE, legend = "right" ,align = "hv",
                    labels = c("Genus level", "", "Species level", ""),
                    font.label = list(size = 12))
guilds
```

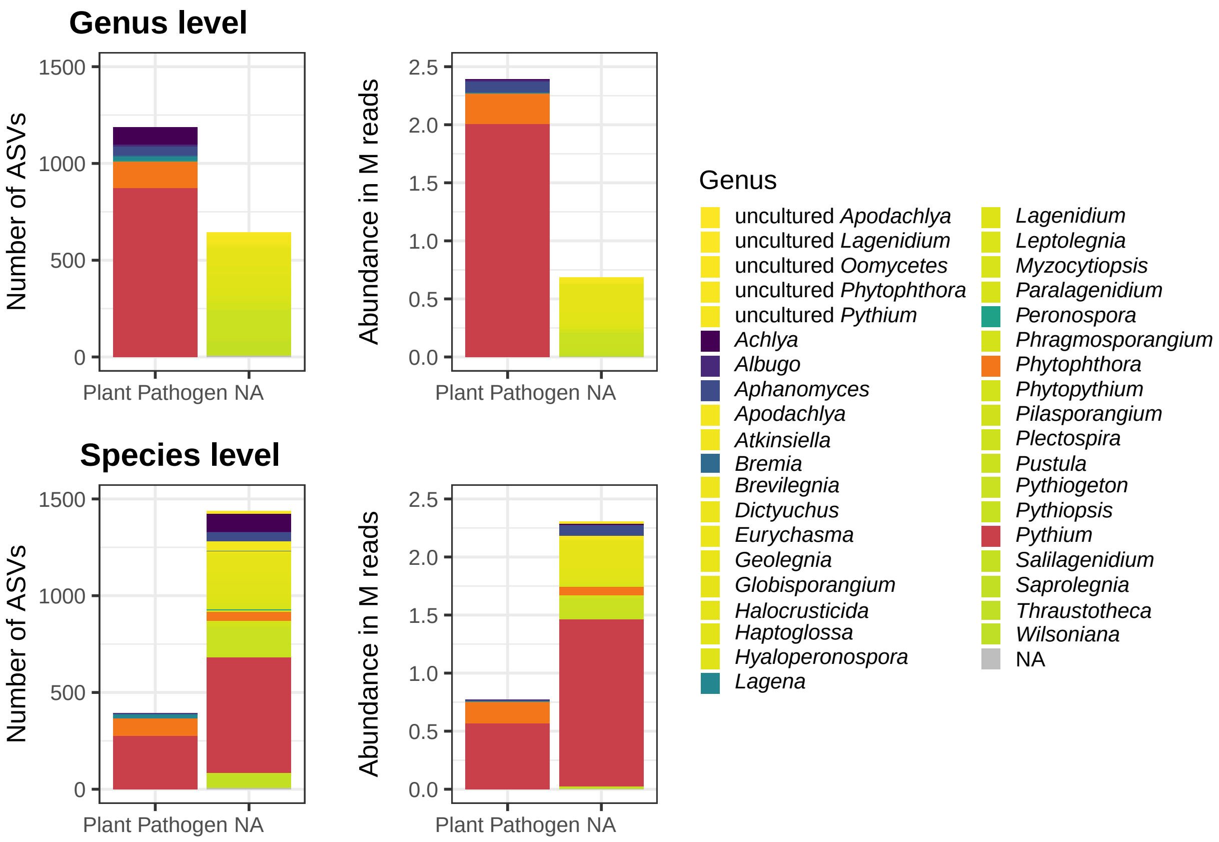


```
save_plot(guilds, plot_name = "FunGuild_combo_Fig2")

cat("Chunk successfully run")
```

```
## Chunk successfully run
```

Extract summary table with sample-specific information This chunk extracts the base data used in Table 1 of the revised manuscript

```
# Filter Oomycetes with Abundance > 0 from large transformed ps tibble
tbl_overview <- filter(ps_tr_tbl, str_detect(Class, 'Oomycetes')) %>%
  filter(Abundance > 0)

# Extract ASVs annotated as 'Plant Pathogen' by FUNGuild to annotate overview
ASVs_PPP <- filter(ASVs_funguild, str_detect(guild_genus, 'Plant Pathogen')) %>%
  select(OTU, guild_genus) %>% unique()

# Fuse the two objects from above and reduce to relevant columns
tbl_overview <- left_join(tbl_overview, ASVs_PPP, by = "OTU") %>%
  dplyr::select(OTU, Soil, Bait, Prcnt_Abundance,
                guild_genus, Type, Country, Run) %>%
  unique()

# Count distinct Oomycete ASVs and sum up per sample abundance percentage
```

```
count_Oomycs <- tbl_overview %>%
  dplyr::group_by(Soil, Bait) %>%
  summarise(number_Oomycete_ASVs = n_distinct(OTU),
            sum_Prcnt_Oomycetes = sum(Prcnt_Abundance))

# Count distinct PPP ASVs and sum up per sample abundance percentage
count_PPP <- tbl_overview %>%
  filter(OTU %in% ASVs_PPP$OTU) %>%
  dplyr::group_by(Soil, Bait) %>%
  summarise(number_PPP_ASVs = n_distinct(OTU),
            sum_Prcnt_PPP = sum(Prcnt_Abundance))

# Combine counts with big tibble and reformat column headers, round percentages
# to one decimal
tbl_overview_compl <- tbl_overview %>%
  select(-OTU, -Prcnt_Abundance, -guild_genus) %>%
  unique() %>%
  left_join(count_Oomycs) %>%
  left_join(count_PPP) %>%
  arrange(Soil, Bait) %>%
  dplyr::rename(Sampling = Bait, "Sample type" = Type, "Sequencing run" = Run,
                "Exporting country" = Country,
                "Oomycete ASVs" = number_Oomycete_ASVs,
                "% Oomycete ASVs" = sum_Prcnt_Oomycetes,
                "PPP ASVs" = number_PPP_ASVs,
                "% PPP ASVs" = sum_Prcnt_PPP) %>%
  mutate(Sampling = str_replace(Sampling, "Enriched", "After enrichment")) %>%
  mutate(across(c(7,9), round, 1))

# export as csv
write_csv(tbl_overview_compl, path = file.path(path, "Rev1_table_1.csv"))

# Optional: export as xlsx, requires 'writexl' package
#writexl::write_xlsx(tbl_overview_compl, path = file.path(path,
#                                                  "Rev1_table_1.xlsx"),
#                    format_headers = FALSE)
```

Rank samples by ASV count of classes This chunk generates all panels of Fig. 3, in which the relative abundances of taxonomic classes of interest are summarized. Relative abundances are summed up for all samples in the provided phyloseq objects and assigned a rank based on this sum. The sample with the highest amount of total ASV counts will receive rank 1, the one with the second highest rank 2 and so on. A bar plot is generated, plotting relative abundance against the sample rank with the bars being colored according to the taxonomic. This is supposed to provide a quick overview of relative abundance for the taxonomic class subsets.

```
## CHANGE ME to change the width (in cm) of the output.
wp <- 17.5
# CHANGE ME to change the height (in cm) of the output.
hp <- 15
# CHANGE ME to change the resolution (in dpi) of the output.
res <- 150

# calculate averages for display in graphs
```

```r
avgNames <- c("PPUntr", "PPBait","ChrUntr", "ChrBait",
              "O-PUntr", "O-PBait", "OthUntr", "OthBait")
avgs <- c(mean(ranksUntrPP$Abundance),
          mean(ranksBaitPP$Abundance),
          mean(ranksUntrAlgae$Abundance),
          mean(ranksBaitAlgae$Abundance),
          mean(ranksUntrOwoP$Abundance),
          mean(ranksBaitOwoP$Abundance),
          mean(rankedOthersUntr$OtherASVcounts),
          mean(rankedOthersBait$OtherASVcounts))

names(avgs) <- avgNames

# multiply to transform relative abundances into avg percentages and round to
# one decimal
avgs <- avgs*100
avgs <- format(round(avgs, 1), nsmall = 1)

# Define fixed color schemes for consistency between plots
plotvars <- c("Chrysophyceae", "All oomycetes",
              "Other oomycetes", "Other classes", "PP")

# Just to catch others/exceptions, make a general palette that will be
# overwritten below
plotPalette <- viridis(length(plotvars))
names(plotPalette) <- plotvars
plotPalette["Other classes"] <- "#C0C0C0"
plotPalette["Chrysophyceae"] <- viridis_yellows[2]
plotPalette["PP"] <- viridis_reds[4]
plotPalette["Other oomycetes"] <- viridis_reds[1]
plotPalette["All oomycetes"] <- viridis_reds[1]

# plot total ASV counts, sorted by rank and color by class.
AllBait <- ggplot(data = OthersRankPP,
                  aes(x = Rank, y = Abundance, fill = Set))
AllUntr <- ggplot(data = UntrOthersRankPP,
                  aes(x = Rank, y = Abundance, fill = Set))

customTheme <- theme(legend.position = "none",
                     axis.title = element_blank(),
                     title = element_text(size = 6))
customThemeA <- theme(legend.position = "none",
                      legend.title = element_blank(),
                      legend.key.size = unit(5,"mm"),
                      axis.title.x = element_blank())

xUpperLim <- nrow(OthersRankPP) + 1
ytextl = 0.05
xtextl = 15
ytexts = 0.75
xtexts = 45
middleline <- geom_blank() #geom_hline(yintercept = 0.5,
                           # linetype="dashed", color="black")
```

```
pAllBait <- AllBait + geom_col() +
  geom_col(data = AlgaeInvRankOom) +
  geom_col(data = OomRankPP) +
  geom_col(data = ranksBaitPP) +
  scale_fill_manual(values = plotPalette) +
  ggtitle("After enrichment") + xlab("Sample rank") +
  labs(fill = "") + ylim(0,1) + xlim(0,xUpperLim) +
  customThemeA + middleline +
  annotate(geom = 'text', label = paste("Mean PP", avgs["PPBait"], "%"),
           x = xtextl, y = ytextl) +
  ylab("Rel. abundance")

pAllUntr <- AllUntr + geom_col() +
  geom_col(data = UntrAlgaeInvRankOom) +
  geom_col(data = UntrOomRankPP) +
  geom_col(data = ranksUntrPP) +
  scale_fill_manual(values = plotPalette) +
  ggtitle("Before enrichment") + xlab("Sample rank") +
  labs(fill = "") + ylim(0,1) + xlim(0,xUpperLim) +
  customThemeA + middleline +
  annotate(geom = 'text', label = paste("Mean PP", avgs["PPUntr"], "%"),
           x = xtextl, y = ytextl) +
  ylab("Rel. abundance")

AlgBait <- ggplot(data = ranksBaitAlgae,
                  aes(x = Rank, y = Abundance, fill = Set)) + geom_col() +
  scale_fill_manual(values = plotPalette) +
  ggtitle("Chrysophyceae - after enrichment") +
  xlim(0,xUpperLim) + customTheme + middleline +
  annotate(geom = 'text', label = paste("Mean", avgs["ChrBait"], "%"),
           x = xtexts, y = ytexts) +
  scale_y_continuous(breaks = c(0,0.5,1), limits = c(0,1))

OomBait <- ggplot(data = ranksBaitOwoP,
                  aes(x = Rank, y = Abundance, fill = Set)) + geom_col() +
  scale_fill_manual(values = plotPalette) +
  ggtitle("Other oomycetes - after enrichment") +
  xlim(0,xUpperLim) + customTheme + middleline +
  annotate(geom = 'text', label = paste("Mean", avgs["O-PBait"], "%"),
           x = xtexts, y = ytexts) +
  scale_y_continuous(breaks = c(0,0.5,1), limits = c(0,1))

OthBait <- ggplot(data = rankedOthersBait,
                  aes(x = Rank, y = OtherASVcounts, fill = Set)) + geom_col() +
  scale_fill_manual(values = plotPalette) +
  ggtitle("Other classes - after enrichment") +
  xlim(0,xUpperLim) + customTheme + middleline +
  annotate(geom = 'text', label = paste("Mean", avgs["OthBait"], "%"),
           x = xtexts, y = ytexts) +
  scale_y_continuous(breaks = c(0,0.5,1), limits = c(0,1))

AlgUntr <- ggplot(data = ranksUntrAlgae,
                  aes(x = Rank, y = Abundance, fill = Set)) + geom_col() +
```

```
  scale_fill_manual(values = plotPalette) +
  ggtitle("Chrysophyceae - before enrichment") +
  xlim(0,xUpperLim) + customTheme + middleline +
  annotate(geom = 'text', label = paste("Mean", avgs["ChrUntr"], "%"),
           x = xtexts, y = ytexts) +
  scale_y_continuous(breaks = c(0,0.5,1), limits = c(0,1))

OomUntr <- ggplot(data = ranksUntrOwoP,
                  aes(x = Rank, y = Abundance, fill = Set)) + geom_col() +
  scale_fill_manual(values = plotPalette) +
  ggtitle("Other oomycetes - before enrichment") +
  xlim(0,xUpperLim) + customTheme + middleline +
  annotate(geom = 'text', label = paste("Mean", avgs["O-PUntr"], "%"),
           x = xtexts, y = ytexts) +
  scale_y_continuous(breaks = c(0,0.5,1), limits = c(0,1))

OthUntr <- ggplot(data = rankedOthersUntr,
                  aes(x = Rank, y = OtherASVcounts, fill = Set)) + geom_col() +
  scale_fill_manual(values = plotPalette) +
  ggtitle("Other classes - before enrichment") +
  xlim(0,xUpperLim) + customTheme + middleline +
  annotate(geom = 'text', label = paste("Mean", avgs["OthUntr"], "%"),
           x = xtexts, y = ytexts) +
  scale_y_continuous(breaks = c(0,0.5,1), limits = c(0,1))

#A.combo <- ggarrange(pAllUntr, pAllBait, nrow=2,
# labels = c("A"), vjust = 1, common.legend = TRUE, legend = "bottom")

untr_smallplots <- ggarrange(AlgUntr, OomUntr, OthUntr, nrow = 3)
untr_smallplots_anot <- annotate_figure(untr_smallplots,
                                        left = text_grob("Rel. abundance",
                                                         rot = 90, size = 11))
bait_smallplots <- ggarrange(AlgBait, OomBait, OthBait, nrow = 3)
bait_smallplots_anot <- annotate_figure(bait_smallplots,
                                        left = text_grob("Rel. abundance",
                                                         rot = 90, size = 11))

combo <- ggarrange(pAllUntr, untr_smallplots_anot, pAllBait,
                   bait_smallplots_anot, nrow = 2, ncol = 2, widths = c(2,1),
                   labels = c("A", "", "B", ""), common.legend = TRUE,
                   legend = "bottom") + theme(plot.margin = margin(10,10,10,10))

#Save plot
save_plot(combo, plot_name = "Class_ranking_Fig3")

combo
```

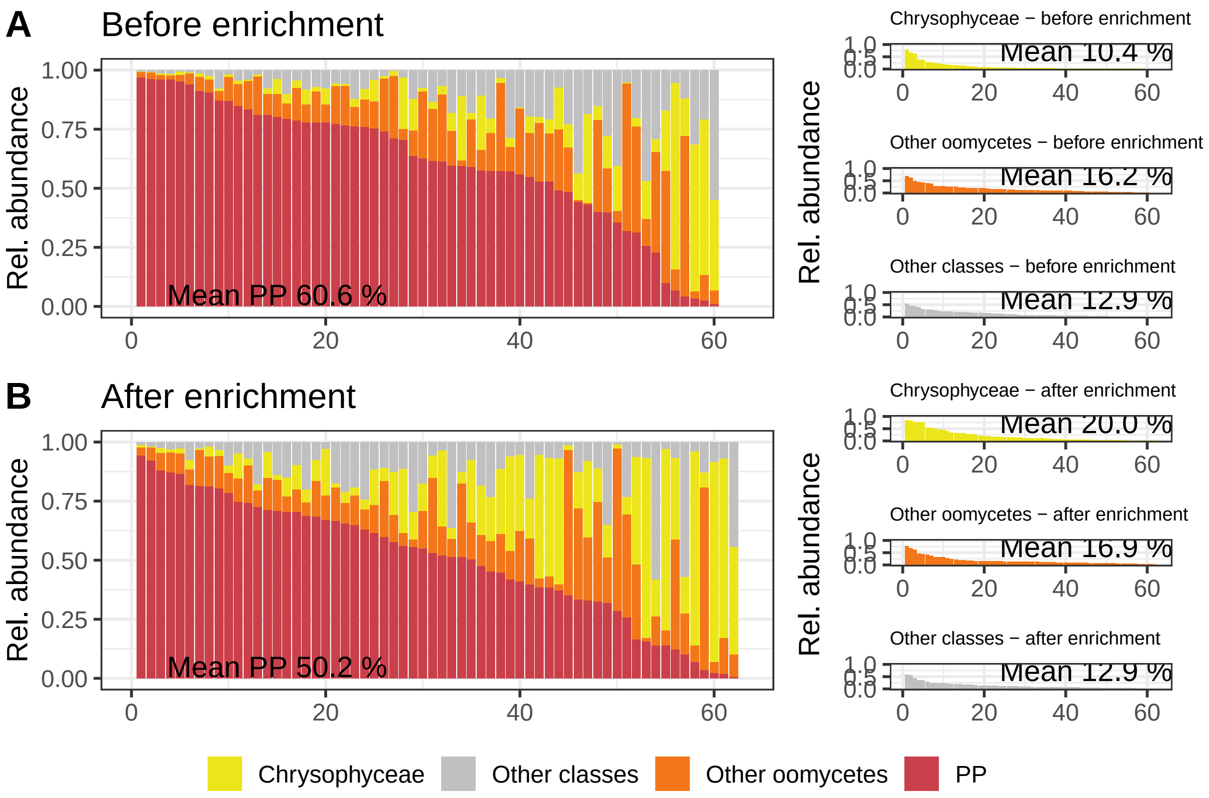


```
cat("Chunk successfully run")
```

```
## Chunk successfully run
```

Pairwise t-tests of classes in Fig. 3 This chunk documents the pairwise t-tests performed for the classes shown in Fig. 3. For each class/group of classes, the mean relative abundance before and after baiting are compared.

```
library(rstatix)
pairwise_test <- function(ASV_list){
  ps.temp <- prune_taxa(ASV_list, ps.trans)
  rankTemp <- cuphyr::make_ranked_sums(ps.temp)
  rankTemp <- as.data.frame(rankTemp)
  teststats <- pairwise_t_test(rankTemp, formula = Abundance ~ Bait)
  return(teststats)}

ChrysUntrVsBait <- pairwise_test(subASVsAlg)
subASVsO_P <- setdiff(subASVsOom, subASVsPP)
OomUntrVsBait <- pairwise_test(subASVsO_P)
PPUntrVsBait <- pairwise_test(subASVsPP)
subASVsOth <- setdiff(taxa_names(ps), subASVsOom)
subASVsOth <- setdiff(subASVsOth, subASVsAlg)
OthUntrVsBait <- pairwise_test(subASVsOth)
```

```r
all_stats <- list(PPUntrVsBait, ChrysUntrVsBait, OomUntrVsBait, OthUntrVsBait)
names(all_stats) <- c("PP", "Chrysophyceae", "Other oomycetes", "Other classes")
all_stats <- bind_rows(all_stats,.id = "Genera from")
all_stats
```

```
## # A tibble: 4 x 10
##   `Genera from` .y.   group1 group2    n1    n2       p p.signif   p.adj
##   <chr>         <chr> <chr>  <chr>  <int> <int>   <dbl> <chr>      <dbl>
## 1 PP            Abun~ Befor~ Enric~    60    62 0.0305  *        0.0305
## 2 Chrysophyceae Abun~ Befor~ Enric~    60    62 0.00818 **       0.00818
## 3 Other oomyce~ Abun~ Befor~ Enric~    60    62 0.793   ns       0.793
## 4 Other classes Abun~ Befor~ Enric~    60    62 0.983   ns       0.983
## # ... with 1 more variable: p.adj.signif <chr>
```

```r
cat("Chunk successfully run")
```

```
## Chunk successfully run
```

Comparison of Metabarcoding and baiting

Phylogenetic tree of isolates, ASVs and reference isolates This chunk generates a phylogeny for the isolates ITS sequences and corresponding ASVs and one reference sequence per identified species. The selection/similarity of ASVs was determined by vsearch, the fasta files were assembled with bash commands (Supplementary Fig. 4). This figure was fused with Fig.

```bash
# Trimming isolate sequences to the metabarcoding regions from Sanger
# consensus multifasta
cutadapt -a CGGAAGGATCATTACCAC...CAGCAGTGGATGTCTAGGCT --minimum-length 50 \
  -o trim_isos_v3.fasta Okay_Consensus_seqs_v3.fa

# Finding homologous ASVs using vsearch on a one-line-per-sequence
# (as opposed to multiline fasta) version of the ASV fasta with a similarity
# cutoff of 98%
vsearch --usearch_global trim_isos_v3.fasta -db ASVs_without_controls_oneline.fasta \
--userout match_trim-isolates_ASVs_v3.txt -userfields query+target+id+mism+opens \
-id 0.98 -strand both

# Parsing results into a list of unique ASVs found
sed 's/.*ASV/ASV/' match_trim-isolates_ASVs_v3.txt > exact_ASVs_v3.tmp
sed -i 's/\t.*//' exact_ASVs_v3.tmp
sort exact_ASVs_v3.tmp | uniq > exact_ASVs_v3.txt

# Removing temporary file
yes | rm exact_ASVs_v3.tmp

# Extracting fasta belonging to the unique ASVs from the oneline fasta file
grep -f exact_ASVs_v3.txt -A1  ASVs_without_controls_oneline.fasta \
> exact_ASVs_v3.fasta
sed -i '/--/d' exact_ASVs_v3.fasta

# Concatenating ASVs and trimmed isolate sequences into a multi-sequence fasta
```

```
cat exact_ASVs_v3.fasta trim_isos_v3.fasta > iso_and_ASVs_v3.fasta

# Modifying fasta headers for better display in the phylogenetic tree
sed -i 's/||.*//' iso_and_ASVs_v3.fasta
sed -i 's/@.*//' iso_and_ASVs_v3.fasta

# Trim reference BLAST results from with Genbank identifiers to the region of
# interest and concatenate with the isolate and ASV sequences
cutadapt -a CGGAAGGATCATTACCAC...CAGCAGTGGATGTCTAGGCT --minimum-length 50 \
-o BLAST_ref_trim.fa BLAST_ref.fasta
cat iso_and_ASVs_v3.fasta BLAST_ref_trim.fa > iso_ASVs_refs_v3.fasta
```

```r
wp <- 18
hp <- 13.5

seqs <- readDNAStringSet(file = file.path(path,"iso_ASVs_refs_v3.fasta"),
                         format = "fasta")
align <- AlignSeqs(DNAStringSet(seqs), iterations = 100, refinements = 5)
```

```
## Determining distance matrix based on shared 8-mers:
## ================================================================================
##
## Time difference of 0.12 secs
##
## Clustering into groups by similarity:
## ================================================================================
##
## Time difference of 0.04 secs
##
## Aligning Sequences:
## ================================================================================
##
## Time difference of 0.87 secs
##
## Iteration 1 of 100:
##
## Determining distance matrix based on alignment:
## ================================================================================
##
## Time difference of 0 secs
##
## Reclustering into groups by similarity:
## ================================================================================
##
## Time difference of 0.02 secs
##
## Realigning Sequences:
## ================================================================================
##
## Time difference of 0.33 secs
##
## Iteration 2 of 100:
##
## Determining distance matrix based on alignment:
```

```
## ================================================================================
##
## Time difference of 0 secs
##
## Reclustering into groups by similarity:
## ================================================================================
##
## Time difference of 0.01 secs
##
## Realigning Sequences:
## ================================================================================
##
## Time difference of 0.02 secs
##
## Alignment converged - skipping remaining iterations.
##
## Refining the alignment:
## ================================================================================
##
## Time difference of 0.22 secs
##
## Alignment converged - skipping remaining refinements.
```

```
seqs_phang <- phangorn::phyDat(as(align, "matrix"), type = "DNA")
seqs_dm <- phangorn::dist.ml(seqs_phang)
seqs_treeNJ <- NJ(seqs_dm)
seqs_fit = pml(seqs_treeNJ, data = seqs_phang)
fitGTRseqs <- update(seqs_fit, k = 4, inv = 0.2)
fitGTRseqs <- optim.pml(fitGTRseqs, model = "GTR", optInv = TRUE,
                        optGamma = TRUE, rearrangement = "stochastic",
                        control = pml.control(trace = 0))

GTR_tree <- treeio::as.treedata(fitGTRseqs$tree, type = "ml")
iso_ASV_tree <- ggtree(GTR_tree) + geom_tree() +
  geom_treescale(x = 0.5) +
  geom_tiplab(size = 3) + xlim(-0.1,3)

save_plot(iso_ASV_tree, plot_name = "IsolateSeqs_ASVs_ref_tree_Supfig4")
iso_ASV_tree
```

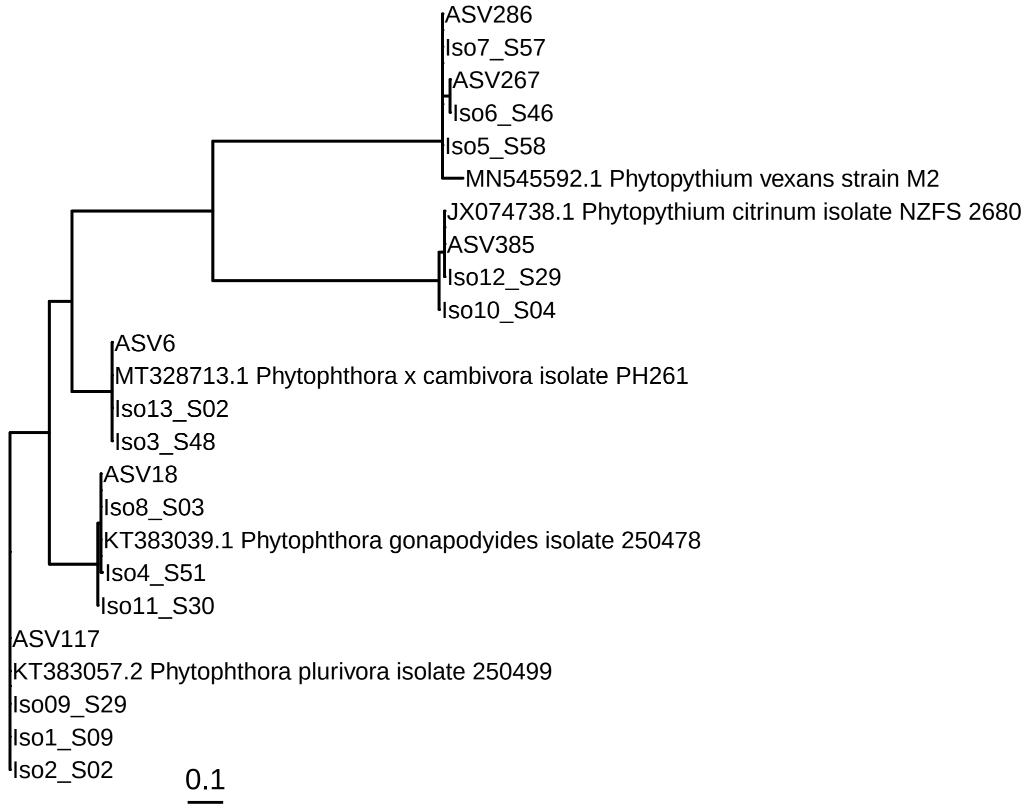


```r
cat("Chunk successfully run")
```

```
## Chunk successfully run
```

Isolates and corresponding ASVs This chunk generates a tile plot of the presence and abundance of ASVs that correspond with isolates in the soil samples before and after enrichment (Fig. 4). In revision 1 of the manuscript this figure was expanded by fusing it with the iso_ASV_tree generated in the previous chunk.

```r
##CHANGE ME to change the width (in cm) of the output.
wp <- 14
#CHANGE ME to change the height (in cm) of the output.
hp <- 18

# ASVs (before LULU!) that exactly match isolates seqs (identified by vsearch),
# read in from fasta
exASVs_seqs <- readDNAStringSet(paste0(path,"/exact_ASVs_v3.fasta"))
iso_seqs <- readDNAStringSet(paste0(path,"/trim_isos_v3.fasta"))
iso_ASV_match <- read_delim(paste0(path,"/match_trim-isolates_ASVs_v3.txt"),
                            delim = "\t",
                            col_names = c("Iso_Soil" ,"OTU",
                                          "Match", "Mism", "Gap"))

#Parse headers to get ASV IDs listed in vector
```

```
exASVs <- names(exASVs_seqs) %>% str_remove("\\|\\|.*")
isos <- names(iso_seqs)

#Re-label the facets
enrich_labs <- c("Before enrichment", "After enrichment")
names(enrich_labs) <- c("Before enrichment", "Enriched")

exASV_counts <- ps_tr_tbl %>%
  dplyr::select(OTU:Bait, Sanger, Rel_Abundance, Prcnt_Abundance) %>%
  dplyr::filter(OTU %in% exASVs) %>%
  dplyr::filter(Abundance >= 1)

iso_ASVs <- ggplot(exASV_counts, aes(x = OTU,
                                     y = reorder(Soil, dplyr::desc(Soil)),
                                     fill = Prcnt_Abundance)) +
              geom_tile() +
              scale_fill_viridis() +
              facet_wrap(~Bait, labeller = labeller(Bait = enrich_labs)) +
              theme(axis.text.x = element_text(angle = 90, hjust = 1, vjust = 0.5),
                    panel.grid = element_blank(),
                    axis.title = element_blank(),
                    strip.background = element_rect(fill = "white", color = NA)) +
              labs(fill = "Abund. (%)") +
              scale_x_discrete(limits = exASVs)

#save_plot(iso_ASVs, plot_name = "IsolateSeqs_ASVs_heatmap_Fig4")
#iso_ASVs

heatmap_tree_combo <- ggarrange(iso_ASVs, iso_ASV_tree, nrow = 2,
                                labels = c("A", "B"))

save_plot(heatmap_tree_combo, plot_name = "combined_tree_heatmap_Fig4_Rev1")
heatmap_tree_combo
```

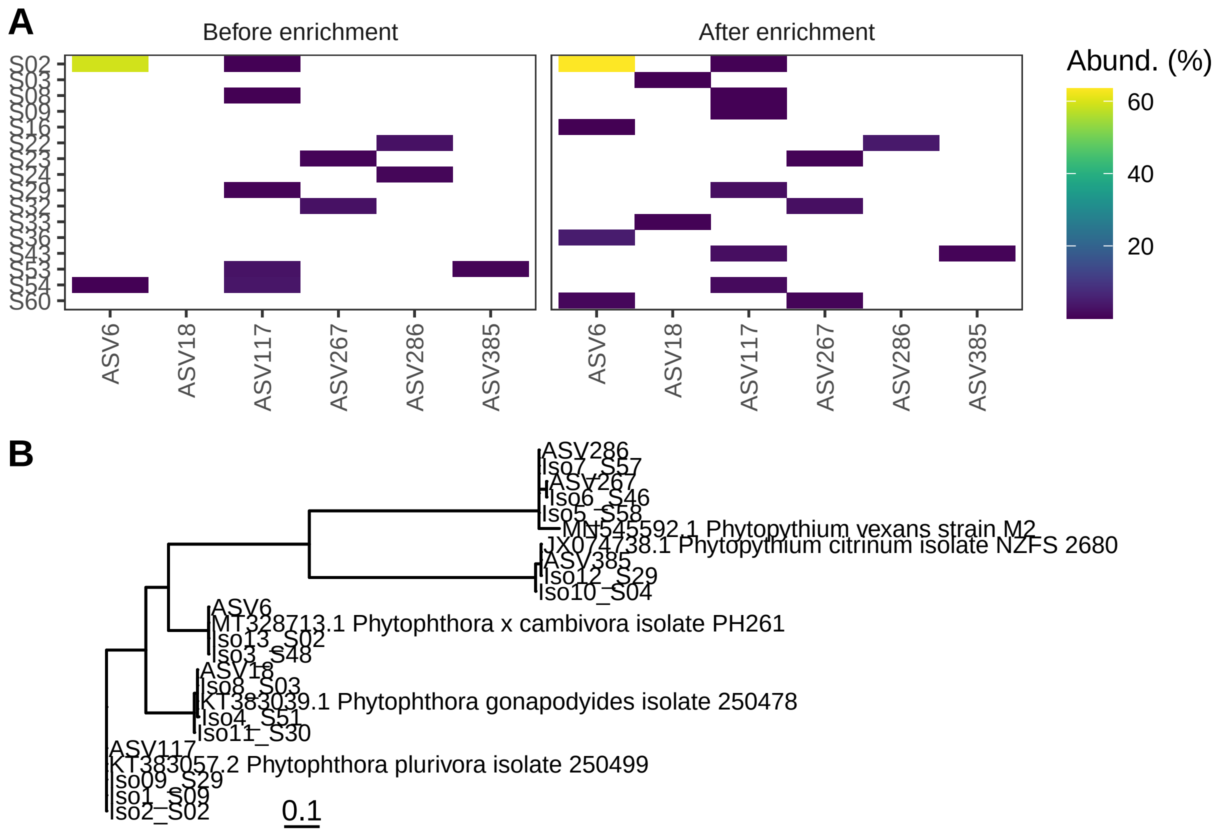


```
cat("Chunk successfully run")
```

```
## Chunk successfully run
```

Supplementary Fig4_Rev1 (previously Suppfig3) This figure is complementary to Fig 3 in the main manuscript but shows the absolute abundance for PP and Chrysophyceae instead of the relative abundances.

```
##CHANGE ME to change the width (in cm) of the output.
wp <- 17.5
#CHANGE ME to change the height (in cm) of the output.
hp <- 10
#CHANGE ME to change the resolution (in dpi) of the output.
res <- 150

#calculate averages for display in graphs
avgNames <- c("PPUntr", "PPBait","ChrUntr", "ChrBait")
avgs <- c(mean(ranksUntrPP_tot$Abundance),mean(ranksBaitPP_tot$Abundance),
          mean(ranksUntrAlgae_tot$Abundance),mean(ranksBaitAlgae_tot$Abundance))
names(avgs) <- avgNames
avgs <- format(round(avgs, 1), nsmall = 1)

#Define fixed color schemes for consistency between plots
plotvars <- c("Chrysophyceae_tot", "All oomycetes",
              "Other oomycetes", "Other classes", "PP_tot")
```

```r
getPalette <- colorRampPalette(viridis(length(plotvars)))
plotPalette <- getPalette(length(plotvars))
names(plotPalette) <- plotvars
plotPalette["Chrysophyceae_tot"] <- viridis_yellows[2]
plotPalette["PP_tot"] <- viridis_reds[4]

customTheme <- theme(legend.position = "none",
                     axis.title = element_blank(),
                     title = element_text(size = 7))
customThemeA <- theme(legend.position = "none",
                      legend.title = element_blank(),
                      legend.key.size = unit(5,"mm"),
                      axis.title = element_blank(),
                      title = element_text(size = 10))

xUpperLim <- nrow(ranksBaitAlgae_tot) + 1
ytextl = 0.05
xtextl = 15
ytexts = 90000
xtexts = 45

pAllBait <- ggplot(data = ranksBaitPP_tot,
                   aes(x = Rank, y = Abundance, fill = Set)) + geom_col() +
  scale_fill_manual(values = plotPalette) +
  ggtitle("PP - after enrichment") +
  xlim(0,xUpperLim) +
  customTheme +
  annotate(geom = 'text', label = paste("Mean", avgs["PPBait"]),
           x = xtexts, y = ytexts) +
  scale_y_continuous(limits = c(0,105000))

pAllUntr <- ggplot(data = ranksUntrPP_tot,
                   aes(x = Rank, y = Abundance, fill = Set)) + geom_col() +
  scale_fill_manual(values = plotPalette) +
  ggtitle("PP - before enrichment") +
  xlim(0,xUpperLim) +
  customTheme +
  annotate(geom = 'text', label = paste("Mean", avgs["PPUntr"]),
           x = xtexts, y = ytexts) +
  scale_y_continuous(limits = c(0,105000))

AlgBait <- ggplot(data = ranksBaitAlgae_tot,
                  aes(x = Rank, y = Abundance, fill = Set)) + geom_col() +
  scale_fill_manual(values = plotPalette) +
  ggtitle("Chrysophyceae - after enrichment") +
  xlim(0,xUpperLim) +
  customTheme +
  annotate(geom = 'text', label = paste("Mean", avgs["ChrBait"]),
           x = xtexts, y = ytexts) +
  scale_y_continuous(limits = c(0,105000))

AlgUntr <- ggplot(data = ranksUntrAlgae_tot,
                  aes(x = Rank, y = Abundance, fill = Set)) +
```

```
  geom_col() +
  scale_fill_manual(values = plotPalette) +
  ggtitle("Chrysophyceae - before enrichment") +
  xlim(0,xUpperLim) +
  customTheme +
  annotate(geom = 'text', label = paste("Mean", avgs["ChrUntr"]),
           x = xtexts, y = ytexts) +
  scale_y_continuous(limits = c(0,105000))

A.combo <- ggarrange(pAllUntr, pAllBait, nrow = 2, labels = c("A"), vjust = 1)
B.combo <- ggarrange(AlgUntr, AlgBait, nrow = 2, labels = c("B"), vjust = 1)

A.combo.anot <- annotate_figure(A.combo, left = text_grob("Abundance", rot = 90))

combo.AB <- ggarrange(A.combo.anot, B.combo, ncol = 2) +
  theme(plot.margin = margin(10,10,10,10)) +
  scale_fill_manual(values = plotPalette)


#Save plot
save_plot(combo.AB, plot_name = "Class_absolute_ranking_Supfig4")
combo.AB
```

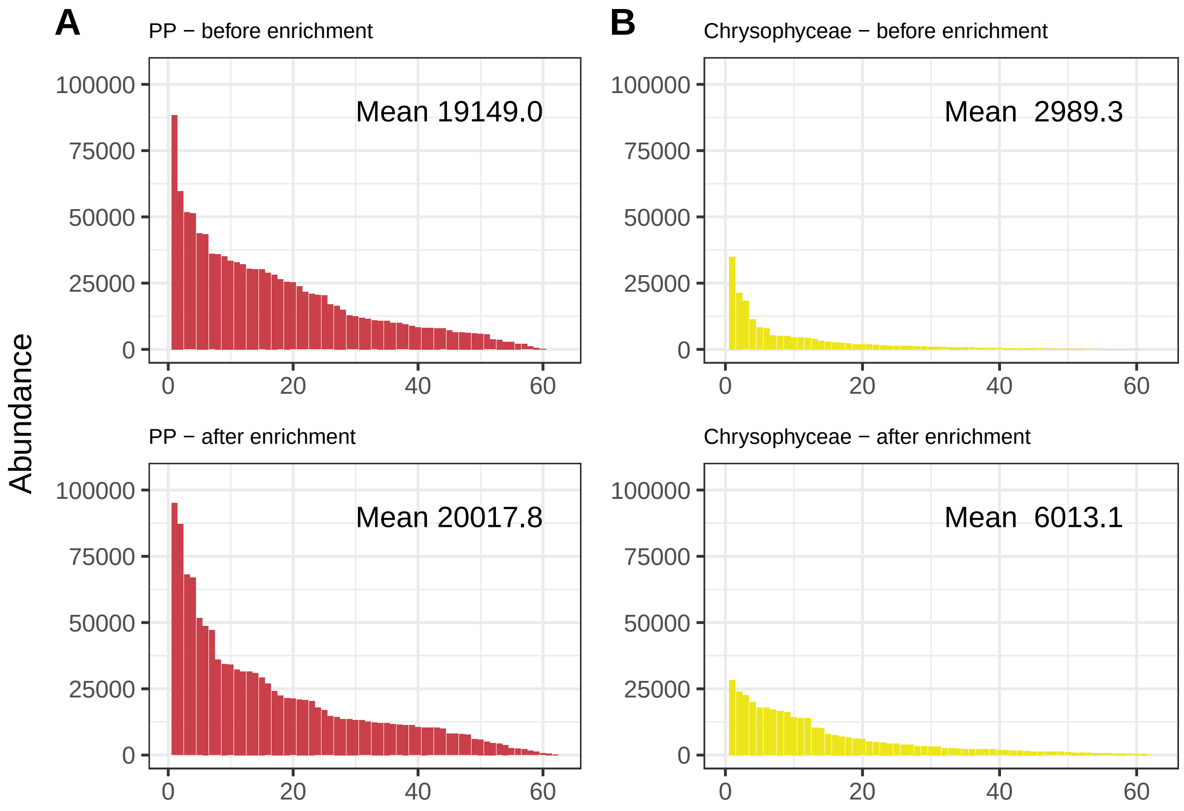


```
cat("Chunk successfully run")
```

```
## Chunk successfully run
```

```
sessionInfo()
```

```
## R version 4.0.2 (2020-06-22)
## Platform: x86_64-w64-mingw32/x64 (64-bit)
## Running under: Windows 10 x64 (build 17763)
##
## Matrix products: default
##
## locale:
## [1] LC_COLLATE=English_United Kingdom.1252
## [2] LC_CTYPE=English_United Kingdom.1252
## [3] LC_MONETARY=English_United Kingdom.1252
## [4] LC_NUMERIC=C
## [5] LC_TIME=English_United Kingdom.1252
##
## attached base packages:
## [1] stats4    parallel  stats     graphics  grDevices utils     datasets
## [8] methods   base
##
## other attached packages:
##  [1] rstatix_0.6.0       magrittr_1.5        data.table_1.13.2
##  [4] cuphyr_0.3          ggtree_2.2.4        treeio_1.12.0
##  [7] viridis_0.5.1       viridisLite_0.3.0   gridExtra_2.3
## [10] ggpubr_0.4.0        ggsci_2.9           DECIPHER_2.16.1
## [13] RSQLite_2.2.1       phangorn_2.5.5      ape_5.4-1
## [16] Biostrings_2.56.0   XVector_0.28.0      IRanges_2.22.2
## [19] S4Vectors_0.26.1    BiocGenerics_0.34.0 forcats_0.5.0
## [22] stringr_1.4.0       dplyr_1.0.2         purrr_0.3.4
## [25] readr_1.4.0         tidyr_1.1.2         tibble_3.0.4
## [28] ggplot2_3.3.2       tidyverse_1.3.0     dada2_1.16.0
## [31] Rcpp_1.0.5          phyloseq_1.32.0
##
## loaded via a namespace (and not attached):
##   [1] readxl_1.3.1             backports_1.2.0
##   [3] fastmatch_1.1-0          plyr_1.8.6
##   [5] igraph_1.2.6             lazyeval_0.2.2
##   [7] splines_4.0.2            BiocParallel_1.22.0
##   [9] usethis_1.6.3            GenomeInfoDb_1.24.2
##  [11] digest_0.6.27            foreach_1.5.1
##  [13] htmltools_0.5.0          fansi_0.4.1
##  [15] memoise_1.1.0            cluster_2.1.0
##  [17] openxlsx_4.2.3           remotes_2.2.0
##  [19] modelr_0.1.8             RcppParallel_5.0.2
##  [21] matrixStats_0.57.0       prettyunits_1.1.1
##  [23] jpeg_0.1-8.1             colorspace_1.4-1
##  [25] blob_1.2.1               rvest_0.3.6
##  [27] haven_2.3.1              xfun_0.19
##  [29] callr_3.5.1              crayon_1.3.4
##  [31] RCurl_1.98-1.2           jsonlite_1.7.1
##  [33] survival_3.2-7           iterators_1.0.13
##  [35] glue_1.4.2               gtable_0.3.0
##  [37] zlibbioc_1.34.0          DelayedArray_0.14.1
```

```
##  [39] car_3.0-10                  pkgbuild_1.1.0
##  [41] Rhdf5lib_1.10.1             abind_1.4-5
##  [43] scales_1.1.1                DBI_1.1.0
##  [45] progress_1.2.2              tidytree_0.3.3
##  [47] foreign_0.8-80              bit_4.0.4
##  [49] httr_1.4.2                  RColorBrewer_1.1-2
##  [51] ellipsis_0.3.1              farver_2.0.3
##  [53] pkgconfig_2.0.3             dbplyr_2.0.0
##  [55] utf8_1.1.4                  labeling_0.4.2
##  [57] tidyselect_1.1.0            rlang_0.4.8
##  [59] reshape2_1.4.4              munsell_0.5.0
##  [61] cellranger_1.1.0            tools_4.0.2
##  [63] cli_2.1.0                   generics_0.1.0
##  [65] ade4_1.7-16                 devtools_2.3.2
##  [67] broom_0.7.2                 evaluate_0.14
##  [69] biomformat_1.16.0           yaml_2.2.1
##  [71] processx_3.4.4              knitr_1.30
##  [73] bit64_4.0.5                 fs_1.5.0
##  [75] zip_2.1.1                   nlme_3.1-150
##  [77] aplot_0.0.6                 xml2_1.3.2
##  [79] compiler_4.0.2              rstudioapi_0.11
##  [81] curl_4.3                    png_0.1-7
##  [83] testthat_3.0.0              ggsignif_0.6.0
##  [85] reprex_0.3.0                stringi_1.5.3
##  [87] ps_1.4.0                    desc_1.2.0
##  [89] lattice_0.20-41             Matrix_1.2-18
##  [91] vegan_2.5-6                 permute_0.9-5
##  [93] multtest_2.44.0             vctrs_0.3.4
##  [95] pillar_1.4.6                lifecycle_0.2.0
##  [97] BiocManager_1.30.10         cowplot_1.1.0
##  [99] bitops_1.0-6                patchwork_1.0.1
## [101] GenomicRanges_1.40.0        R6_2.5.0
## [103] latticeExtra_0.6-29         hwriter_1.3.2
## [105] ShortRead_1.46.0            rio_0.5.16
## [107] sessioninfo_1.1.1           codetools_0.2-16
## [109] MASS_7.3-53                 assertthat_0.2.1
## [111] pkgload_1.1.0               rhdf5_2.32.4
## [113] SummarizedExperiment_1.18.2 rprojroot_1.3-2
## [115] withr_2.3.0                 GenomicAlignments_1.24.0
## [117] Rsamtools_2.4.0             GenomeInfoDbData_1.2.3
## [119] mgcv_1.8-33                 hms_0.5.3
## [121] quadprog_1.5-8              grid_4.0.2
## [123] rvcheck_0.1.8               rmarkdown_2.5
## [125] carData_3.0-4               Biobase_2.48.0
## [127] lubridate_1.7.9
```

Credit This script is based in part on ideas and code from the dada2 Tutorial by Benjamin Callahan, the publication "Bioconductor Workflow for Microbiome Data Analysis: from raw reads to community analyses" by Callahan et al. (2016) and various pages of the official phyloseq website by Paul J. McMurdie.
